# Catechol-O-methyl transferase suppresses cell invasion and interplays with MET signaling in estrogen dependent breast cancer

**DOI:** 10.1038/s41598-023-28078-1

**Published:** 2023-01-23

**Authors:** Lucia Janacova, Michaela Stenckova, Petr Lapcik, Sarka Hrachovinova, Pavla Bouchalova, David Potesil, Roman Hrstka, Petr Müller, Pavel Bouchal

**Affiliations:** 1 Department of Biochemistry, Faculty of Science, Masaryk University, Kamenice 5, 62500 Brno, Czech Republic; 2 Research Centre for Applied Molecular Oncology, Masaryk Memorial Cancer Institute, Brno, Czech Republic; 3 Proteomics Core Facility, Central European Institute for Technology, Masaryk University, Brno, Czech Republic

**Keywords:** Breast cancer, Cell invasion, Proteomics, Protein-protein interaction networks, RNA

## Abstract

Catechol-O-methyl transferase (COMT) is involved in detoxification of catechol estrogens, playing cancer-protective role in cells producing or utilizing estrogen. Moreover, COMT suppressed migration potential of breast cancer (BC) cells. To delineate COMT role in metastasis of estrogen receptor (ER) dependent BC, we investigated the effect of *COMT* overexpression on invasion, transcriptome, proteome and interactome of MCF7 cells, a luminal A BC model, stably transduced with lentiviral vector carrying *COMT* gene (MCF7-COMT). 2D and 3D assays revealed that COMT overexpression associates with decreased cell invasion (*p* < 0.0001 for Transwell assay, *p* < 0.05 for spheroid formation). RNA-Seq and LC-DIA-MS/MS proteomics identified genes associated with invasion (*FTO, PIR, TACSTD2, ANXA3, KRT80, S100P, PREX1, CLEC3A, LCP1*) being downregulated in MCF7-COMT cells, while genes associated with less aggressive phenotype (*RBPMS, ROBO2, SELENBP, EPB41L2*) were upregulated both at transcript (|log2FC|> 1, adj. *p* < 0.05) and protein (|log2FC|> 0.58, *q *< 0.05) levels. Importantly, proteins driving MET signaling were less abundant in COMT overexpressing cells, and pull-down confirmed interaction between COMT and Kunitz-type protease inhibitor 2 (SPINT2), a negative regulator of MET (log2FC = 5.10, *q* = 1.04^−7^). In conclusion, COMT may act as tumor suppressor in ER dependent BC not only by detoxification of catechol estrogens but also by suppressing cell invasion and interplay with MET pathway.

## Introduction

Estrogens are among very few aromatic molecules circulating in the human body and contribute to an elevated breast cancer risk involving estrogen receptor (ER)-mediated cell proliferation associated with spontaneous replication error. Likewise, a complementary pathway involving indirect and direct genotoxicity caused by estrogen metabolites, such as 16α-hydroxyestrone, catechol estrogens and estrogen quinones participate in the mechanism^1^.

Catechol-O-methyl transferase (COMT) occurs in variable amounts in most tissues such as endometrium and mammary gland^2,3^ and plays an essential role in detoxification of catechol substrates by transferring a methyl group from *S*-adenosyl-*L*-methionine (SAM) to one of the hydroxyls in a molecule of a substrate^2^. Along with NAD(P)H-quinone oxidoreductase (NQO1), it is quantitatively the most significant contributor to inactivation of catechol estrogens^4^, intermediates in the formation of reactive oxygen species causing different types of DNA damage in breast tissue^1,5,6^. COMT blocks estrogenicity of catechol metabolites of estradiol and estrone, particularly 2-hydroxy and 4-hydroxy estradiol/estrone, and prevents their oxidation to reactive quinones^4,7^. On top of that, methoxyestrogens formed by COMT, especially 2-methoxyestradiol, also have tumor-suppressing properties and cardioprotective effects mediated through antiangiogenic and growth inhibitory effects. Hence, COMT generates a potentially protective metabolites along with blocking genotoxic effects of catechol estrogens^4,8^.

Therefore, COMT has become a research target as a possible tumor suppressor in breast cancer. Studies have focused on its functional polymorphisms which have been considered to regulate COMT catalytic activity and have the impact on its protective effect, mainly the Val108/158Met polymorphism, posing rather inconclusive results^1,9,10^.

In our previous study, we found a positive association between COMT protein level and cell migration of triple negative MDA-MB-231 cancer cells, as well as connection of COMT protein levels with lymph node metastasis, high grade and poor survival of triple negative breast cancer patients via SWATH-MS proteomics^11^.

It is evident that previous studies led to inconclusive results regarding the general role of COMT in breast cancer development, suggesting the role being dependent on ER status. Here we study its role in the model of ER dependent breast cancer subtype, luminal A. Using invasion studies in 2D and 3D setting we show that COMT overexpression is connected with lower invasiveness, being supported by RNA-Seq and DIA-based proteomics analysis^12^ recognizing changes relevant for organization of extracellular matrix, MET signaling, and transcription. In addition, discovery of a novel interaction partners including Kunitz-type protease inhibitor SPINT2, pointed to the novel mechanism of tumor suppressor role of COMT in ER dependent breast cancer through the interplay with MET signaling pathway.

## Materials and methods

### Cell line preparation and cultivation

The MCF7 breast cancer cell line was purchased from ATCC. MCF7 breast cancer cells were stably transduced with lentiviral vectors (i) pLENTI6.3-RBS-COMT-IRES-EmGFP-GWs expressing COMT (without tag) and green fluorescence protein (GFP) (MCF7-COMT), (ii) pLENTI6.3-EmGFP-GWs expressing GFP as a control (MCF7-GFP), (iii) pLENTI6.3-N-SBP-TEV-COMT-IRES-EmGFP-GWs expressing COMT N-terminally tagged with streptavidin binding peptide and GFP (MCF7-SBP-COMT) and (iv) pLENTI6.3-N-SBP-EmGFP-GWs expressing N-terminal SBP and GFP (MCF7-SBP) as described in Supplementary Methods. The parental MCF7 cells were cultured in Dulbecco's Modified Eagle´s Medium (DMEM; high glucose) with 10% fetal bovine serum (Merck, Germany) at 37 °C in 5% CO_2_ atmosphere; the stably transduced MCF7 cells were cultured under the same conditions except for adding blasticidin (10 μg/ ml; InvivoGen, USA) to the culture medium after the first passage.

### Transwell assay

Cells were cultured in five 6 cm culture dishes per cell line to ~ 80% confluency. Prior to the harvest, the cells were starved in serum-free DMEM medium for 6 h. The cells were then trypsinized, counted, and 100,000 cells were seeded per Transwell well. Invasiveness of the cells was determined by Transwell 96-well plate (Corning Inc., USA) according to the manufacturer’s protocol in three independent biological experiments. Nine replicates (wells) were used for each condition (with and without FBS) (see Supplementary Fig. S1 for a detailed design of Transwell assay and calibration curves). The top chambers were coated with basement membrane extract (BME, Cultrex® Trevigen, USA) diluted in Coating Buffer (1:50, Trevigen). Invading cells were detected using a fluorescent dye Calcein-AM. The plate was read using a TECAN reader (Tecan Infinite 1000 Pro, Tecan, Switzerland) in fluorescence top reading mode with 16 reads per well and gain set to 100. A standard curve of the dependence of relative fluorescence on cell number was constructed, and percentual invasiveness was calculated from the number of cells which invaded to the bottom chamber of each well divided by the initial number of plated cells (100,000 cells).

### 3D spheroid cell invasion assay, confocal laser scanning microscopy and image analysis

The cells were cultured in 6 cm culture dishes to ~ 80% confluency in duplicates per condition. Then the cells were trypsinized, counted, and 4000 cells were resuspended in Spheroid formation matrix solution and pelleted in a 96-well Round bottom plate in triplicates for each cell line. Cell invasiveness in 3D setting was analyzed using the Cultrex 96 Well 3D Spheroid BME Cell Invasion Assay (Trevigen) according to the manufacturer’s instructions. Serum-supplemented invasion matrix and complete DMEM medium with 5 µM SIR-DNA^13^ cell dye (Spirochrome, Switzerland) were added to each well 3 days after seeding the cells. Images were acquired using confocal laser scanning microscopy every 24 h. Fresh DMEM medium with 5 µM SIR-DNA was added to each well 3 days after adding invasion matrix to the wells.

Confocal laser scanning microscopy was performed on a Zeiss LSM800 microscope with Plan-Apochromat 5X/0.16 objective. SIR-DNA was excited with 640 nm laser beam and fluorescent emission between 640 and 700 nm was captured on GaAsP-PMT detector. Confocal pinhole was fixed to 1 AU. Transmitted light detector (TL-PMT) was used to acquired overall morphology of spheroids. 3D images of spheroids were generated from Z-stack with fixed slice width and thickness (0.976 µm, 20.970 µm), and analyzed using Imaris 9.7.2 software (Bitplane AG, Oxford Instruments, UK)^14^. Volumes of spheroids were quantified based on SIR-DNA intensity and standard surface detection. Volume measurements were carried out in triplicates. Three independent experiments were performed.

### Protein detection by SDS-PAGE and western blotting

Cell lysates for SDS PAGE were prepared using hot (95 °C) complete sample buffer (14.5% glycerol, 87 mM Tris HCl pH 6.8, 0.006% bromophenol blue in 1 M Tris HCl pH 6.8, 2.9% sodium dodecyl sulfate, 10% mercaptoethanol) and stored at − 20 °C. SDS-PAGE apparatus (Mini-PROTEAN® 3, Bio-Rad, USA) was used with a 5% stacking gel and 10% running gel. 30 µg of protein lysates as determined by an RC-DC Protein Assay (Bio-Rad), and Precision Plus Protein Dual Color Standards (Bio-Rad) were run in electrophoretic buffer (25 mM Tris Base, 192 mM glycine, 0.1% SDS) at 30 mA/gel and wet-transferred onto nitrocellulose membranes (BioTrace NT, 0.22 μm, Pall Life Sciences, Mexico) in a blotting buffer (20% methanol, 192 mM glycine, 24.8 mM Tris Base) at 100 V for 75 min. Membranes were then cut into pieces according to molecular weight of target protein at corresponding bands of Mw marker and blocked for 1 h in PBS + 0.1% Tween 20 (2.68 mM KCl, 137 mM NaCl, 6.45 mM Na_2_HPO_4_*12H_2_O, 1.47 mM KH_2_PO_4_, 0.89 mM Tween 20) containing 5% fat-free dried milk powder (PanReac Applichem, Germany) and incubated with primary antibody at 4 °C overnight. The following antibodies were used: rabbit anti-COMT (Abcam, UK; ab129504, 1:10,000); mouse anti-actin (Merck; A3853, 1:1000) and anti-SBP (Merck; MAB10764, 1:2000). After incubation, membranes were washed twice in PBS (phosphate buffered saline, 137 mM NaCl; 2.68 mM KCl; 1.47 mM KH_2_PO_4_; 10 mM Na_2_HPO_4_, pH 7.4) and twice in PBS + 0.1% Tween 20 and subsequently incubated with the corresponding secondary antibody (RAMPx, Dako, Denmark; P0161, 1:1000; SWARPx, Dako; P0217, 1:1000) at room temperature for 1 h and washed again. After 5 min of incubation of membranes with enhanced chemiluminescence (ECL) solution (10 mM luminol, 0.5 mM EDTA, 405 μM coumaric acid, 200 mM Tris pH 9.4, 8 mM sodium perborate tetrahydrate, 50 mM sodium acetate), ECL of immunoreactive proteins were visualized using a CCD camera (Fusion FX 7, Vilber Lourmat, France).

### Sample preparation for RNA-Seq analysis

The cells were cultured in 6 cm culture dishes to ~ 80% confluency in duplicates for each cell line. The cells were washed two times with cold PBS on ice, harvested by adding 500 µl of PBS and scraping by a cell scraper, transferred to a microtube and stored on ice. After centrifuging the lysates (1000 g, 5 min, 4 °C,), the supernatant was aspirated and 360 µl of TRI reagent (Merck) was added. Total RNA was isolated according to TRI reagent protocol and its concentration was determined using Qubit RNA BR assay kit (Thermo Fisher Scientific, USA). 240 ng of total RNA at 20 ng/µl was used in RNA-Seq analysis. Samples were stored at − 80 °C.

### RNA-Seq analysis and data processing

The TruSeq Stranded Total RNA LT Sample Prep Kit (Illumina, USA) was used to convert 0.5 mg of total RNA into a library of template molecules. Library was validated using Bioanalyzer (DNA 1000 Kit, Agilent Technologies, USA) and quantified according to manufacturer instructions by qPCR (KAPA Library Quantification Kit Illumina platforms, Kapa Biosystems, USA) using Quant studio (QuantStudio 5, Thermo Fisher Scientific). Samples were sequenced using NextSeq 500 (Illumina).

For RNA-seq, the raw reads were filtered to remove the adaptors and low-quality bases using Trimmomatic (v0.36) with Truseq2 as well as any reads that were shorter than 65 bases. Filtered reads were aligned to the human genome (Homo_sapiens.GRCh38.dna.primary_assembly) using STAR (v2.5.2b) in end-to-end mode to scan splice junctions. Then the counts in exon genomic features were calculated subread (v1.5.2). Differential expression analysis was performed in R 3.5.3 under the Deseq2 package version 1.22.2. BiomaRt package version 2.38.0. was used for annotation the results from differential expression analysis.

### Sample preparation for total proteome analysis

The cells were cultured in 6 cm culture dishes to ~ 80% confluency in triplicates for each cell line. The cells were washed two times with cold PBS on ice and lysed using 200 µl of lysis buffer (6 M guanidine hydrochloride, 100 mM Na-phosphate pH 6.6, 1% Triton X-100). Lysed cells were harvested by a cell scraper, transferred to microtube, and stored on ice. Subsequently, the lysed cells were needle sonicated on ice (50 W, 30 × 0.1 s, 30 s pause, 30 × 0.1 s). After 75-min incubation at room temperature, cell lysates were centrifuged (14,000 g, 20 min, 4 °C) and the supernatant was collected. The protein concentration of cell lysates was determined using RC-DC protein assay kit (Bio-Rad). Samples were stored at − 80 °C.

### Sample preparation for pull-down assay and interactome analysis

The cells were cultured in three 15 cm culture dishes to ~ 80% confluency for each cell line in triplicates. Then they were harvested by a cell scraper, centrifuged (1000 g, 4 °C, 5 min), washed with PBS, and the cell pellets were frozen at − 80 °C. The frozen cell pellet was resuspended with HNN-lysis buffer (0.5% NP40, 200 mM Na_3_VO_4_, 1 mM PMSF, 1.2 μM avidin, Complete protease inhibitors without EDTA (Roche, Switzerland)) and subsequently pipetted to dissolve and avoid foaming. The suspension was incubated on ice for 10 min, then transferred to a 2 ml microtube and centrifuged at 14,000 g for 20 min at 4 °C. 250 μl of lysis buffer was added to Bio-Rad Spin Column (Bio-Rad, cat. No 732-6008) to avoid the formation of air bubbles. 100 μl of High Capacity Streptavidin Agarose Resin (Thermo Fisher Scientific, cat. No 20359) were mixed in 750 μl of HNN-lysis buffer, and 200 μl of the prepared beads were added to samples which were then incubated for 15 min at 4 °C on a rotary wheel. The beads were then washed twice with 1 ml of HNN-lysis buffer using Bio-Rad Mini Columns. After washing with lysis buffer, samples were washed three times with 1 ml of HNN buffer (50 mM HEPES, 150 mM NaCl, 50 mM NaF), which contained no detergent and inhibitors. Finally, samples were eluted with 200 μl of 2.5 mM Biotin in HNN buffer three times.

### Protein digestion and peptide purification

Protein digestion in cell lysates was performed on Filter Aided Sample Preparation (FASP) columns (Microcolon filter device, 30 kDa cut-off, Merck). 100 μg of cell lysate was added on the column, the samples were then centrifuged (14,000 g, 30 min, 20 °C). Both steps were repeated until all the volume of the pull-down eluate was applied. 100 μl of 8 M urea in 0.1 M Tris/HCl, pH 8.5, and 20 μl of 100 mM TCEP (tris (2-carboxyethyl) phosphine) was added on the filter, the proteins were reduced in a thermomixer (600 rpm, 30 min, 37 °C) and centrifuged (14,000 g, 15 min, 20 °C). Then, 100 μl of 8 M urea and 20 μl of 300 mM iodoacetamide were added to the samples. The samples were alkylated in a thermomixer (600 rpm, 1 min, 25 °C), stored without previous stirring in the dark (20 min) and centrifuged (14,000 g, 15 min, 20 °C). 100 μl of 100 mM NH_4_HCO_3_ were added on the filter and the sample was centrifuged (14,000 g, 20 min, 20 °C). The previous step was repeated once more. Proteolytic digestion of proteins was initiated by the addition of 100 μl of 50 mM NH_4_HCO_3_ and 3.33 μl solution of trypsin (Promega, USA) dissolved in 50 mM acetic acid at 1 μg/μl (trypsin: cleaved protein ratio 1:30). Samples were mixed in a thermomixer (600 rpm, 1 min, 37 °C) and cleaved overnight (37 °C, without shaking). On the next day, the peptides were eluted by centrifugation (14,000 g, 30 min, 20 °C) prior to desalting on MicroSpin C18 columns (The Nest Group, Inc., USA) as previously described^15^. The eluates were dried in SpeedVac (Thermo Fisher) and stored at − 20 °C.

### LC–MS/MS analyses

For the total proteome analysis, the dried peptides were solubilized using 50 µl of 2.5% formic acid (FA) in 50% ACN and 100 µl of pure acetonitrile and concentrated using SpeedVac concentrator (Thermo Fisher Scientific) to 20 µl. Finally, the concentrated samples were diluted into LC–MS vials to get peptide concentration of 1 µg/µl with addition of 1 µl of 0.01% polyethylene glycol in water, 1 µl of stock iRT peptides standard (Biognosys, Switzerland), 2 µl of 5% FA and filled into 10 μl by MilliQ water (Merck). Two µg of peptides mixture was injected for each sample.

LC–MS/MS analyses were done using RSLCnano system online connected to Orbitrap Fusion Lumos tribrid mass spectrometer (Thermo Fisher Scientific, USA). Prior to the LC separation, tryptic digests were online concentrated and desalted using trapping column (100 μm × 30 mm) filled with 3.5 μm X-Bridge BEH 130 C18 sorbent (Waters, USA). After washing of trapping column with 0.1% FA, the peptides were eluted from the trapping column onto analytical Acclaim Pepmap100 C18 column (3 µm particles, 75 μm × 500 mm; Thermo Fisher Scientific, USA) by the following gradient program (mobile phase A: 0.1% FA in water; mobile phase B: 0.1% FA in 80% ACN; flow 300 nl/min): the gradient elution started at 5% of mobile phase B and began to increase in the 5th min to 37% during the 109 min, then reached to 80% of mobile phase B in the next 6 min and remained at this state for the last 10 min. Equilibration of the trapping column and the analytical column was done prior to sample injection to sample loop. The analytical column outlet was directly connected to the Digital PicoView 550 (New Objective, USA) ion source. ABIRD (Active Background Ion Reduction Device, ESI Source Solutions) was installed. MS data were acquired in a data-independent mode (DIA).

Orbitrap analyzer and quadrupole mass filter were employed for survey scan detection (350–1650 m/z). The MS scan resolution was 120,000 (at 200 m/z) with a target value of 2*10^5^ ions and maximum injection time of 100 ms. After the MS scan, defined m/z segments were isolated by quadrupole mass filter and HCD fragmentation was done with a target value of 5*10^5^ ions. MS/MS spectra after HCD fragmentation (default charge state is 2 and 28% collision energy) were recorded in Orbitrap with a resolution of 30,000 (at 200 m/z) in scan range of 200 – 1800 m/z. The maximum injection time for MS/MS was 50 ms.

For the interactome analysis, the dried peptides were analyzed in DIA mode as previously described^16^ with several modifications as described in Supplementary Methods.

### Proteomics data analysis and omics data integration

DIA data were analyzed in Spectronaut software version 13.9 (Biognosys)^17^ in directDIA mode. UniProt/SwissProt database version 2019_07 downloaded on 2019-09-16 limited to human entries containing 20,431 sequences was used for database search. Carbamidomethylation (C) was used as fixed modification, oxidation (M) and acetylation (protein N-term) were used as variable modifications. *q*-value at both precursor and protein levels were set to 0.01. Global data normalization was performed, data based on *q*-value 0.5 percentile (identified in 3 of 6 total runs) were involved in the final dataset. Analysis of differential protein abundance was performed using t-test implemented in Spectronaut^17^ with false discovery rate correction. Default settings were used for other parameters. Omics data integration analysis was performed in in Bioconductor Mixomics package version 3.14 under R 4.0.5.

Out of all proteins revealed by pull-down assay as interaction partners of COMT (log2FC (control/COMT) > -1 and *q*-value < 0.05), the true positive interaction partners were selected by filtering the proteins with significantly increased protein levels (log2FC > 0 and *q* < 0.05) in total proteome analysis out. The resulting true positive protein interaction partners of COMT were displayed by Cytoscape software (version 3.7.2)^18^.

### Gene set enrichment analysis

Gene set enrichment analysis (GSEA) in GSEA Java desktop application (http://software.broadinstitute.org/gsea/downloads.jsp) version 4.2.3 was conducted using the log2 fold change pre-ranked list of all quantified proteins from whole proteome and RNA-seq analyses (COMT vs. control) to identify enriched pathways. All the analyses were conducted using a priori defined pathways from Reactome database version 7.5.1 (ftp.broadinstitute.org://pub/gsea/gene_sets/c2.cp.reactome.v7.5.1.symbols.gmt). No chip platform was selected, otherwise default settings were used. Furthermore, log2 fold change pre-ranked list of all interaction partners of COMT insignificantly over- or lower-expressed in the whole proteome data was used to identify positively enriched pathways in pull-down data. Another GSEA analysis of COMT interaction partners with the same data with addition of COMT was carried out to remove interaction partners that are part of the same pathways as COMT. Minimal size of a gene set was adjusted to 1 and no chip platform was selected, otherwise default settings were used. Pathways with nominal *p*-value < 0.05 were considered as statistically significant. To visualize the overlap among enriched pathway in transcriptomic and proteomic data as an network, we used EnrichmentMap^19^ app in Cytoscape software (3.7.2). Node cutoff was adjusted to 0.1 of FDR *q*-value and for edge cutoff was selected combined Jaccard and Overlap metric at 0.375.

### Statistical analysis of cellular studies in vitro

The data were analyzed using one-way ANOVA with the Sidak’s multiple comparisons test in Prism 8.4.0 (GraphPad Software, USA)^20^, the differences were considered significant at *p* < 0.05. Data are presented as medians with box extended from the 25th to the 75th percentile and whiskers representing minimal and maximal value in the dataset in the cumulative graphs based on three independent biological experiments in main article and individual graphs representing biological replicates in Supplementary Information.

## Results

### COMT overexpression decreases invasiveness of MCF7 cells in 2D and 3D setting

**Figure 1:**
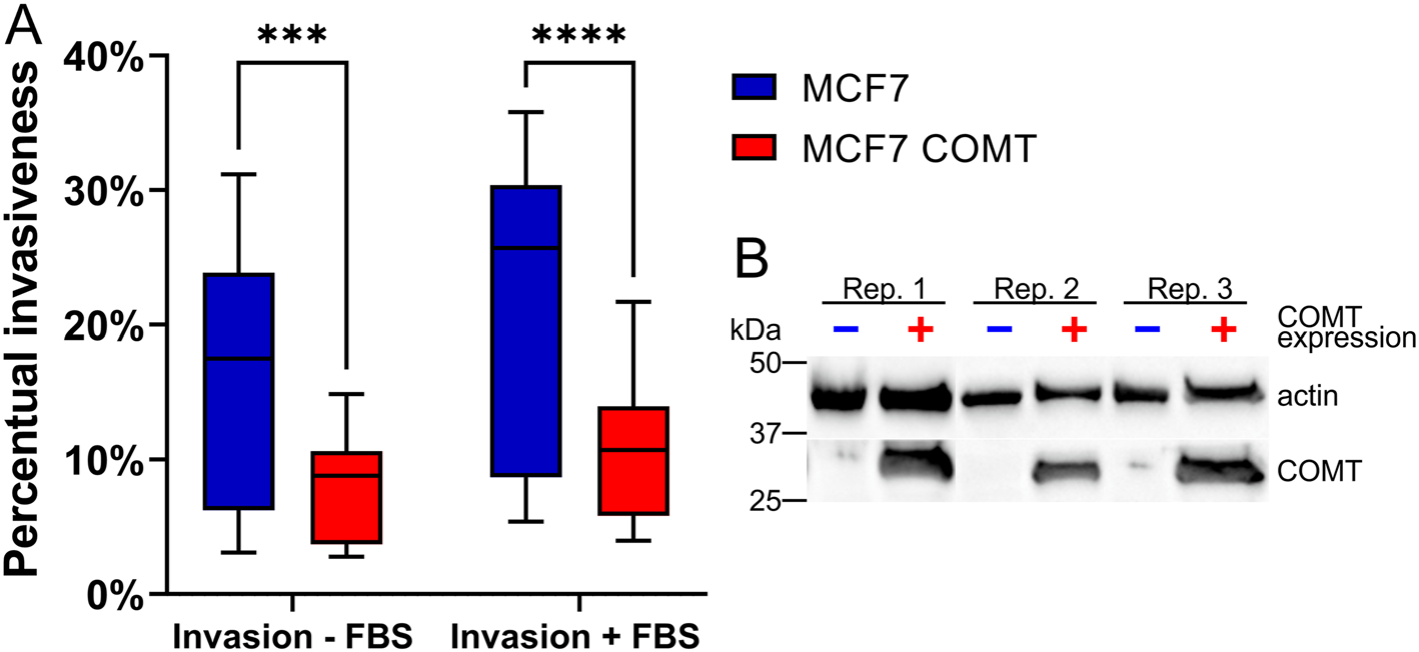
2D invasion Transwell assay of COMT overexpressing MCF7 cells (MCF7-COMT) compared to parental MCF7 cells. (**A**) Box plot of the percentual invasiveness of the cells with and without the presence of chemoattractant (fetal bovine serum, FBS) (n = 9 per cell line). **** *p* < 0.0001, *** *p* < 0.001. Boxes are extended from the 25th to the 75th percentile, with a line at the median. The whiskers represent minimal and maximal value in the dataset. The data are cumulative for three independent Transwell experiments. See Fig. S1 for the design of Transwell assay and the calibration curves. See Fig. S3 for results of three individual biologically independent experiments. (**B**) Western blot analysis of COMT and actin expression in MCF7-COMT and MCF7 parental cells in three biologically independent experiments. See Fig. S2 for raw immunoblotting images.

To study the role of COMT in metastatic potential of ER dependent BC at cellular level, we examined how COMT overexpression affects cell invasiveness of MCF7 cells originally derived from luminal A breast tumor. We generated a new stably transduced MCF7 cell line overexpressing COMT (MCF7-COMT) and compared its invasiveness with that of parental MCF7 cells in 2D setting using Transwell invasion assay (Fig. 1 and Supplementary Fig. S2). Although MCF7 cells generally do not exhibit highly invasive phenotype, three independent biological experiments showed that MCF7-COMT cells exhibit significantly lower invasiveness than parental MCF7 cell line (*p* < 0.0001; Fig. 1 and Supplementary Fig. S3).

**Figure 2:**
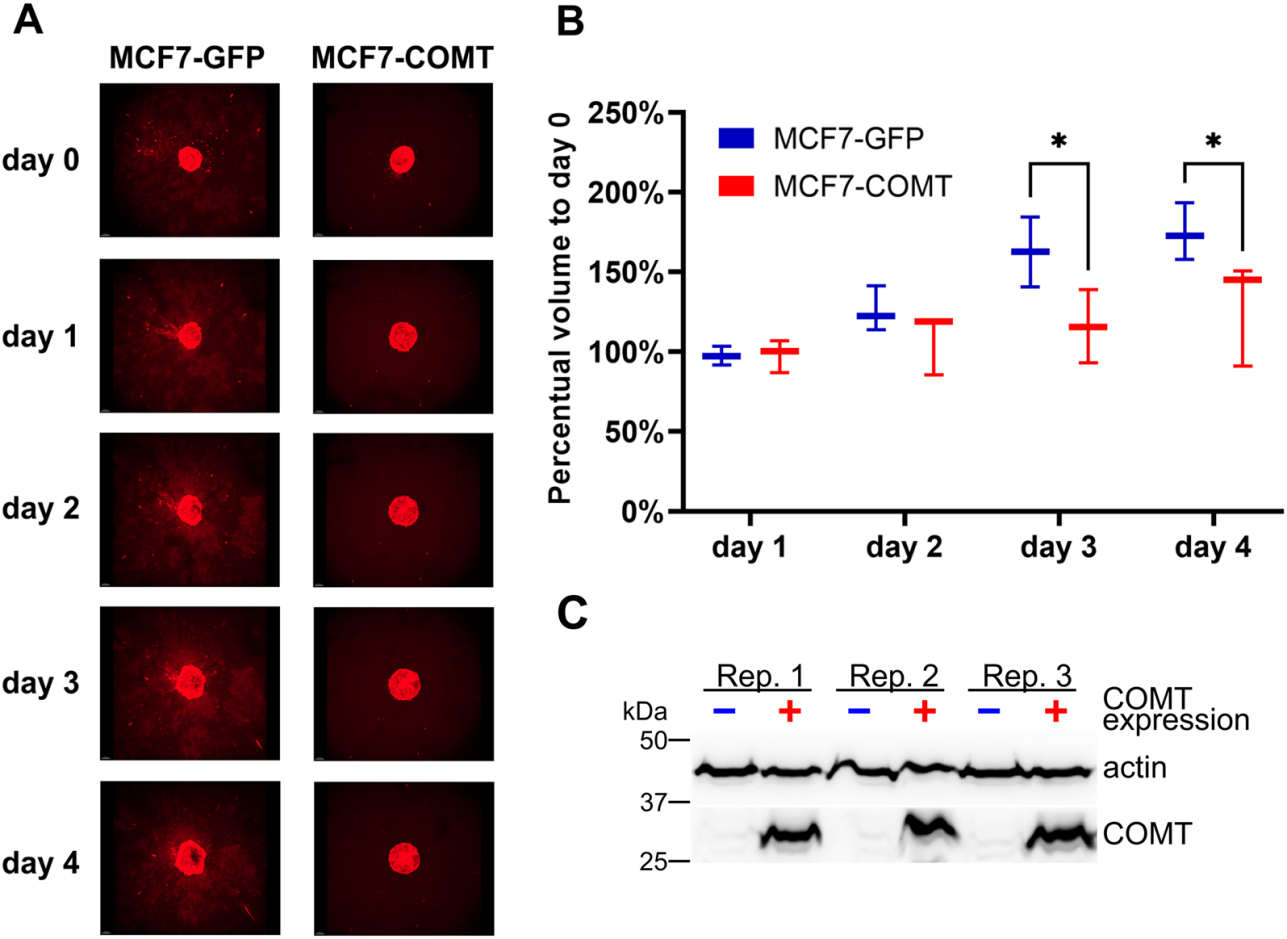
3D spheroid invasion assay of COMT overexpressing MCF7 cells (MCF7-COMT) compared to control MCF7-GFP cells. (**A**) Z-stack images of spheroids formed by MCF7-COMT cells and control cell line have been acquired by confocal laser scanning microscopy for 4 days/96 h after day 0. Data for single representative replicate, see Supplementary Fig. S4 for two remaining replicates and Supplementary Data S1 for full-resolution images. (**B**) Box plot of the percentual change in volume of the spheroids for all replicates (n = 3 per cell line). Boxes are extended from the 25th to the 75th percentile, with a line at the median. The whiskers represent minimal and maximal value in the dataset. *p < 0.05. (**C**) Western blot analysis of COMT and actin expression in MCF7-COMT and MCF7-GFP cells in three biologically independent experiments. See Fig. S5 for raw immunoblotting image.

To confirm this observation in 3D setting, we studied the effect of COMT overexpression on the invasiveness of MCF7 cells using a spheroid cell invasion assay. The 3D experiment confirmed decreased invasiveness of MCF7-COMT cells compared to control MCF7-GFP cells: cells did not invade into the surrounding invasion matrix (Fig. 2A,B, Supplementary Figs. S4, S5, Supplementary Data S1) and, in addition, spheroids formed by MCF7-COMT cells increased their volume significantly slower than control cells (*p* < 0.05; Fig. 2C).

Overall, COMT overexpression in MCF7 cells reduced cell invasiveness in comparison with both parental and mock cells in 2D and 3D settings, indicating anti-invasion role of COMT in ER dependent breast cancer.

### COMT overexpression modulates gene products associated with cell migration and adhesion and interplays with MET pathway

To understand the molecular background of COMT role in MCF7 cells, we analyzed stably transduced MCF7-COMT cells compared to control mock MCF7-GFP cells using RNA-Seq and proteomics based on LC-DIA-MS/MS. In total, 15,996 protein coding transcripts were quantified in RNA-Seq analysis (Supplementary Data S2) and 3465 protein groups represented by 28,681 peptides and 37,556 precursors were quantified in total proteome analysis (Supplementary Data S3). Of these, 3299 proteins groups matched their encoding genes in the human genome database used for RNA-Seq data analysis (Supplementary Data S4).

**Figure 3:**
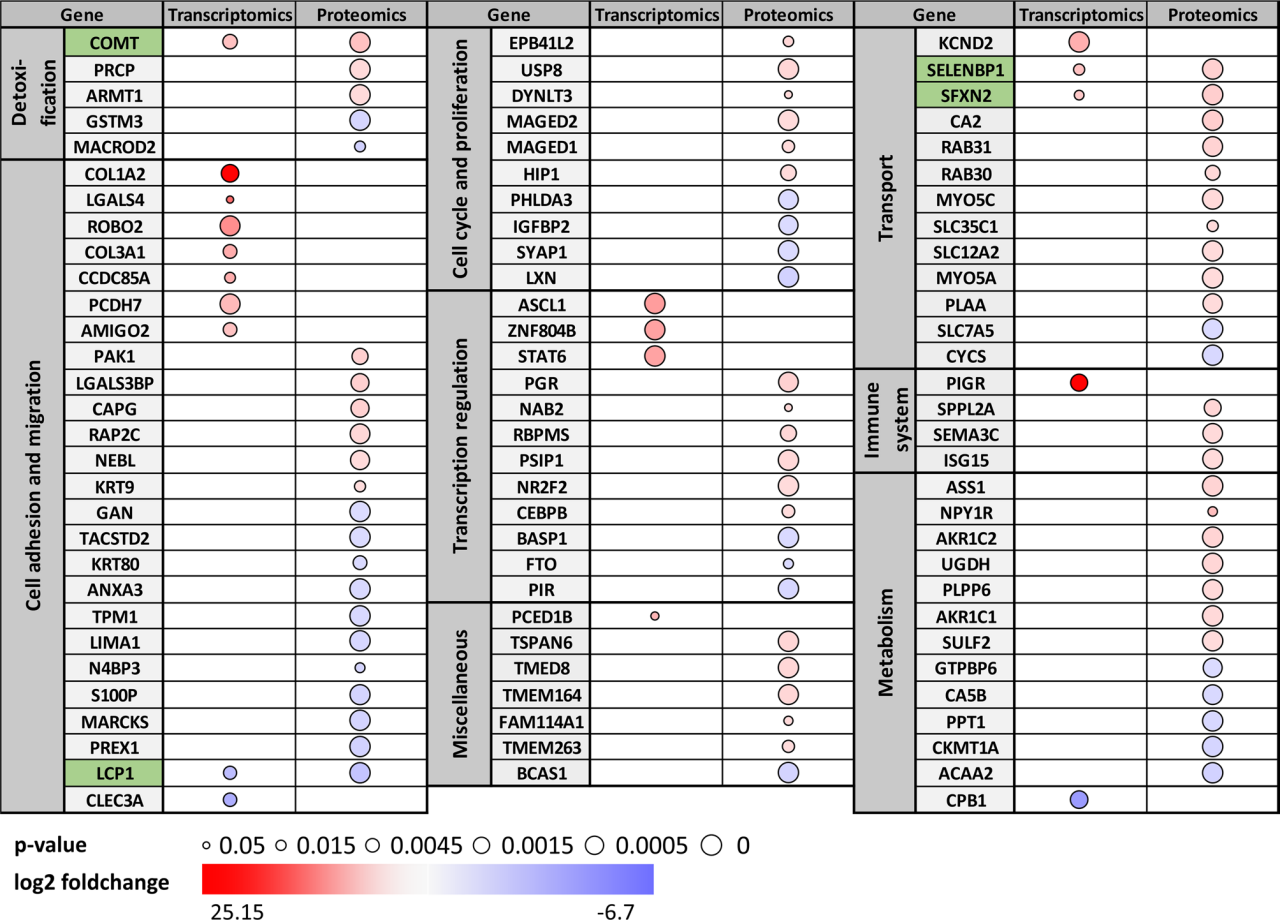
Significantly deregulated gene products in RNA-Seq (|log2FC|> 1, *p*-adj < 0.05; n = 2 per cell line) and proteomics (|log2FC|> 0.58, *q* < 0.05; n = 3 per cell line). Gene products are grouped according to their biological function. Size and color of the node represent statistical significance and log2 foldchange of the gene, respectively. Gene products significantly deregulated both in RNA-Seq and proteomics data are marked in green.

Out of 3299 genes and proteins identified in our data, 19 genes and 74 proteins were significantly deregulated in transcriptomics (|log2FC|> 1, adj. *p*-val < 0.05) and proteomics (|log2FC|> 0.58, adj. *p*-val < 0.05), respectively. COMT was the most significantly overexpressed gene in MCF7-COMT cells compared to control cells at both transcript and protein level, confirming the validity of the experiments. Other groups of deregulated gene products involved those associated with detoxification, cell adhesion and migration, cell cycle and proliferation, transcription regulation, transport, immune system and metabolism (Fig. 3).

In addition to COMT, three gene products were significantly deregulated on both transcript and protein levels. From these, sideroflexin-2 (SFXN2) is an evolutionary conserved protein involved in mitochondrial iron metabolism by regulating heme biosynthesis^21^. SFXN2 was later observed as an prognostic factor in renal, urothelial, cervical, and liver cancer and its high expression was associated with a significantly longer overall survival probability in the PANCAN cohort with more than 12,800 samples derived from 17 different tumors^22^. Furthermore, *LCP1* gene product representing plastin-2/L-plastin protein was downregulated in MCF7-COMT cells; this protein was observed to accompany tumorigenesis in malignant cells and was connected to cell migration and metastasis^23^. These genes were selected from combined omics analysis based on three biological replicates in proteomics and two biological replicates in transcriptomics and remained unchanged when additional two biological replicates were implemented into transcriptomics analysis (Supplementary Data S5). These data on the key gene products co-regulated with COMT indicate that COMT overexpression might lead to upregulation of tumor suppressing and downregulation of pro-tumorigenic gene products.

**Figure 4:**
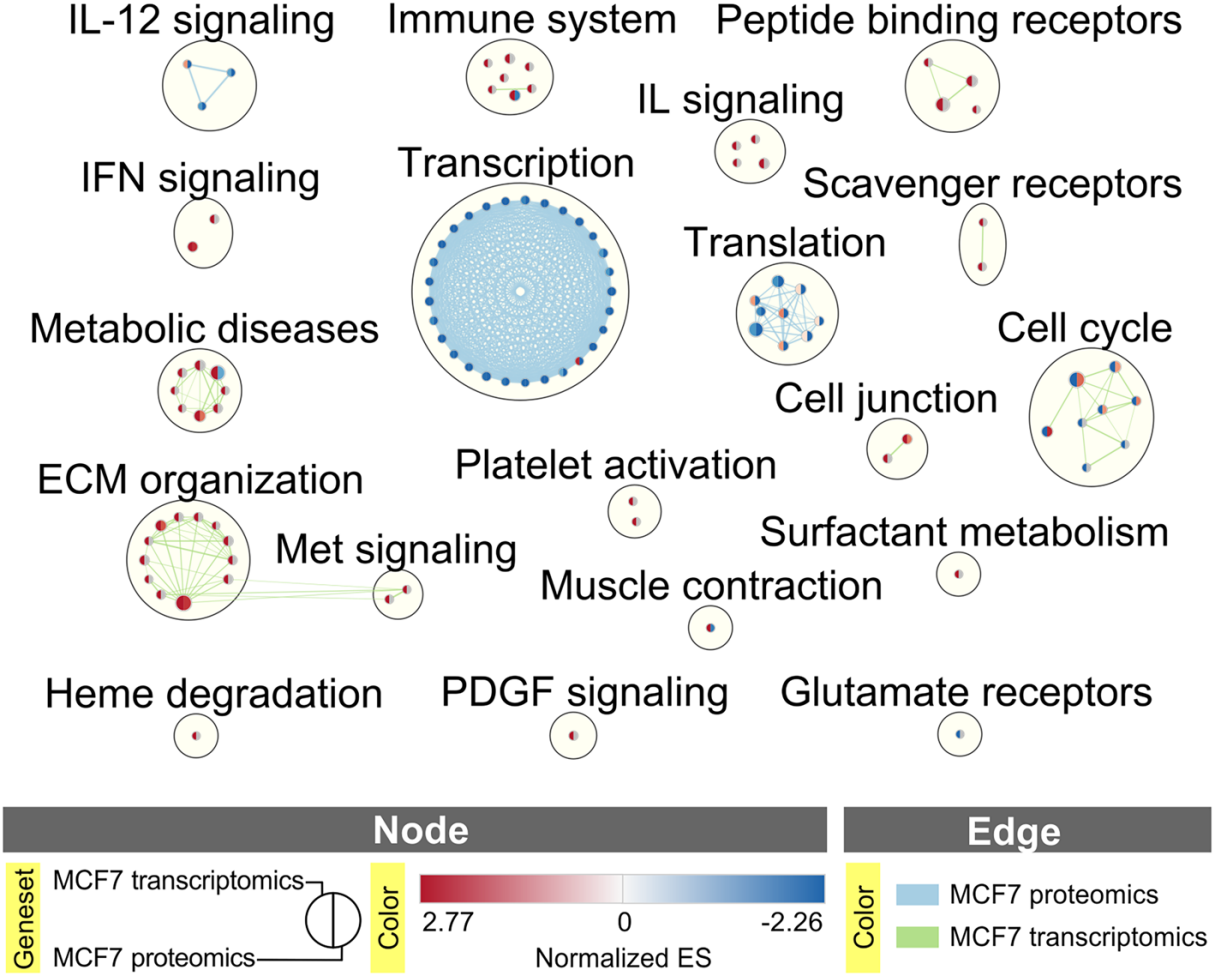
Cytoscape network of significantly enriched (FDR *q*-value < 0.05, Jacard and Overlap metric < 0.375) pathways obtained in GSEA analysis based on RNA-Seq and proteomics data.

GSEA pathway analysis of RNA-Seq and proteomics data was carried out to further recognize the changes in biological processes associated with COMT overexpression in MCF7 cells. 184 and 78 enriched pathways were identified at *p* < 0.05 (Supplementary Data S6) in RNA-Seq and proteomics datasets, respectively. The overlaps among enriched pathways on transcript and protein levels were visualized as a Cytoscape network of single biological theme clusters (Fig. 4). Largest clusters correspond to gene transcription (negative enrichment) and extracellular matrix (ECM) organization (positive enrichment in COMT overexpressing cells). The latter well supports the results of invasion assays (Figs. 1, 2) as the interaction with ECM components is a key factor of the ability of cells to invade. ECM organization cluster was directly connected to MET signaling in the visualized network (Fig. 4). This is important as MET pathway, including MET receptor and its ligand, hepatocyte growth factor (HGF), has a key role in cancer cell migration and metastasis and is an established target of breast cancer therapy. We were thus interested how the individual gene products of this pathway were regulated in response to COMT overexpression (Table 1). First, collagens alpha-2 (COL1A2) and alpha-1 (COL3A1) were the most upregulated gene products. The corresponding proteins serve as structural constituents of extracellular matrix^24^, supporting the interaction of cancer cells with ECM. On the other hand, hepatocyte growth factor activator (HGFAC) was the most downregulated protein in COMT overexpressing cells (Table 1)^25^. HGFAC, a MET ligand, is essential factor for the activation of hepatocyte growth factor (HGF)^26^. These data indicate an interplay between COMT overexpression and suppression of MET signaling.

**Table 1:** Genes and proteins involved in MET signaling deregulated in RNA-Seq and proteomics data (*p*-adj. < 0.05).

Gene	Protein name	log2FC	*p* adj. value	Dataset
COL1A2	Collagen alpha-2(I) chain	24.15	7.60E−04	Transcript
COL3A1	Collagen alpha-1(III) chain	3.24	4.11E−03	
PAK1	Serine/threonine-protein kinase PAK 1	1.25	1.60E−03	Protein
USP8	Ubiquitin carboxyl-terminal hydrolase 8	0.80	7.20E−19	
SRC	Proto-oncogene tyrosine-protein kinase Src	0.27	2.45E−02	
PTPN1	Tyrosine-protein phosphatase non-receptor type 1	0.14	2.80E−04	
ITGB1	Integrin beta-1	0.09	8.24E−03	
ITGA2	Integrin alpha-1	− 0.13	3.76E−02	
ITGA3	Integrin alpha-3	− 0.32	4.55E−02	
HGFAC	Hepatocyte growth factor activator	− 0.46	2.00E−02	

**Figure 5:**
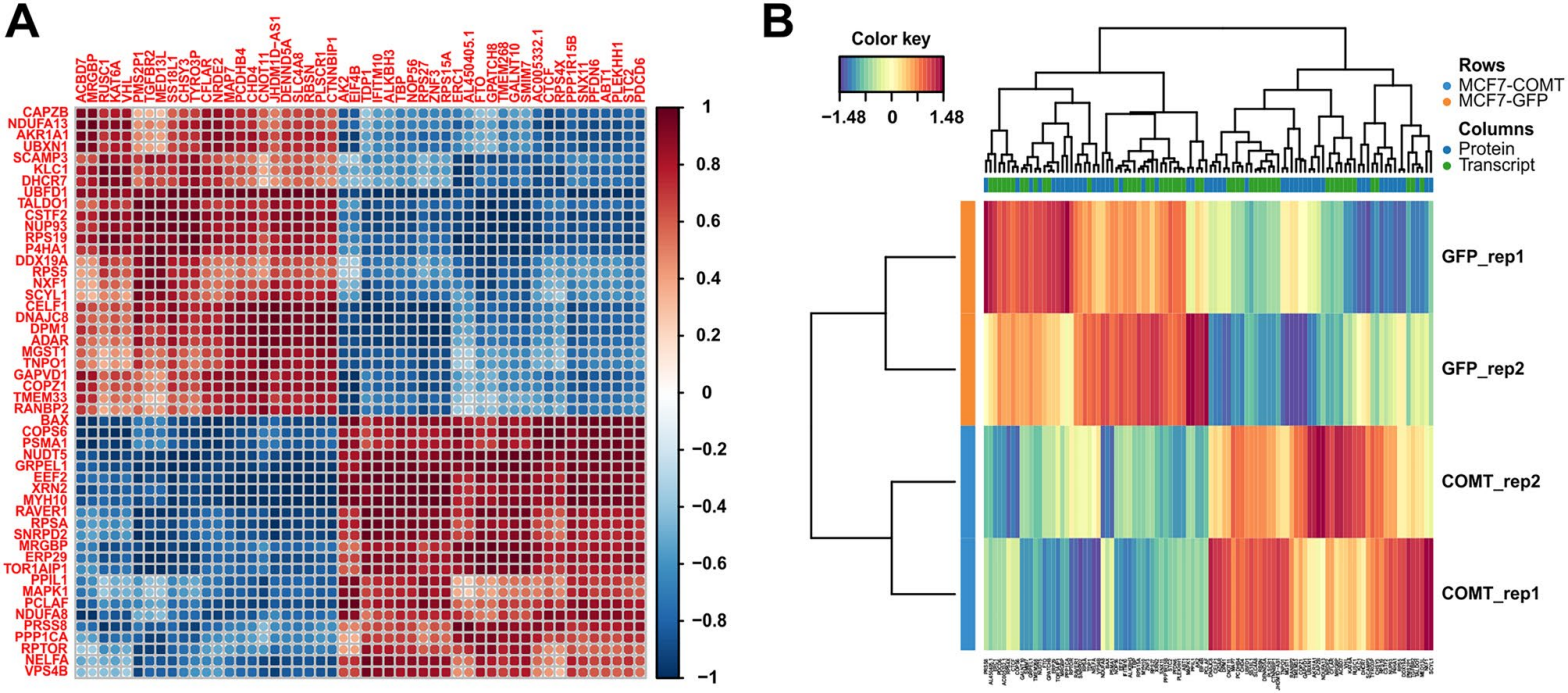
Correlation analysis (**A**) and hierarchical clustering (**B**) based on the top correlated gene products from omics analyses could distinguish the MCF7 samples with and without COMT expression.

Having big omics datasets for COMT-related network in our hands, we were interested whether these RNA-Seq and/or proteomics data can be used to simply distinguish the COMT + and COMT− cells based on their transcriptome and/or proteome profile. As single omics analysis usually does not provide enough information to give a deep understanding of a biological system, we integrated both RNA-Seq and proteomics datasets and subjected them to multivariate analysis. Correlation analysis of both omics’ datasets led to identification of top 50 transcripts and top 50 proteins that were highly correlated. Based on these, we were able to differentiate the sample subgroups in response to COMT overexpression (Fig. 5A). Accordingly, samples of MCF7-COMT and MCF7-GFP cells each clustered together based on the expression of the most correlated transcripts and proteins (Fig. 5B). Even though the integration of the data should display a broader view of samples and enrich our results with a deeper biological context, variance analysis based on the RNA-Seq and proteomics data also showed that the effect of COMT overexpression on MCF7 cells could be distinguished solely using transcriptomics, or proteomics dataset (Supplementary Fig. S6). These results confirm that both datasets are sources of a valuable and relevant information for future studies on COMT functional network.

### MET interactor SPINT2 is a key interaction partner of COMT

**Figure 6:**
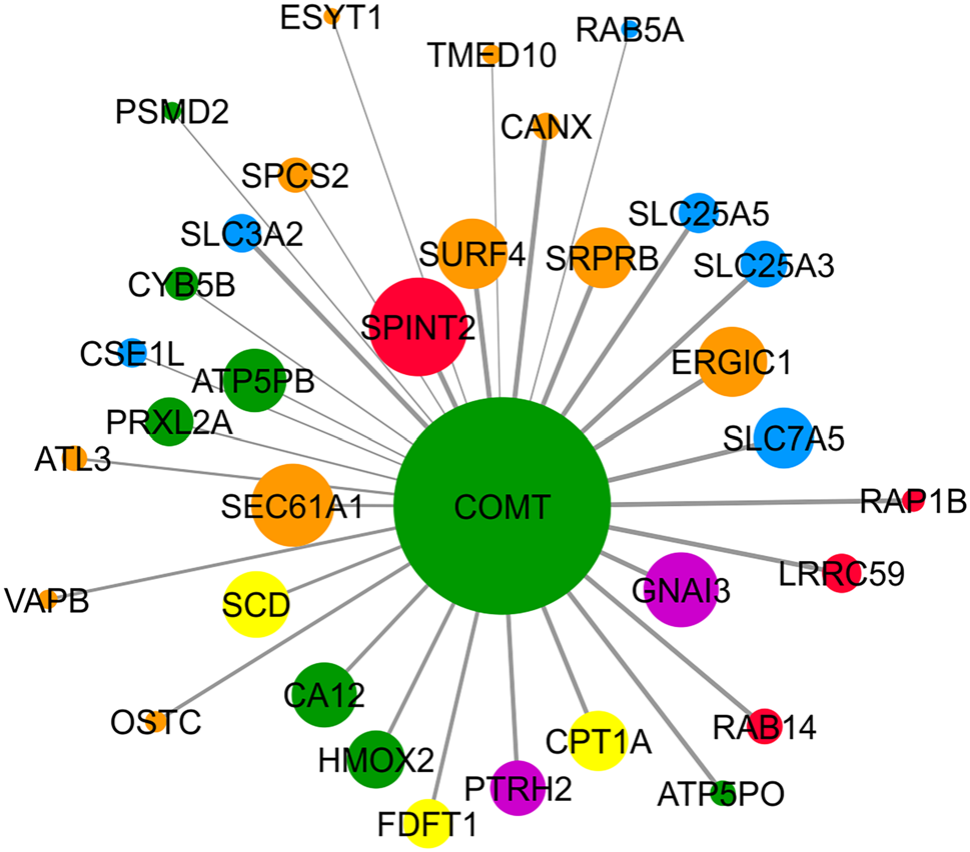
Identified COMT protein–protein interaction partners displayed by Cytoscape. Proteins involved in regulation of cell adhesion and motility, redox homeostasis, cell cycle, ER organization, protein transport and lipid metabolism according to Uniprot are marked in red, green, purple, orange, blue and yellow, respectively. Size of the nodes represent log2 of the average foldchange of COMT to control. Width of the edges represent *p*-value for the interaction.

As not only the co-expression, but also a physical interaction between proteins indicates their functional collaboration, we generated additional stably transduced cell line overexpressing COMT N-terminally tagged with streptavidin binding peptide (SBP) (MCF7-SBP-COMT) as well as the control line expressing SBP only (MCF7-SBP; see Fig. S7 for input levels of COMT and SBP into pull-down assay). Pull-down assay followed by LC-DIA-MS/MS was applied to identify protein–protein interactions of COMT. Of total 388 proteins identified and quantified (Supplementary Data S7), 48 were more abundant (log2FC > 1, *q* < 0.05) in pull-down assays in the presence of COMT, being its potential interaction partners. Subsequently, proteins more abundant (log2FC > 0, *q* < 0.05) also in MCF7-COMT vs. MCF7-GFP total cell proteome were filtered out from this list to exclude changes caused by expression and not the interaction. 32 proteins met these criteria being considered the “true” interaction partners of COMT (Fig. 6). While Ras-related proteins RAB14 and RAB5A, calnexin (CANX) and extended synaptotagmin-1 (ESYT1) have been listed as known COMT interactors in BioGRID^27^, HIPPIE^28^, MENTHA^29^ and PINA^30^ databases, the rest of the “true” interactors are new. To categorize these true interactors into REACTOME pathways, we performed GSEA that resulted in three statistically significantly positively enriched pathways (NOM *p*-value < 0.05; Table 2). As a result, the only “true” interactor that was involved in two of three identified pathways, MET RECEPTOR ACTIVATION and SIGNALING BY MST1 (Table 2) which also had the highest log2FC in pull-down assay but no significantly different abundance between MCF7-COMT vs. MCF7-GFP cells (Fig. 6), was SPINT2, a protease inhibitor known for a negative regulation of hepatocyte growth factor-induced invasion in BC cells^26^. This finding complements an information on interplay between COMT and MET signaling observed in RNA-Seq and proteomics experiments. The third pathway, SIGNALING BY NUCLEAR RECEPTORS pathway is involved in transcription regulation. Among the proteins involved in this pathway, GNAI3 was found responsible for inhibition of cell invasion and tumor progression in hepatocellular^31^ and colitis-associated carcinoma^32^, whereas SCD^33^ and CPT1A^34,35^ are involved in tumor progression and cell motility. In conclusion, pathway analysis of pull-down assay data revealed novel interesting interactors of COMT corresponding to the same functional groups as RNA-Seq and proteomics analysis (MET pathway and transcription regulation) that might cooperate in COMT activity, including its tumor suppressor role in MCF7 cells.

**Table 2:** Statistically significant enriched pathways (NOM *p* < 0.05) in the protein–protein interaction data.

Pathway	Genes involved	ES	NOM *p*-value
SIGNALING BY MST1	SPINT2	1.00	0.000
MET RECEPTOR ACTIVATION	SPINT2	1.00	0.000
SIGNALING BY NUCLEAR RECEPTORS	GNAI3, SCD, CPT1A	0.78	0.048

*ES* enrichment score, *NOM p-value* nominal *p*-value.

## Discussion

Catechol-O-methyl transferase is the enzyme required for inactivation of carcinogenic catechol estrogens, playing a tumor protective role in ER dependent cells^1–8^. In breast cancer cells, Tolba et al. showed that silencing of COMT enhanced cell proliferation of MCF7, however, the opposed was observed in cells that lack ER-α expression as MDA-MB-231^36^. Accordingly, Faktor et al. found COMT to be positively associated with migration of MDA-MB-231 cell line using SILAC proteomics^11^. Our data presented here well complement all these findings by the observation that COMT overexpression in MCF7 cells leads to decreased invasiveness of ER dependent MCF7 cells originally derived from luminal A breast tumor.

Our current study of the COMT effect on invasiveness and tumorigenesis on molecular level led to the gain of transcriptomic and proteomic profiles of MCF7-COMT and control cells. Network constructed of significantly enriched pathways based on these data (Fig. 4) showed deregulation of MET signaling pathways due to COMT overexpression. Signaling leading to interaction between hepatocyte growth factor (HGF) and MET receptor is known to contribute to metastasis development^37^. HGF is involved in tumor cell–cell interactions, matrix adhesion, migration, invasion, and angiogenesis^38^ and together with MET represent an important target for cancer therapy. We identified several deregulated proteins involved in MET signaling in the proteomic data. Serine/threonine-protein kinase PAK1 involved in MET signaling activation via inhibition of merlin^39^ and ubiquitin-specific protease 8 (USP8) regulating MET levels and stability^40,41^ were significantly overexpressed (Table 1). However, almost 1.4-fold lower expression of HGF activator (HGFAC) required for proteolytic activation of HGF inactive precursor and its conversion to heterodimer along with slight overexpression of negative regulator of MET signaling^25^, protein tyrosine phosphatase 1B (PTPN1), were observed after COMT overexpression in our data. Newly found interplay between the MET signaling and COMT expression might pose as additional element participating in inhibition of tumorigenesis mediated by COMT. Another contributing factor to the connection between COMT and MET signaling might be its novel interacting partner Kunitz-type protease inhibitor 2(SPINT2) found in our pull-down data. SPINT2 serves as inhibitor of HGFAC which is, as previously mentioned, necessary for cleaving the pro-HGF and subsequent binding of active HGF to its receptor MET^38^.

**Figure 7:**
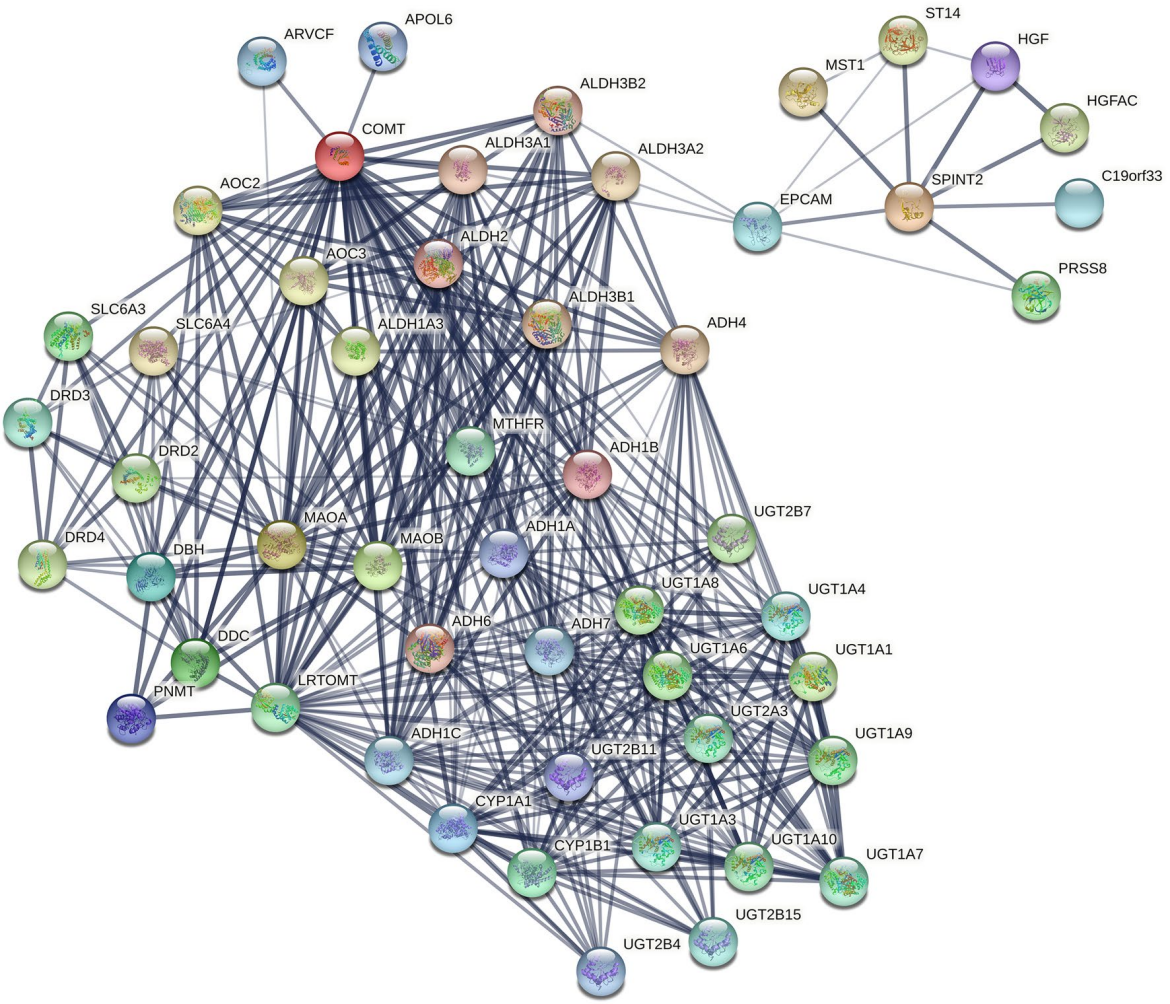
In silico analysis of protein–protein interactions between COMT and SPINT2. Analysis was conducted in STRING database using default settings except minimum required interaction score was set to medium confidence (0.400) and maximum number of interactors was set to no more than 50 interactors in 1st shell. Line thickness indicates the strength of data support.

Considering the function of SPINT2, its role as tumor suppressor was reported in several malignancies such as breast cancer^38,42^, prostate cancer^43,44^, renal cell carcinoma^45–47^, brain cancer^48–51^ and others^52,53^. SPINT2 has been found to be associated with COMT in in silico analysis so far only indirectly through epithelial cell adhesion molecule (EPCAM) and aldehyde dehydrogenase (ALDH) family using STRING database (Fig. 7) and amyloid-beta precursor protein (APP) in BioGRID database. Even though the connection between SPINT2, EPCAM and ALDH3B2 is based on their co-expression in the database, Kawaguchi et al. experimentally proved that SPINT2 stabilizes EPCAM-claudin-7 complex which prevents enhanced epithelial permeability^54^. The connection between COMT and ALDH family is based on their known joint participation in detoxification pathways^55^. Yet another identified indirect interaction between COMT and SPINT2 was mediated via APP, protein associated with occurrence of neurodegenerative diseases such as Alzheimer disease^56^. Study of these disorders led to recognition of COMT and SPINT2 as interacting partners of APP by quantitative interaction proteomics^57^ and protein array-based interactome screen^58^, respectively. Thus, the newly discovered direct interplay between these two proteins could define new mechanism of COMT tumor suppressor activity.

Moreover, the overlap of quantified data from transcriptomic and proteomic analyses showed positive enrichment of pathways involved in ECM organization in MCF7 cells overexpressing COMT. Observed changes in pathways related to collagen organization and turnover, anchoring fibril formation, and syndecan, integrin and non-integrin ECM interactions (Supplementary Data S6) might also be instrumental to demonstrate the anti-invasive effect of COMT on ER + breast cancer cells. Positive enrichment of these pathways is represented by several proteins involved in matrix turnover such as secreted (MMP-2, MMP-7, MMP-8, MMP-9, MMP-12) and membrane-type (MMP-14) matrix metalloproteinases impacting matrix integrity by degrading matrix constituents^59^, metalloproteinase inhibitor 1^60^, N-proteinases ADAMTS-2, ADAMTS-3, ADAMTS-9, ADAMTS-14 and ADAMTS-16 degrading procollagen types I, II and III^61^, cathepsin S cleaving matrix precursors intracellularly^60^, tolloid-like protein 2 proteinase cleaving the carboxy terminus of pro-collagen to release mature collagen molecules^60^, and serpins E1 and H1 serving as inhibitors of matrix turnover by inhibiting serin and cysteine proteases and controlling the collagen turnover by regulating the activity of other degrading enzymes^60^. In addition, the cluster of ECM organization related pathways is associated with collagen fibril organization and cell adhesion represented by lysyl oxidase family member LOXL1 stabilizing the mature collagen fibrils and elastin^60^, lysyl hydroxylase family member PLOD2 involved in crosslinking the collagen fibrils^62^, and several matrix constituents^60^. These include proteoglycan versican, glycoproteins tenascin C, laminin subunit beta-3 forming basal lamina^60^, intracellular adhesion molecule 4 regulating cell adhesion and assembly of other matrix molecules^63^, and sialo-glycoprotein vascular cell adhesion molecule 1 serving pivotal role in the adhesion of cells to the vascular endothelium^64^. Deregulation of ECM organization in our data might conduce the anti-invasive impact of COMT on ER + BC cells.

According to the results of pathway analysis based on transcriptomics and proteomics, the most negatively enriched pathways are associated with transcription regulation (Supplementary Data S6). Proteins contributing to the enrichment of these pathways are important regulators of the cell cycle, particularly cyclin-dependent kinase 1 initiating the mitotic progression^65^, CDKN2A also known as p16 acting as tumor suppressor through inhibition of cell cycle^66^, and cell cycle marker proliferating cell nuclear antigen (PCNA) also involved in DNA replication and repair^67^. These changes in transcriptomic and proteomic profiles after COMT overexpression indicate the possible mechanism behind decreased invasiveness due to upregulation of COMT in MCF7 cells arising from the molecular changes in ECM organization and down-regulation of cell cycle.

Efforts to understand the tumor suppressor role of COMT in cancer cells in hormone receptor positive tumors were previously recorded: Estrogen (17β-estradiol) decreases the mRNA levels of COMT in time- and dose-dependent manner mediated by estrogen responsive elements in its proximal promoter and CCAAT/enhancer binding sites in its distal promoter^68,69^. Subsequently, the epigenetic mechanism of estrogen-induced COMT downregulation by CpG site-specific methylation within its distal promoter was also determined^70^. The negative effect of estrogen on COMT expression and its impact on the inhibition of tumorigenesis was observed on ER + breast cancer cell line^36^, as well as reduced expression of COMT in ER + breast tumor samples^11^. Based on such evidence, beneficial effects of anti-estrogen therapy (e.g. with tamoxifen) in ER + BC patients may include also increased protein levels and activity of COMT, however, this assumption requires further confirmation and evaluation, also because the knowledge on ER binding to COMT promoter remains unclear^71–73^. In our previous study^11^ we found association of increased COMT protein levels with lymph node metastasis of triple negative breast tumors. COMT may thus hypothetically represent a potential therapeutic target for triple negative breast cancer. In the present study we describe the tumor-suppressor role of COMT in luminal A breast cancer, these all in accordance with findings by Tolba et al*.* at cellular level^34^. From the translational perspective, these findings highlight the need of personalized therapy of BC patients.

As of the limitations of the study, our results are based on a single cellular model of luminal A BC subtype overexpressing COMT in hormonally independent expression system. The future experiments should validate our conclusions in other cell lines in vitro, or as cell line derived xenograft mice models in vivo. An impact of hormones and hormonal receptors on COMT expression and function in BC cells also requires more detailed analyses. Last but not least, our results open an opportunity for interesting validation and more detailed functional studies on the COMT-SPINT2 crosstalk and its role in breast cancer phenotype.

In conclusion, cell invasion assays, RNA-Seq and proteomics data resulted in identification of anti-tumorigenic role of COMT represented by decreased cell invasion, positive and negative enrichment of pathways involved in ECM organization and regulation of transcription, respectively. COMT might act as an anti-invasive agent on ER + breast cancer also through its newly discovered interplay with MET signaling via its interacting partner, SPINT2, leading to the inhibition of this pro-tumorigenic pathway. Therefore, we confirm the predicted tumor suppressor role of COMT in luminal breast cancer in spite of opposite recognized effect of COMT in estrogen-independent MDA-MB-231 line associated with cell migration and metastasis in our previous study^11^.

## Supplementary Information

Supplementary Information 1.

Supplementary Information 2.

Supplementary Information 3.

Supplementary Information 4.

Supplementary Information 5.

Supplementary Information 6.

Supplementary Information 7.

Supplementary Information 8.

## Data Availability

The raw mass spectrometry proteomics data and output files for the total proteome and pull-down analyses have been deposited in the ProteomeXchange Consortium via the Proteomics Identifications (PRIDE) partner repository (http://www.ebi.ac.uk/pride/archive/) with the dataset identifier PXD033833. The raw and processed RNA-Seq data have been deposited in NCBI's Gene Expression Omnibus^74^ and are accessible through GEO Series accession number GSE203435 (https://www.ncbi.nlm.nih.gov/geo/query/acc.cgi?acc=GSE203435).
