# Supplementary material for: Catechol-O-methyl transferase suppresses cell invasion and interplays with MET signaling in estrogen dependent breast cancer
Source: Sci Rep. 2023 Jan 23;13:1285. doi: 10.1038/s41598-023-28078-1 (PMC9870911; doi:10.1038/s41598-023-28078-1)
Supplement: Supplementary file 1 — Supplementary Information 1. [file 41598_2023_28078_MOESM1_ESM.zip › Supplementary Data S1/Imaris original images/Supplementary Data S1 _Legend.docx]

**Supplementary Data S1. Full-size, full-resolution Imaris images of the MCF7 3D spheroids.** File includes 5 directories named day 0 (T=0h) – day 4 (T=96h). Each directory contains 3 images of spheroids grown from MCF7-COMT cells and 3 images of spheroids grown from MCF7-GFP cells. The images correspond to the Fig. 2 and S4.
