# Supplementary figures and images for: Catechol-O-methyl transferase suppresses cell invasion and interplays with MET signaling in estrogen dependent breast cancer
Source: Sci Rep. 2023 Jan 23;13:1285. doi: 10.1038/s41598-023-28078-1 (PMC9870911; doi:10.1038/s41598-023-28078-1)

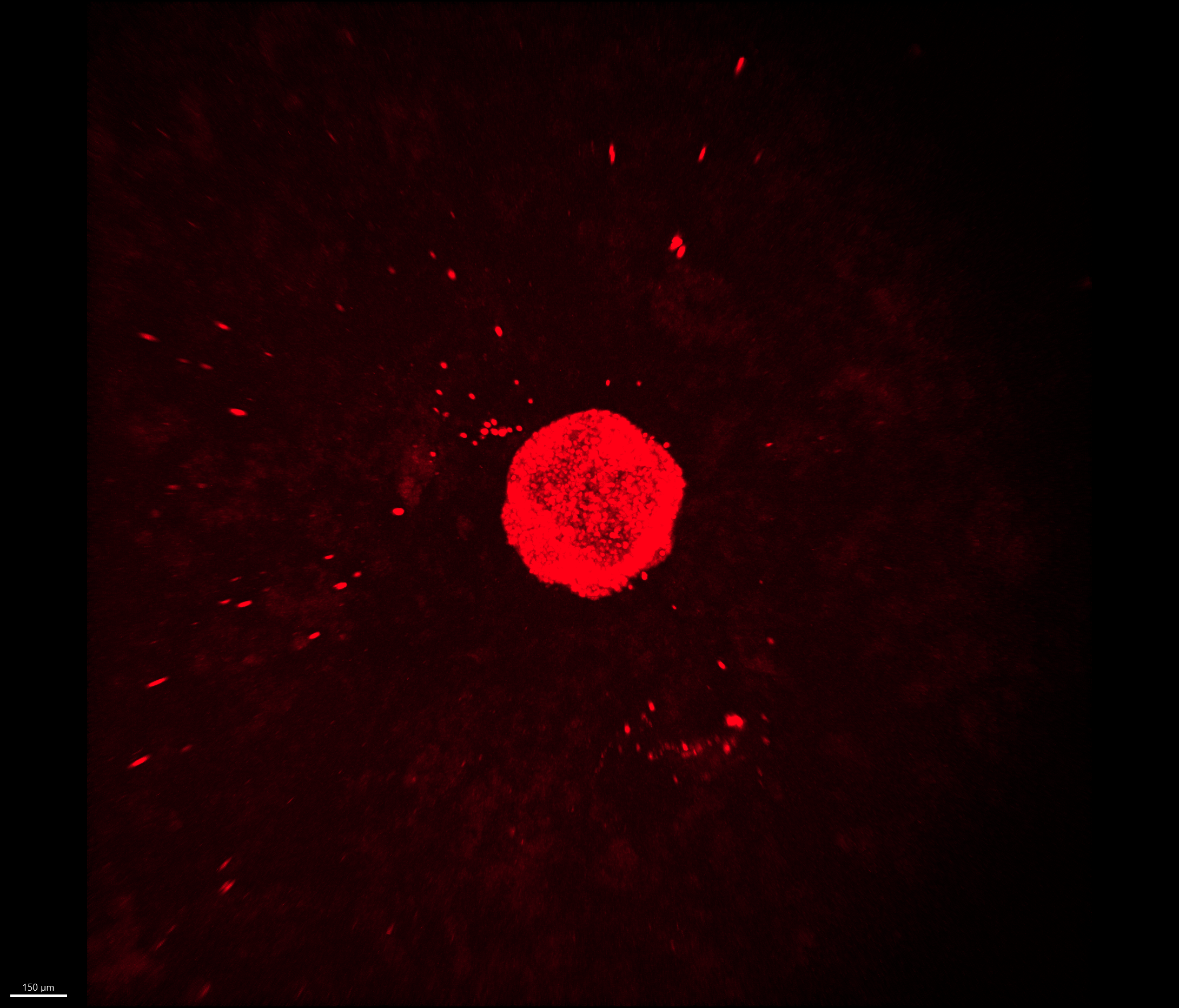

Supplement: Supplementary file 1 — Supplementary Information 1. [file 41598_2023_28078_MOESM1_ESM.zip › Supplementary Data S1/Imaris original images/day 0 (T=0h)/MCF7 COMT 1_[ims1_2021-05-17T11-13-55.365]_2021-05-17T11-23-32.121.tif]

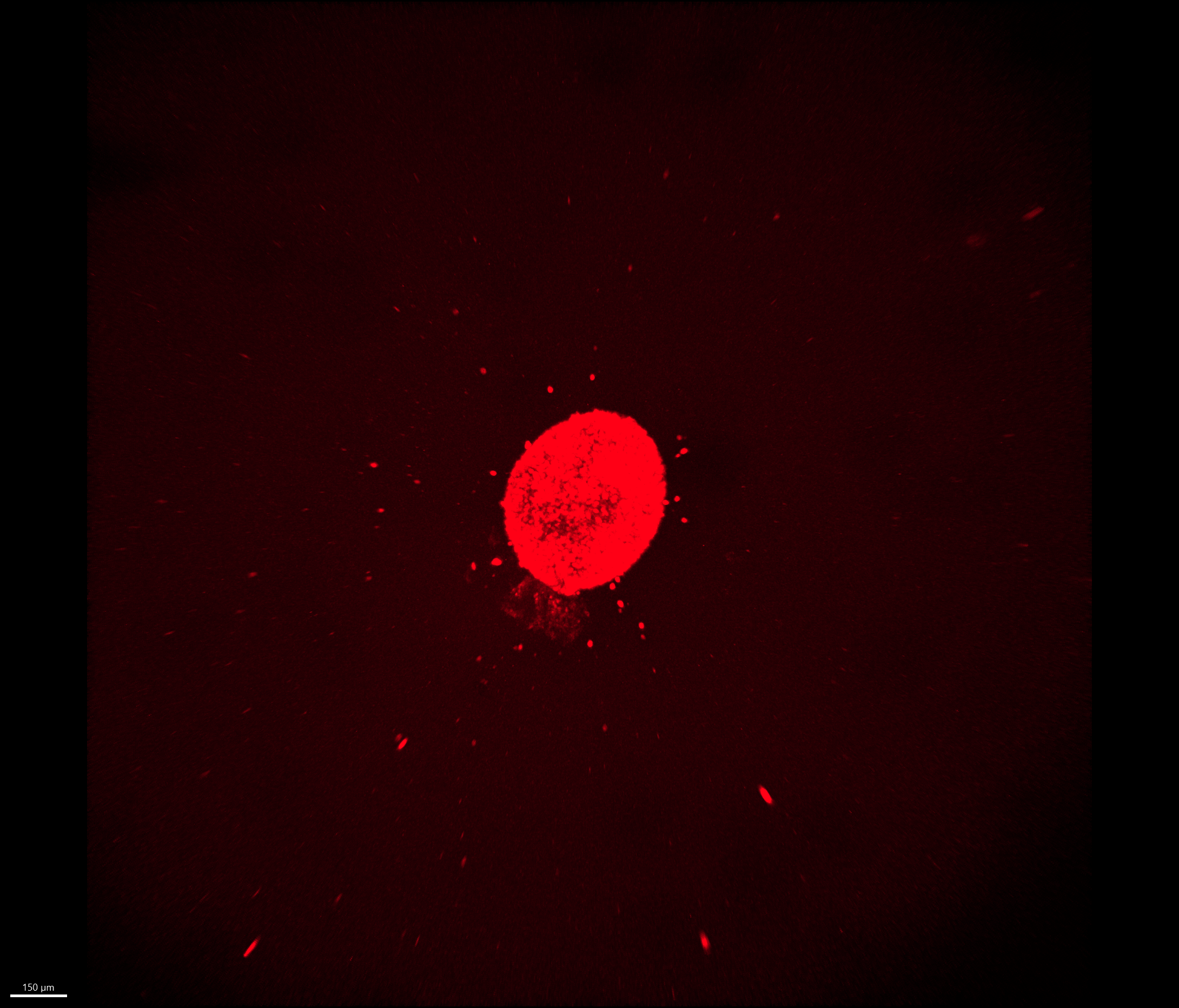

Supplement: Supplementary file 1 — Supplementary Information 1. [file 41598_2023_28078_MOESM1_ESM.zip › Supplementary Data S1/Imaris original images/day 0 (T=0h)/MCF7 COMT 2_[ims1_2021-05-17T11-13-55.365]_2021-05-17T11-24-09.919.tif]

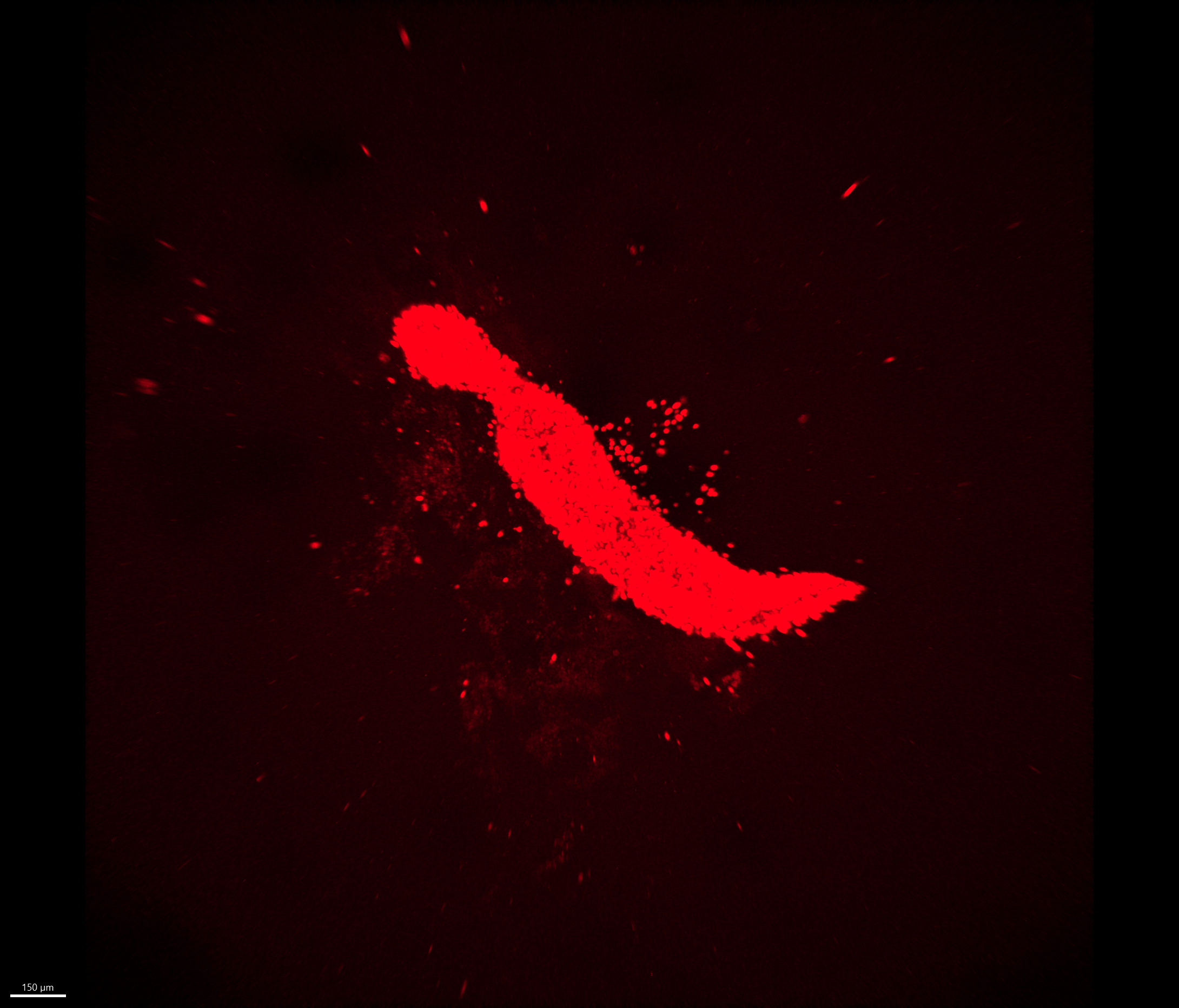

Supplement: Supplementary file 1 — Supplementary Information 1. [file 41598_2023_28078_MOESM1_ESM.zip › Supplementary Data S1/Imaris original images/day 0 (T=0h)/MCF7 COMT 3_[ims1_2021-05-17T11-13-55.365]_2021-05-17T11-24-41.447.tif]

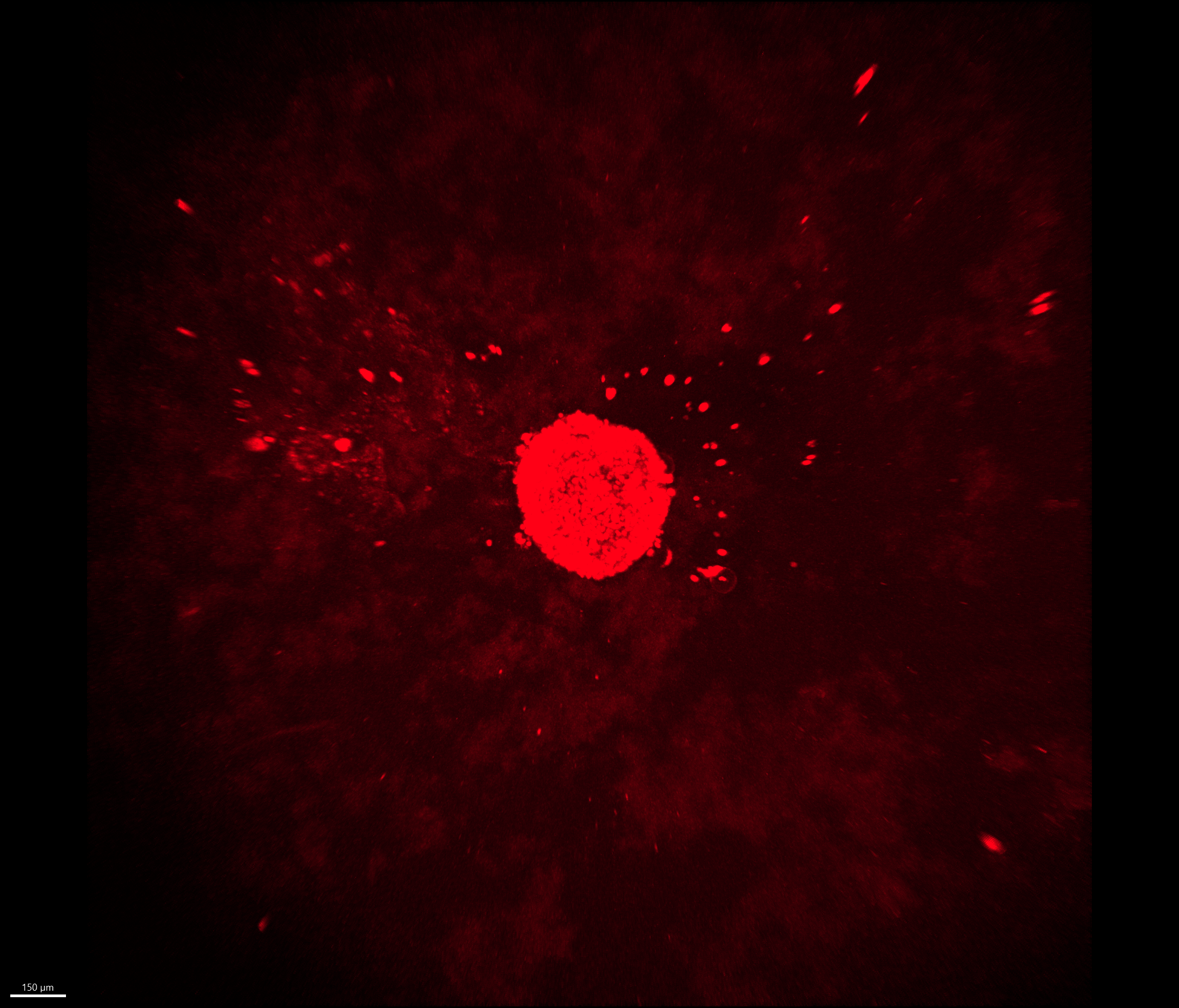

Supplement: Supplementary file 1 — Supplementary Information 1. [file 41598_2023_28078_MOESM1_ESM.zip › Supplementary Data S1/Imaris original images/day 0 (T=0h)/MCF7 GFP 1_[ims1_2021-05-17T11-13-55.365]_2021-05-17T11-25-10.187.tif]

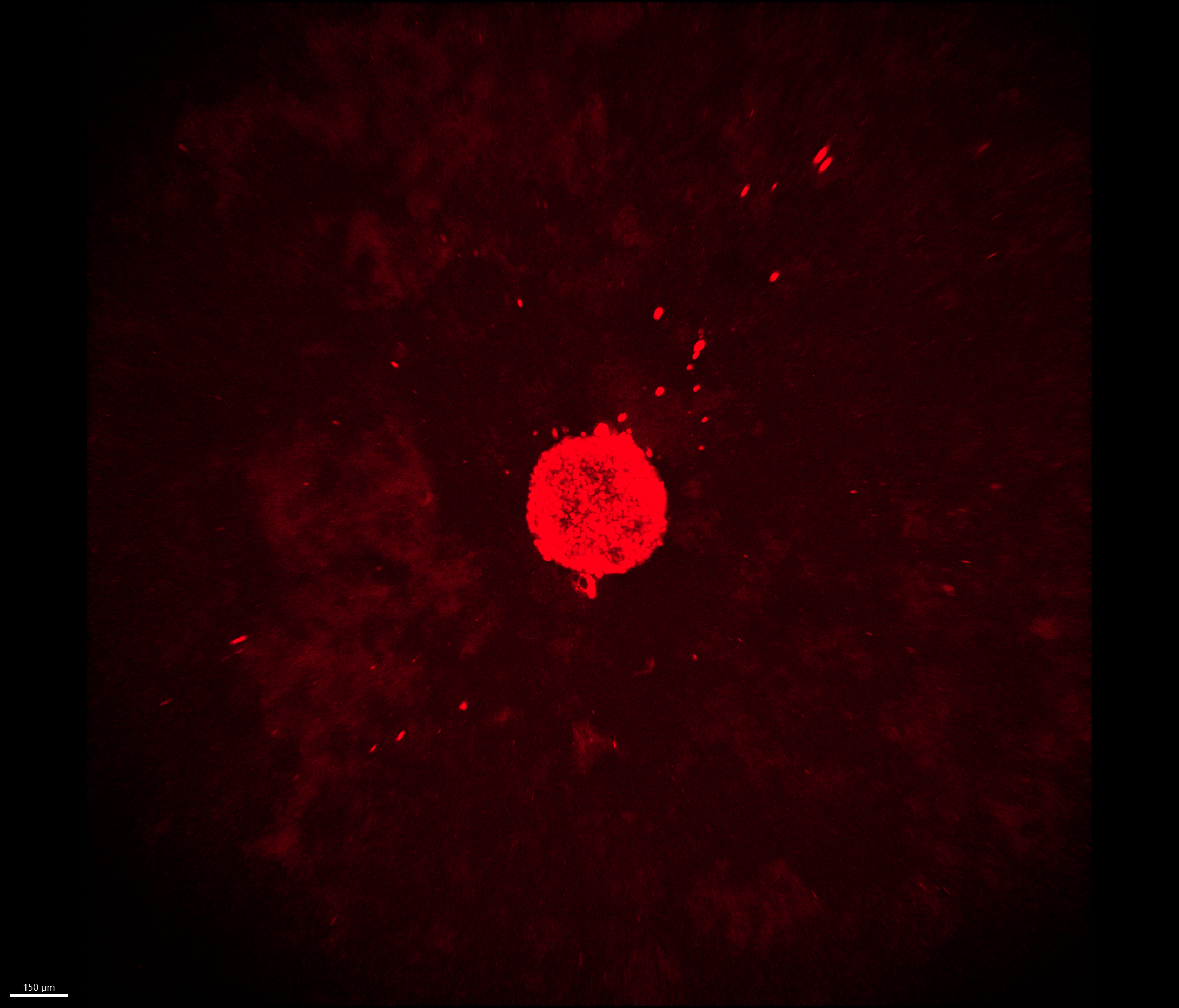

Supplement: Supplementary file 1 — Supplementary Information 1. [file 41598_2023_28078_MOESM1_ESM.zip › Supplementary Data S1/Imaris original images/day 0 (T=0h)/MCF7 GFP 2_[ims1_2021-05-17T11-13-55.365]_2021-05-17T11-25-35.702.tif]

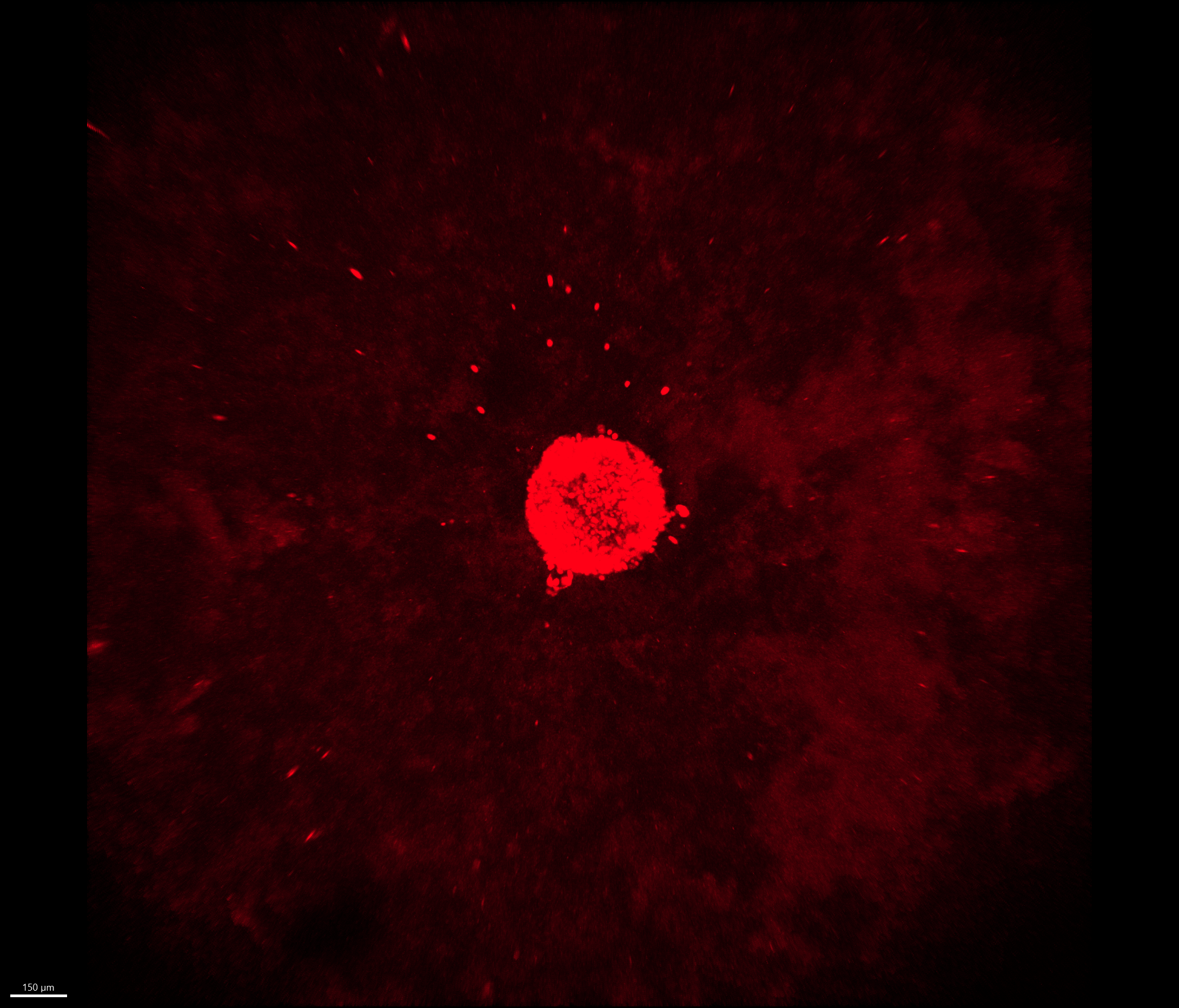

Supplement: Supplementary file 1 — Supplementary Information 1. [file 41598_2023_28078_MOESM1_ESM.zip › Supplementary Data S1/Imaris original images/day 0 (T=0h)/MCF7 GFP 3_[ims1_2021-05-17T11-13-55.365]_2021-05-17T11-26-04.582.tif]

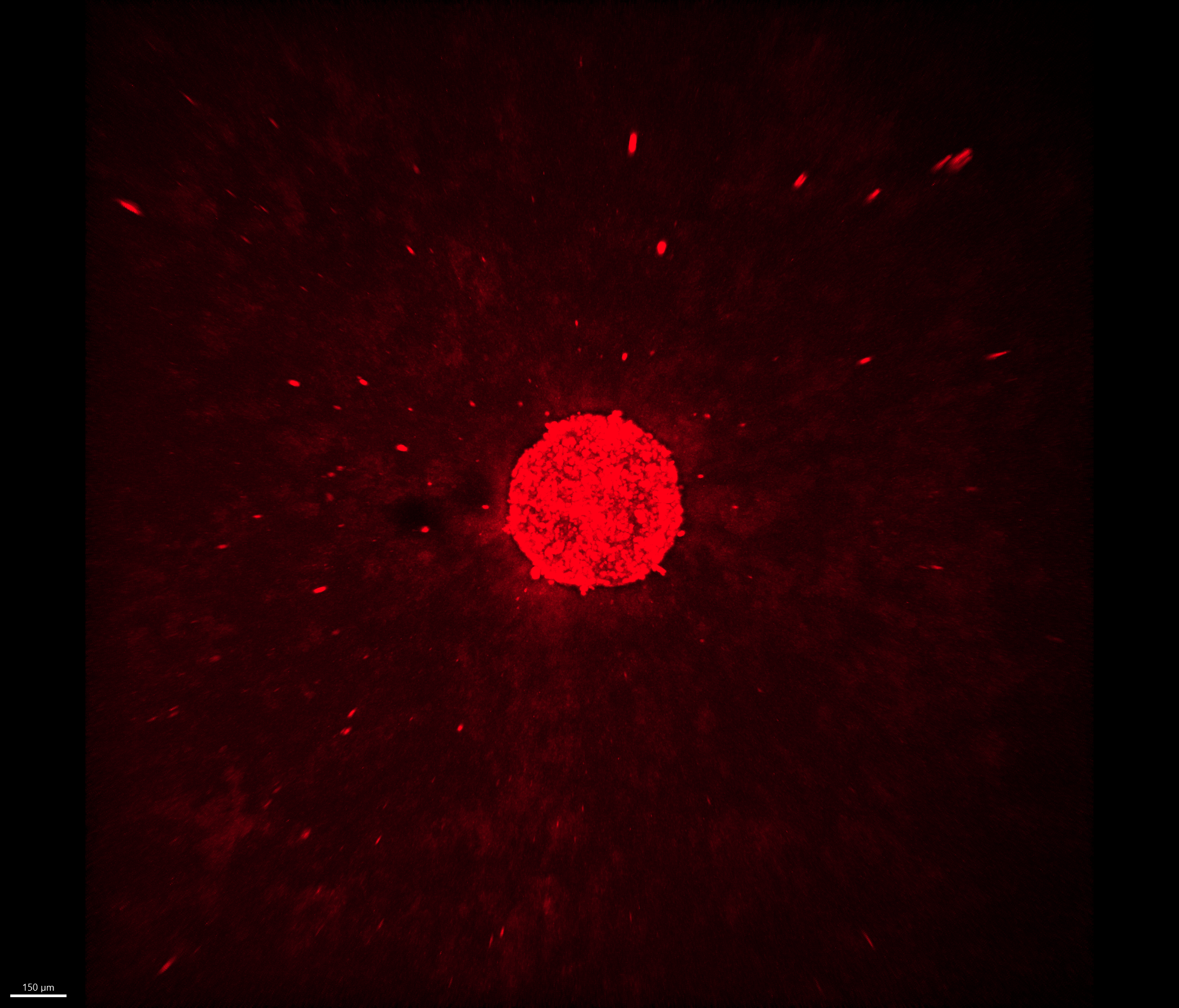

Supplement: Supplementary file 1 — Supplementary Information 1. [file 41598_2023_28078_MOESM1_ESM.zip › Supplementary Data S1/Imaris original images/day 1 (T=24h)/MCF7 COMT 1_[ims1_2021-05-17T11-16-25.943]_2021-05-17T11-26-43.533.tif]

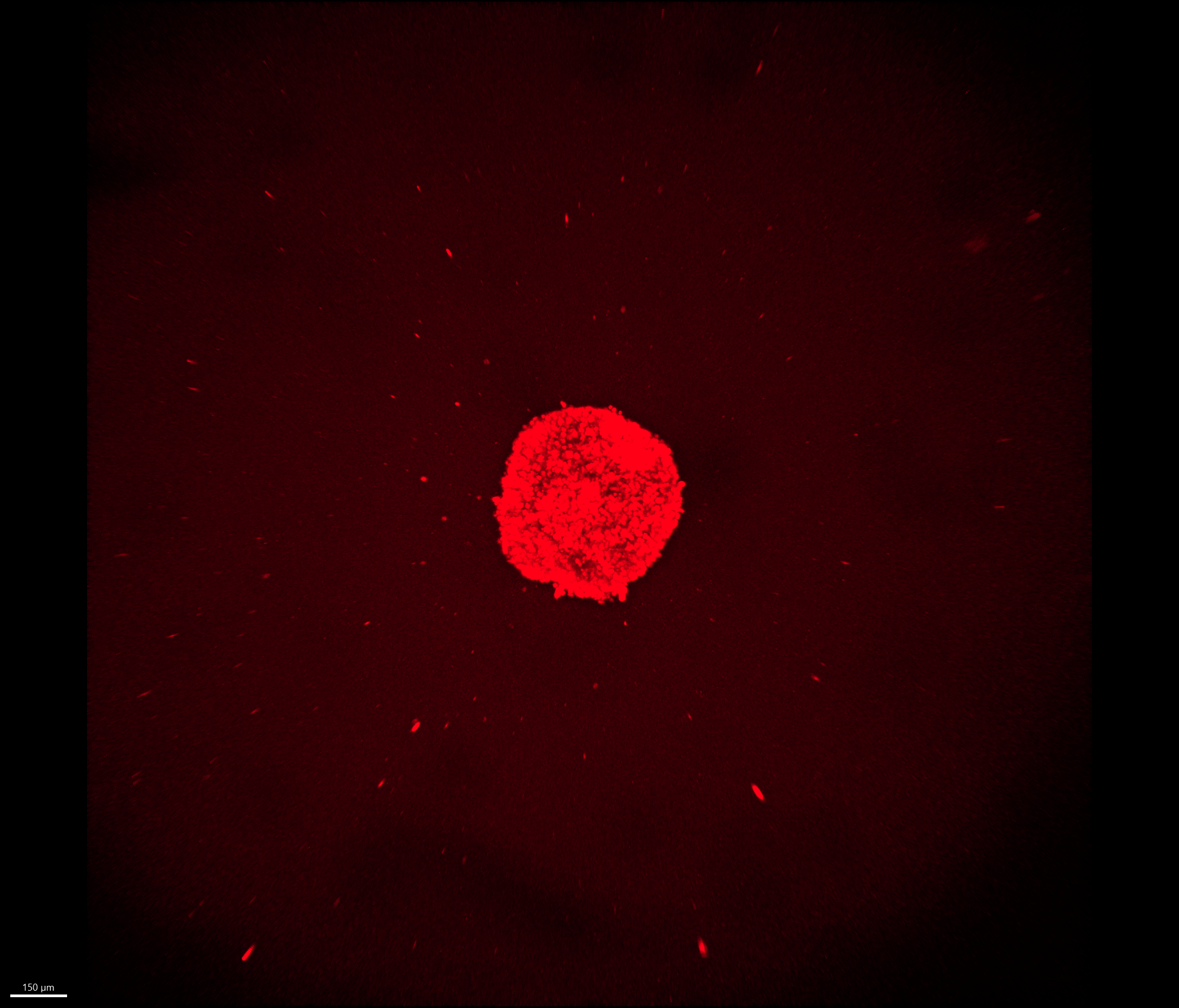

Supplement: Supplementary file 1 — Supplementary Information 1. [file 41598_2023_28078_MOESM1_ESM.zip › Supplementary Data S1/Imaris original images/day 1 (T=24h)/MCF7 COMT 2_[ims1_2021-05-17T11-16-25.943]_2021-05-17T11-27-14.753.tif]

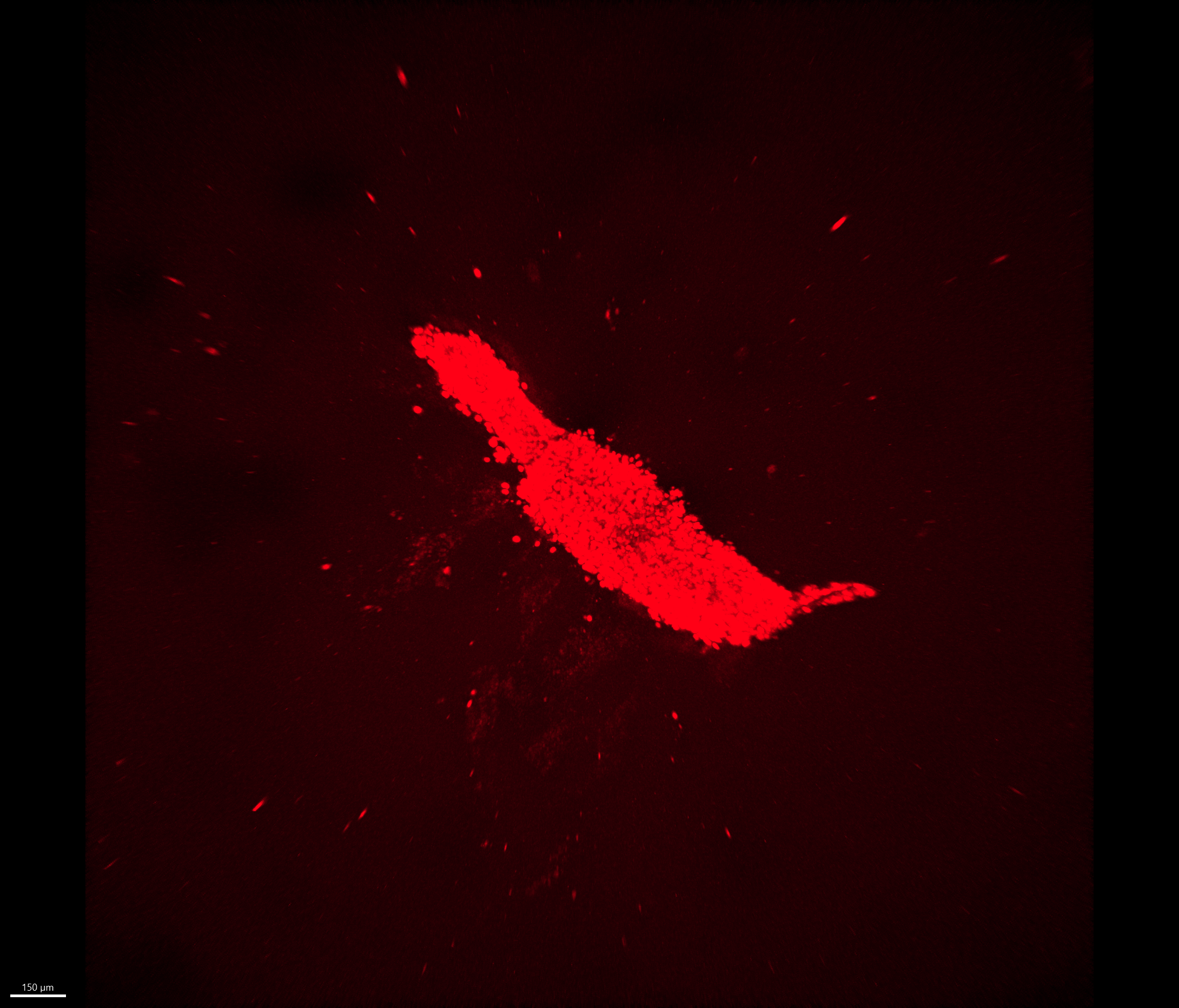

Supplement: Supplementary file 1 — Supplementary Information 1. [file 41598_2023_28078_MOESM1_ESM.zip › Supplementary Data S1/Imaris original images/day 1 (T=24h)/MCF7 COMT 3_[ims1_2021-05-17T11-16-25.943]_2021-05-17T11-27-40.818.tif]

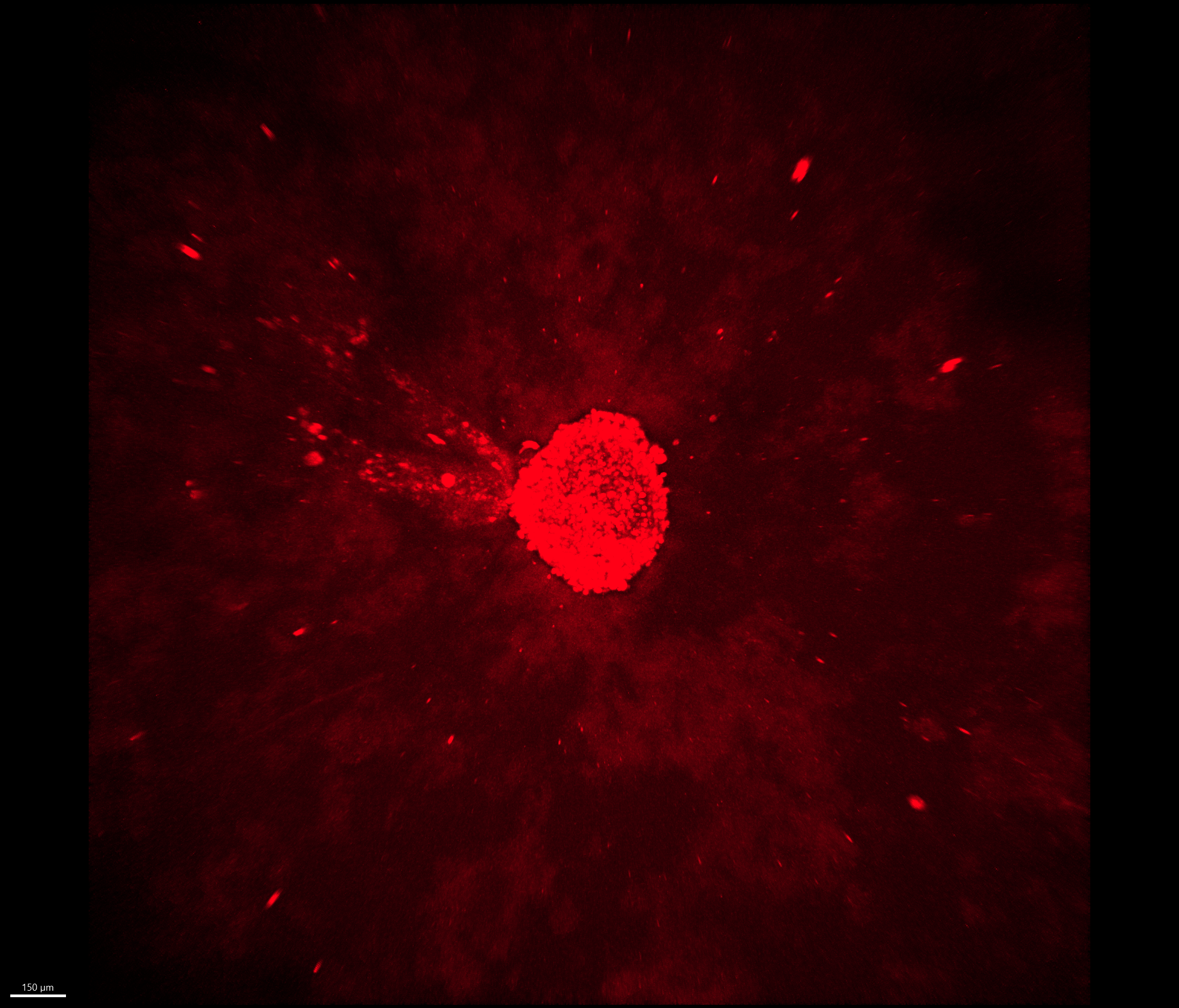

Supplement: Supplementary file 1 — Supplementary Information 1. [file 41598_2023_28078_MOESM1_ESM.zip › Supplementary Data S1/Imaris original images/day 1 (T=24h)/MCF7 GFP 1_[ims1_2021-05-17T11-16-25.943]_2021-05-17T11-28-11.747.tif]

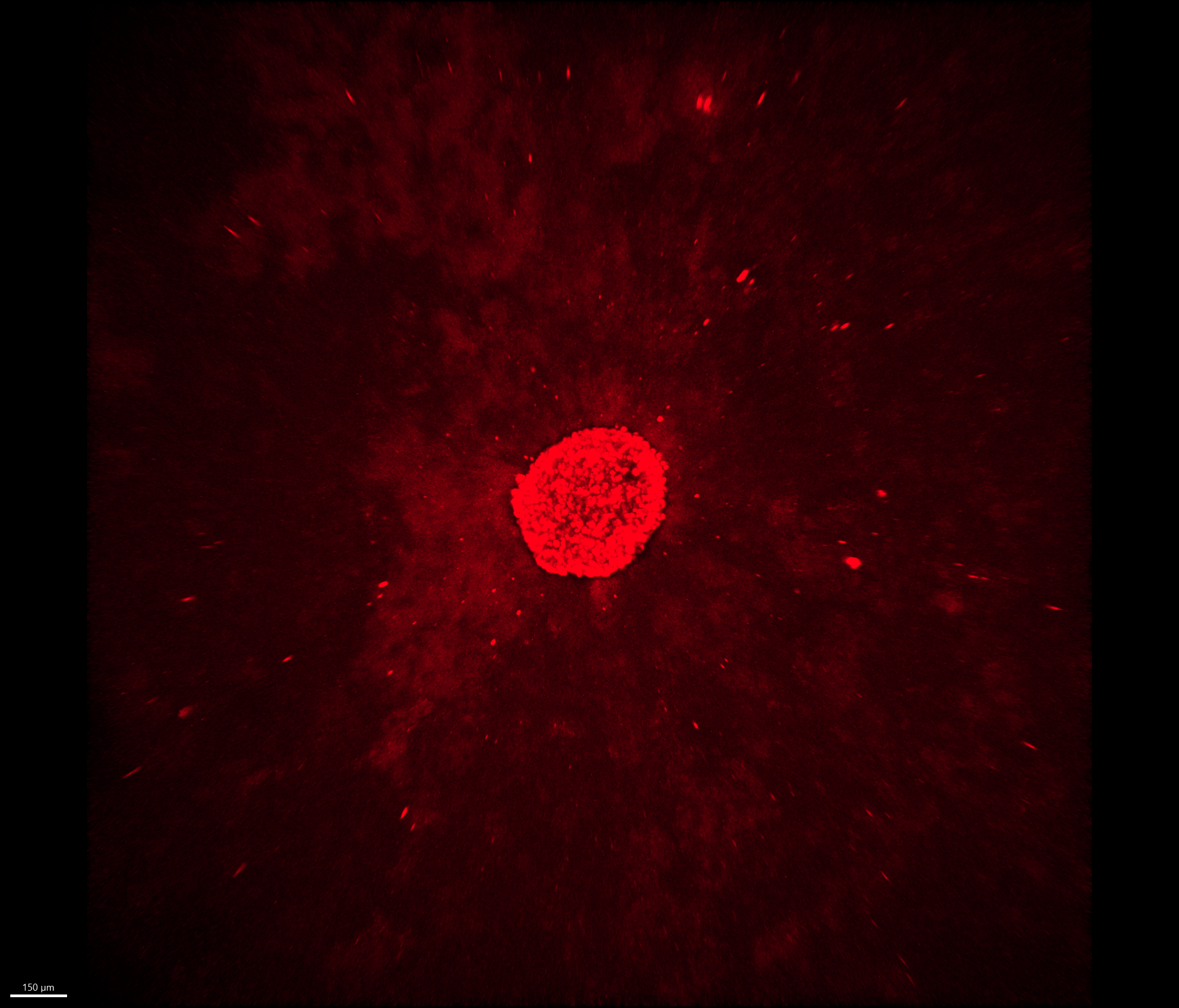

Supplement: Supplementary file 1 — Supplementary Information 1. [file 41598_2023_28078_MOESM1_ESM.zip › Supplementary Data S1/Imaris original images/day 1 (T=24h)/MCF7 GFP 2_[ims1_2021-05-17T11-16-25.943]_2021-05-17T11-29-02.743.tif]

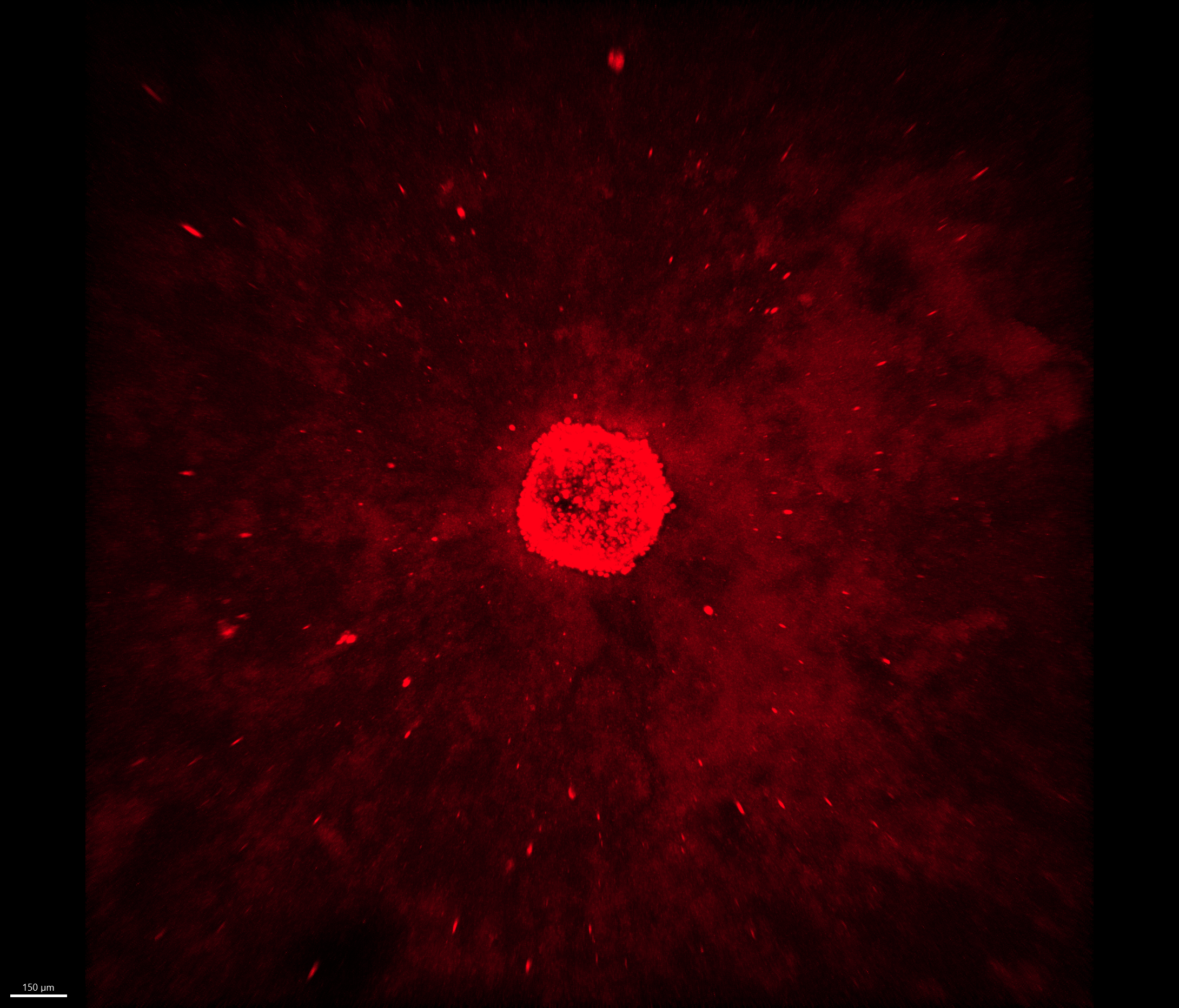

Supplement: Supplementary file 1 — Supplementary Information 1. [file 41598_2023_28078_MOESM1_ESM.zip › Supplementary Data S1/Imaris original images/day 1 (T=24h)/MCF7 GFP 3_[ims1_2021-05-17T11-16-25.943]_2021-05-17T11-29-35.696.tif]

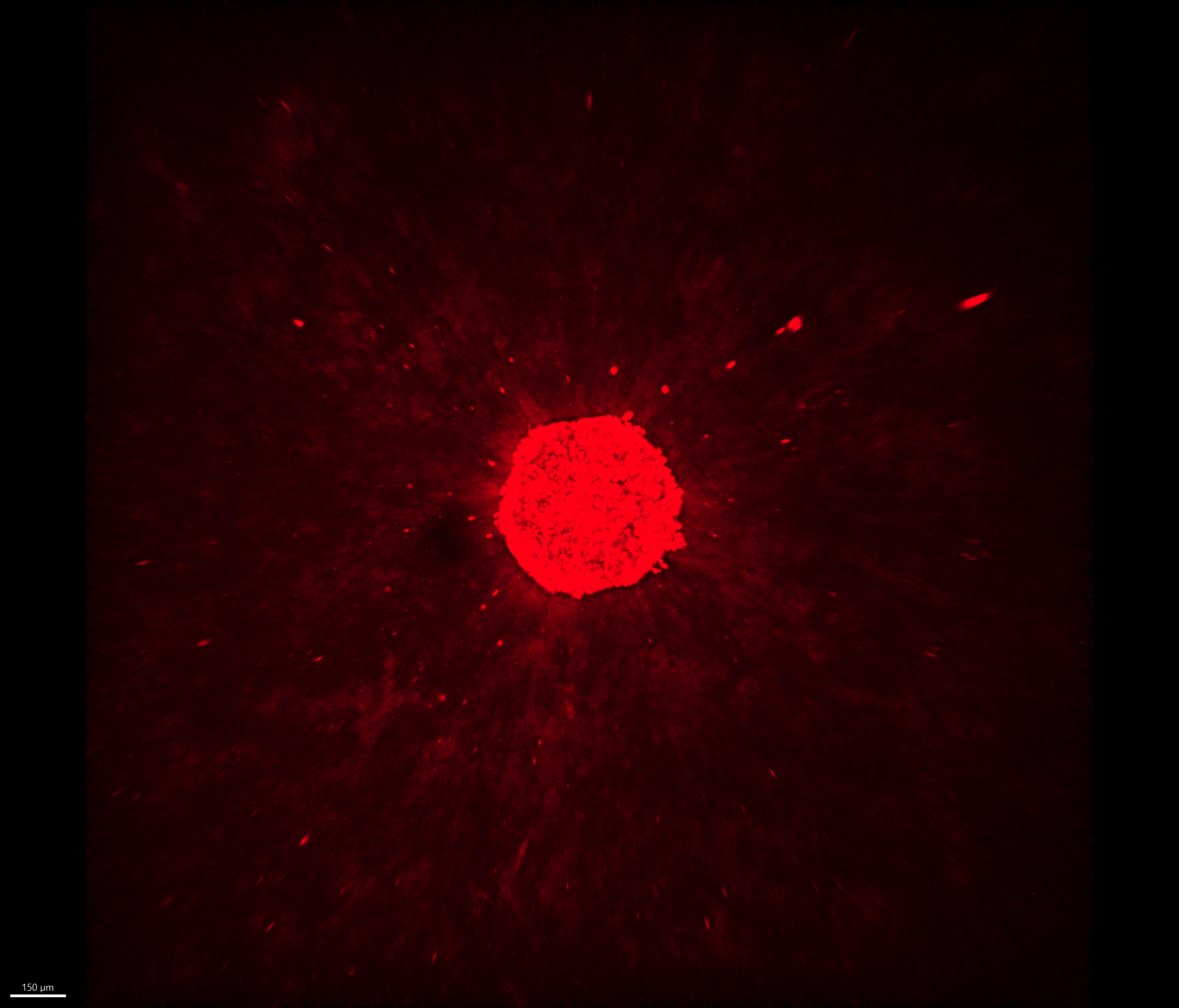

Supplement: Supplementary file 1 — Supplementary Information 1. [file 41598_2023_28078_MOESM1_ESM.zip › Supplementary Data S1/Imaris original images/day 2 (T=48h)/MCF7 COMT 1_[ims1_2021-05-17T11-18-07.782]_2021-05-17T11-31-00.066.tif]

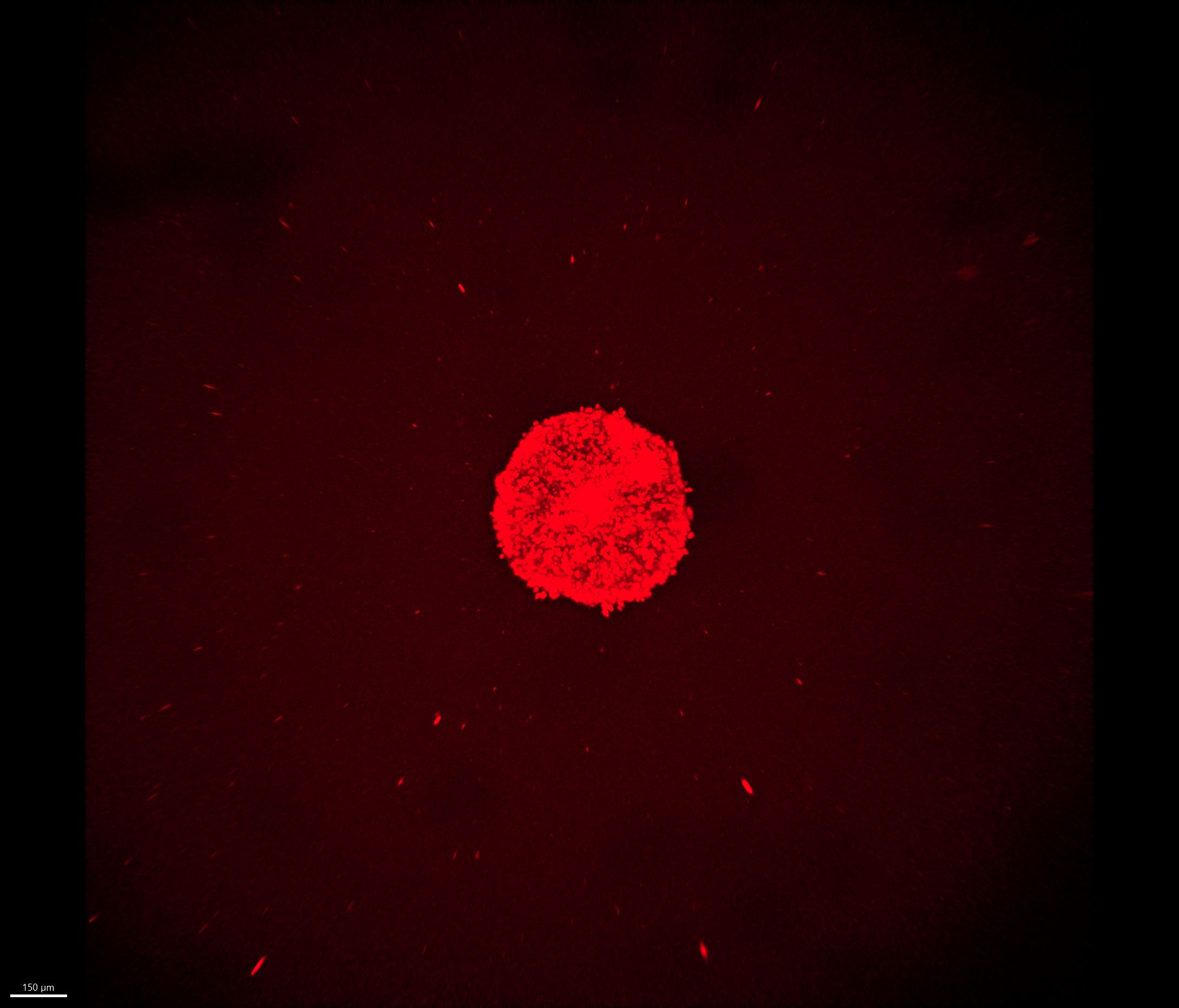

Supplement: Supplementary file 1 — Supplementary Information 1. [file 41598_2023_28078_MOESM1_ESM.zip › Supplementary Data S1/Imaris original images/day 2 (T=48h)/MCF7 COMT 2_[ims1_2021-05-17T11-18-07.782]_2021-05-17T11-31-26.515.tif]

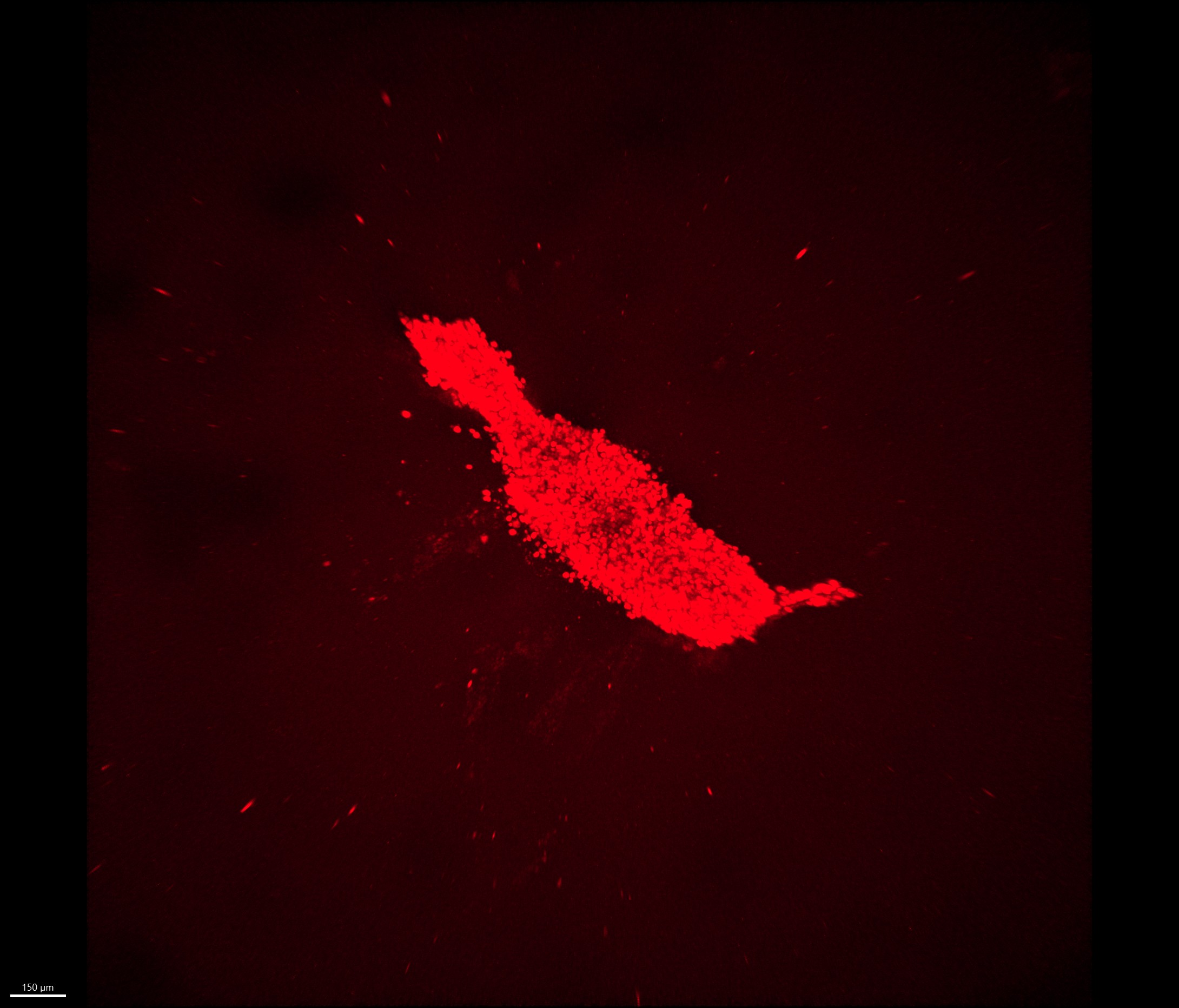

Supplement: Supplementary file 1 — Supplementary Information 1. [file 41598_2023_28078_MOESM1_ESM.zip › Supplementary Data S1/Imaris original images/day 2 (T=48h)/MCF7 COMT 3_[ims1_2021-05-17T11-18-07.782]_2021-05-17T11-31-55.665.tif]

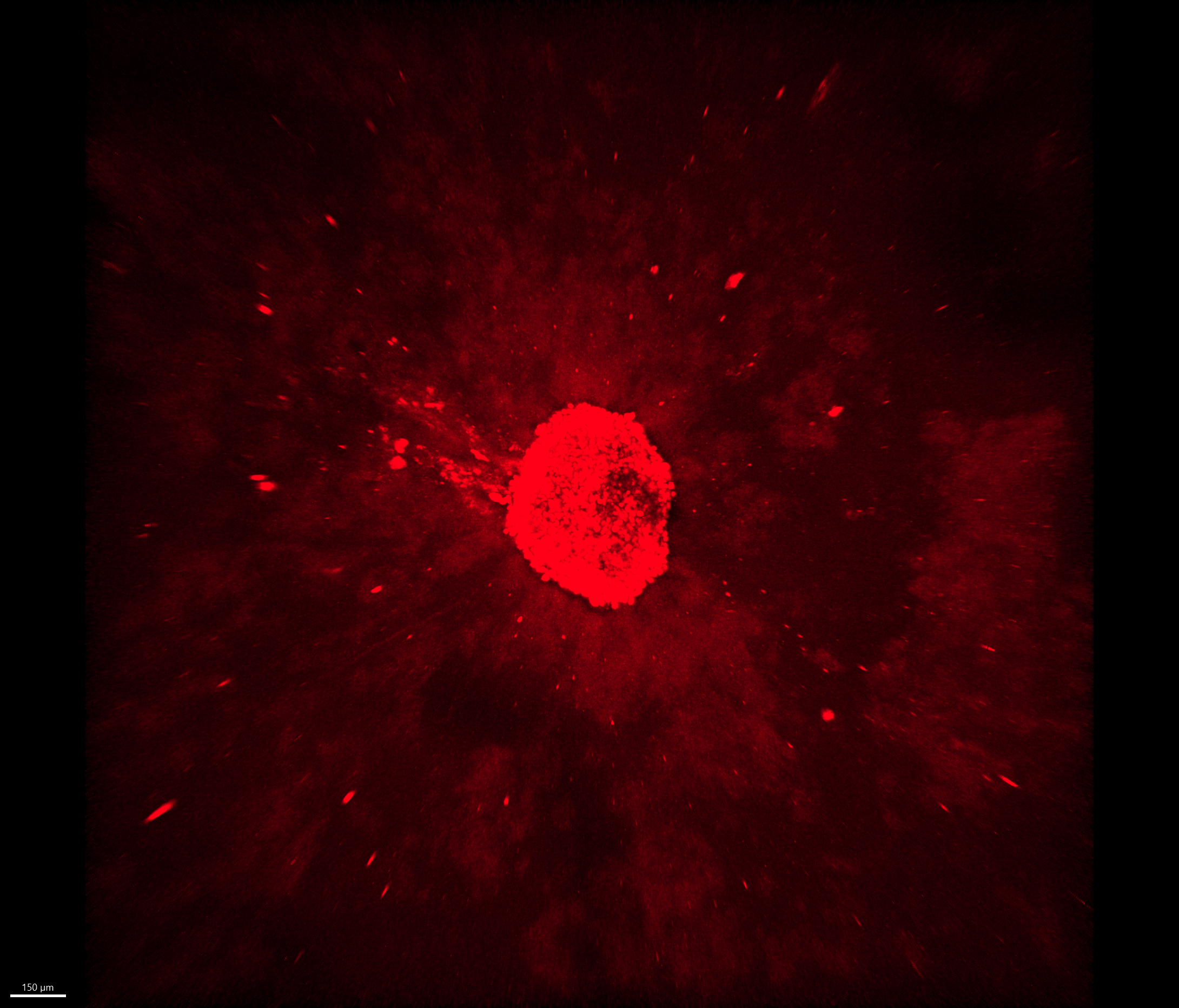

Supplement: Supplementary file 1 — Supplementary Information 1. [file 41598_2023_28078_MOESM1_ESM.zip › Supplementary Data S1/Imaris original images/day 2 (T=48h)/MCF7 GFP 1_[ims1_2021-05-17T11-18-07.782]_2021-05-17T11-32-23.550.tif]

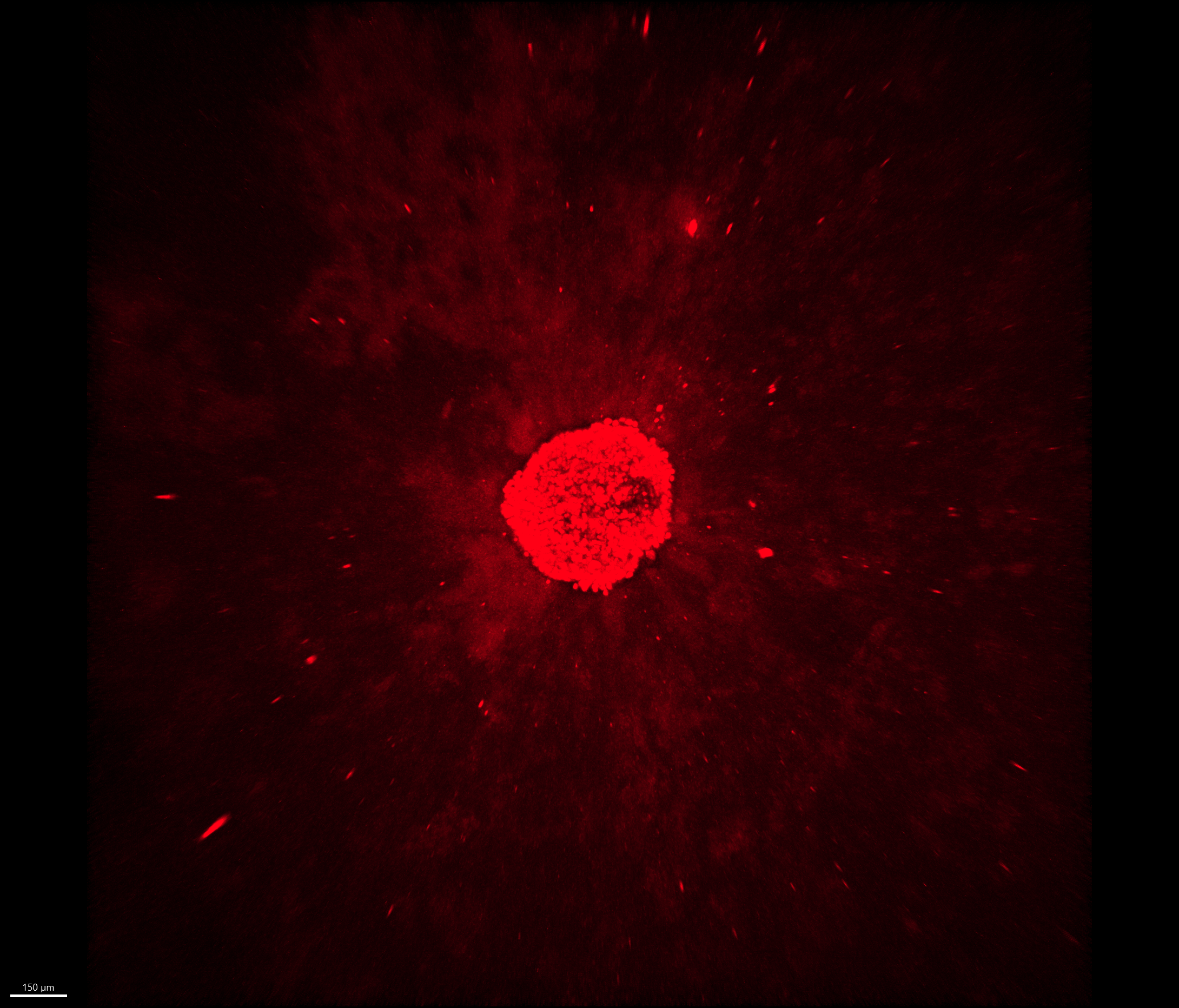

Supplement: Supplementary file 1 — Supplementary Information 1. [file 41598_2023_28078_MOESM1_ESM.zip › Supplementary Data S1/Imaris original images/day 2 (T=48h)/MCF7 GFP 2_[ims1_2021-05-17T11-18-07.782]_2021-05-17T11-32-55.496.tif]

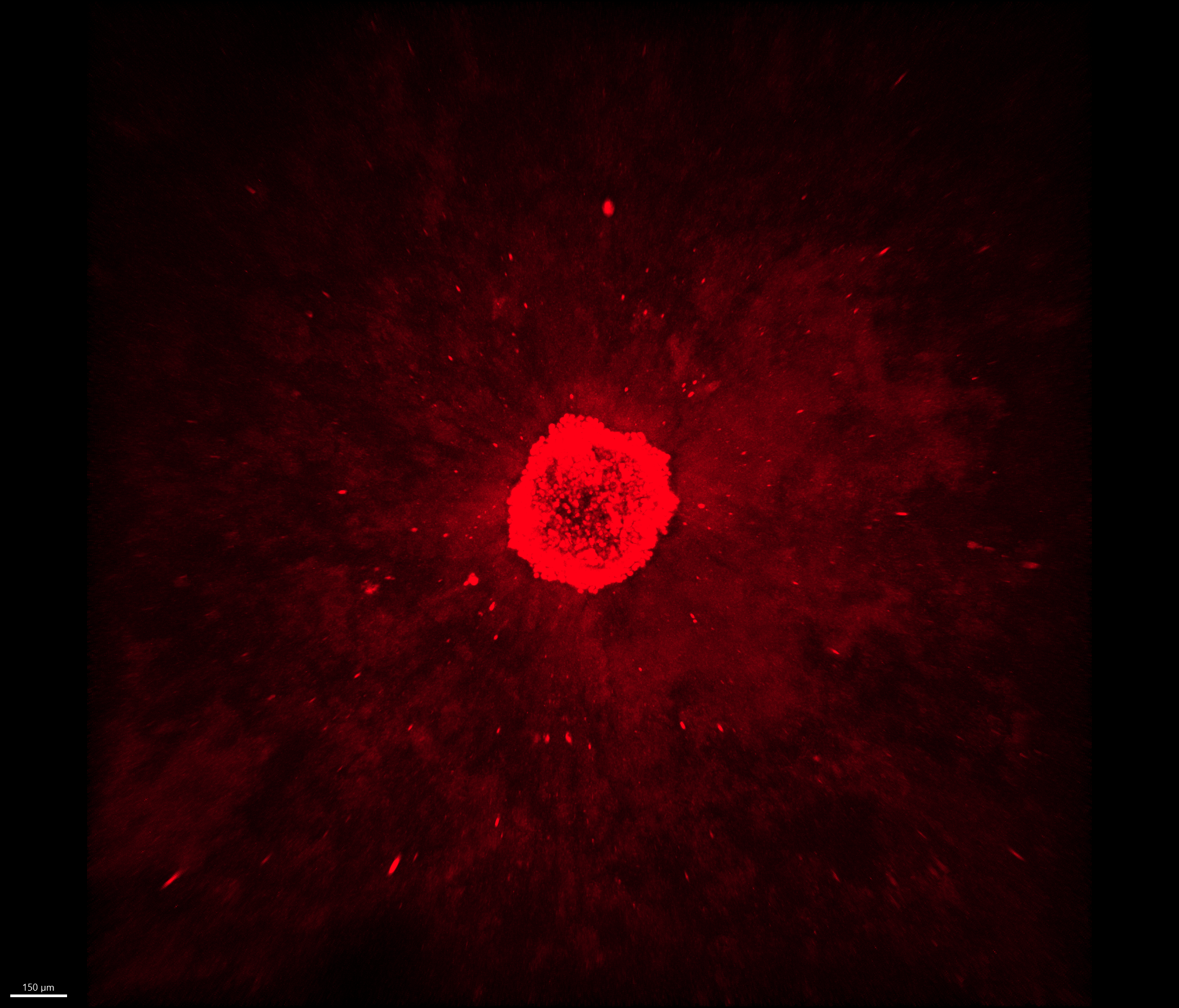

Supplement: Supplementary file 1 — Supplementary Information 1. [file 41598_2023_28078_MOESM1_ESM.zip › Supplementary Data S1/Imaris original images/day 2 (T=48h)/MCF7 GFP 3_[ims1_2021-05-17T11-18-07.782]_2021-05-17T11-33-22.504.tif]

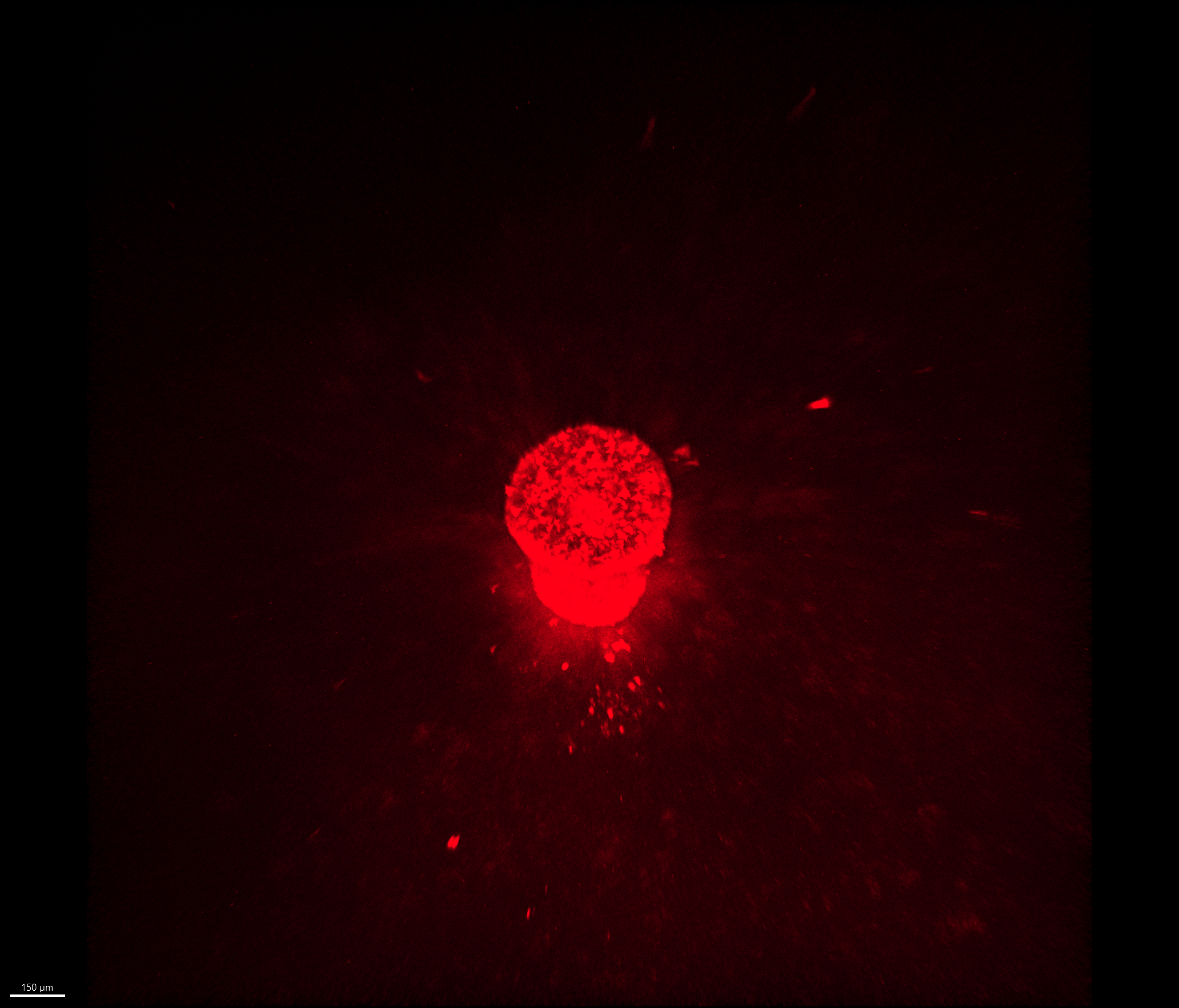

Supplement: Supplementary file 1 — Supplementary Information 1. [file 41598_2023_28078_MOESM1_ESM.zip › Supplementary Data S1/Imaris original images/day 3 (T=72h)/MCF7 COMT 1_[ims1_2021-05-17T11-20-01.133]_2021-05-17T11-34-44.691.tif]

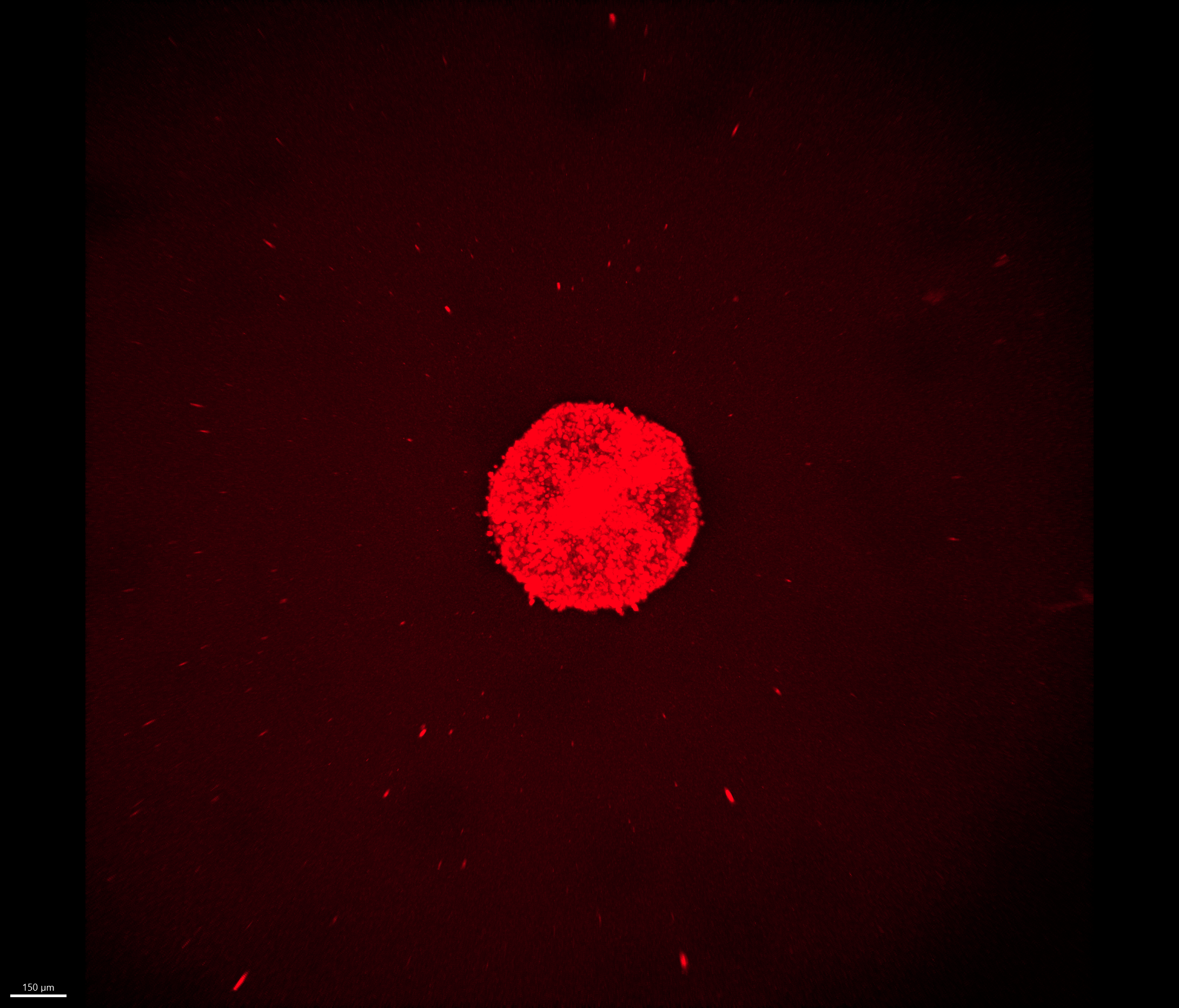

Supplement: Supplementary file 1 — Supplementary Information 1. [file 41598_2023_28078_MOESM1_ESM.zip › Supplementary Data S1/Imaris original images/day 3 (T=72h)/MCF7 COMT 2_[ims1_2021-05-17T11-20-01.133]_2021-05-17T11-35-18.553.tif]

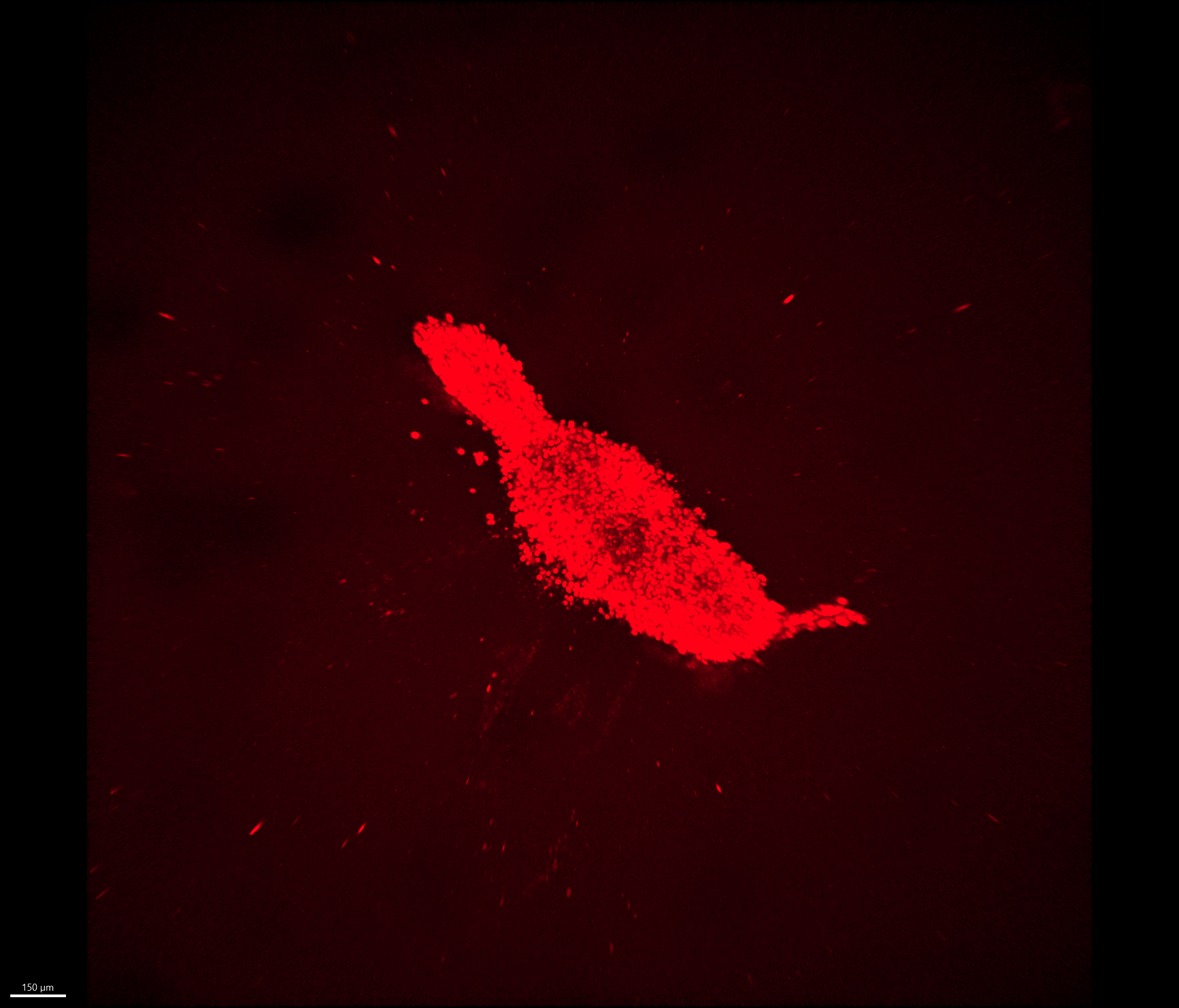

Supplement: Supplementary file 1 — Supplementary Information 1. [file 41598_2023_28078_MOESM1_ESM.zip › Supplementary Data S1/Imaris original images/day 3 (T=72h)/MCF7 COMT 3_[ims1_2021-05-17T11-20-01.133]_2021-05-17T11-35-46.102.tif]

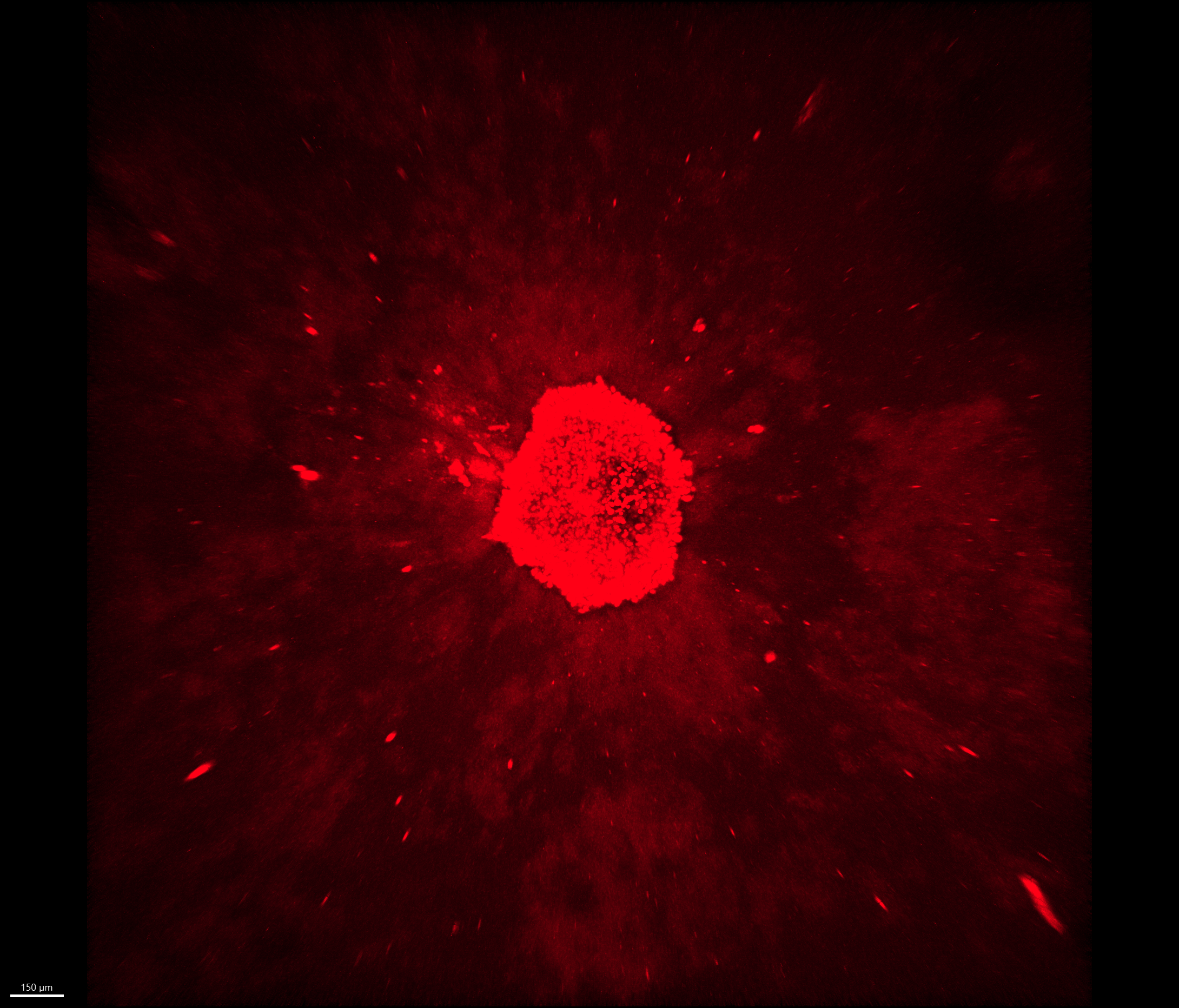

Supplement: Supplementary file 1 — Supplementary Information 1. [file 41598_2023_28078_MOESM1_ESM.zip › Supplementary Data S1/Imaris original images/day 3 (T=72h)/MCF7 GFP 1_[ims1_2021-05-17T11-20-01.133]_2021-05-17T11-37-23.710.tif]

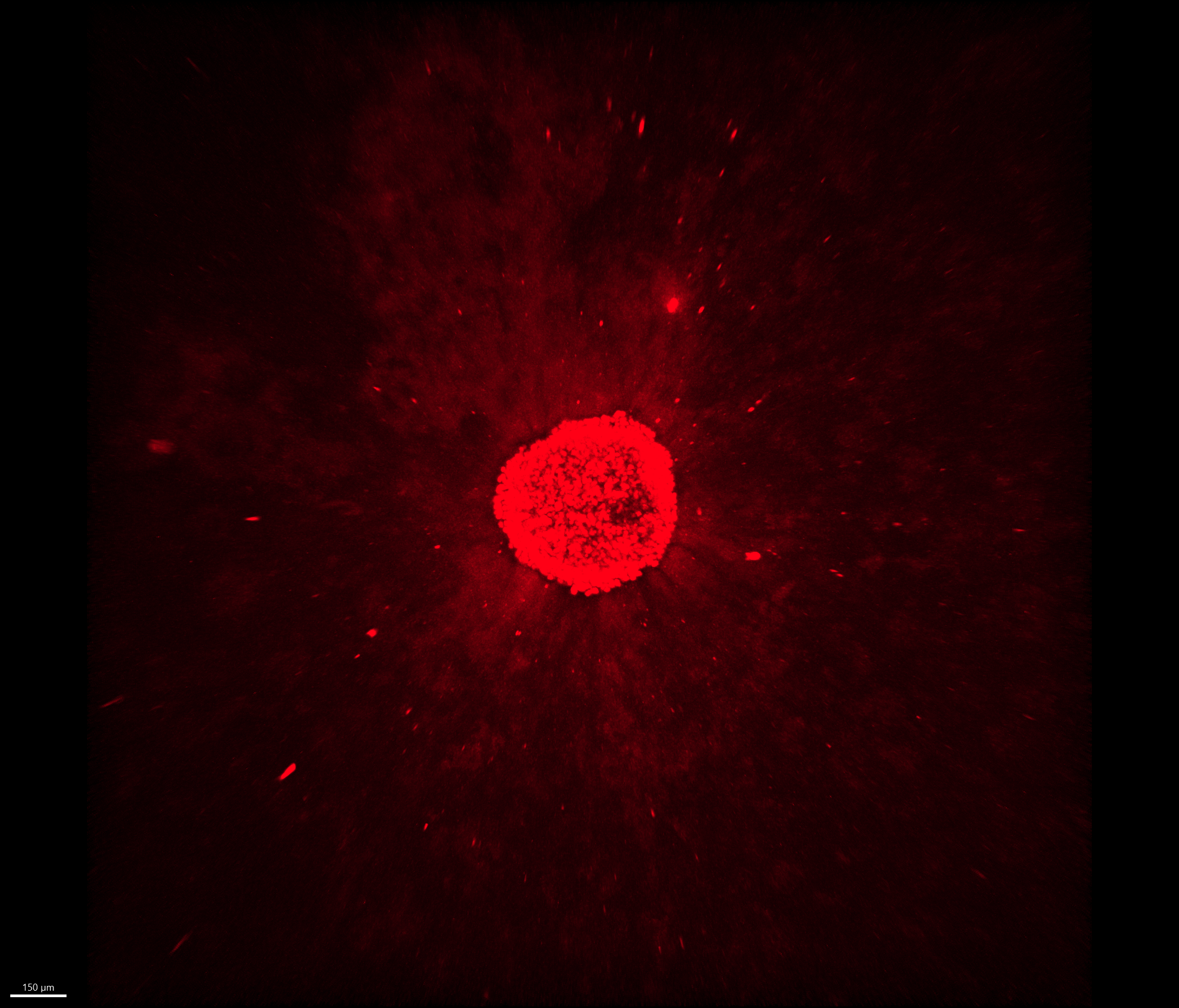

Supplement: Supplementary file 1 — Supplementary Information 1. [file 41598_2023_28078_MOESM1_ESM.zip › Supplementary Data S1/Imaris original images/day 3 (T=72h)/MCF7 GFP 2_[ims1_2021-05-17T11-20-01.133]_2021-05-17T11-38-20.595.tif]

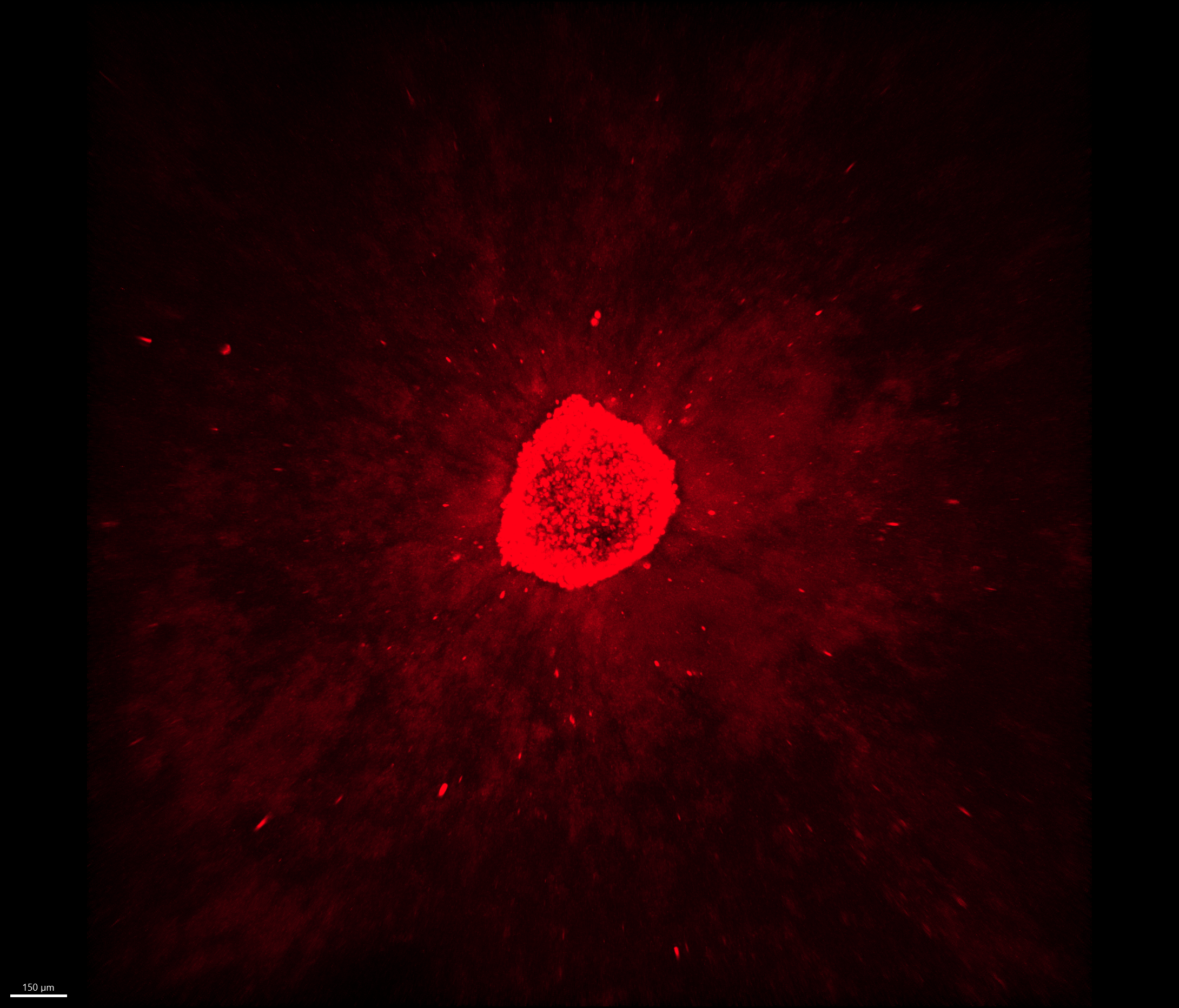

Supplement: Supplementary file 1 — Supplementary Information 1. [file 41598_2023_28078_MOESM1_ESM.zip › Supplementary Data S1/Imaris original images/day 3 (T=72h)/MCF7 GFP 3_[ims1_2021-05-17T11-20-01.133]_2021-05-17T11-38-51.058.tif]

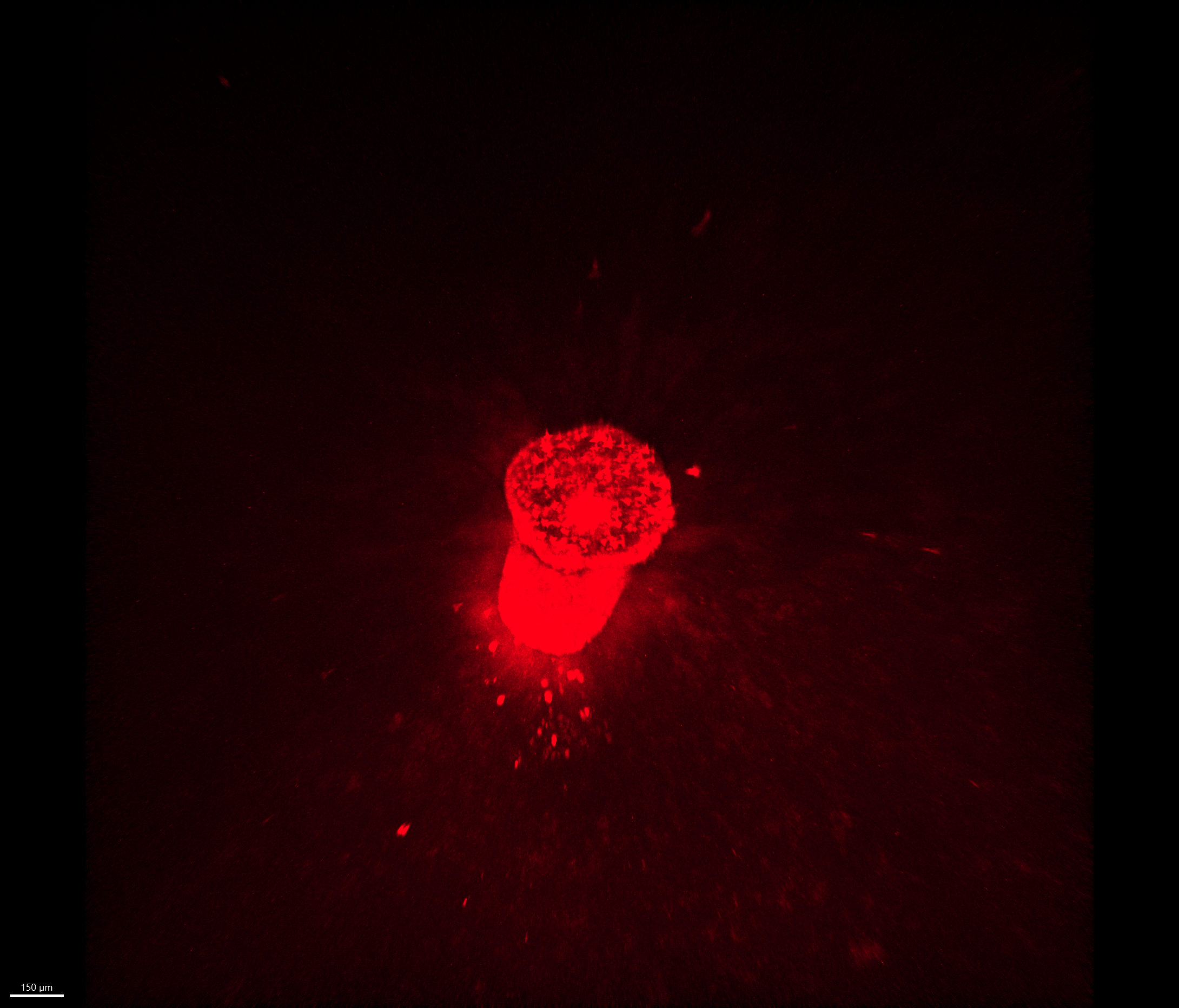

Supplement: Supplementary file 1 — Supplementary Information 1. [file 41598_2023_28078_MOESM1_ESM.zip › Supplementary Data S1/Imaris original images/day 4 (T=96h)/MCF7 COMT 1_[ims1_2021-05-17T11-21-37.449]_2021-05-17T11-39-31.311.tif]

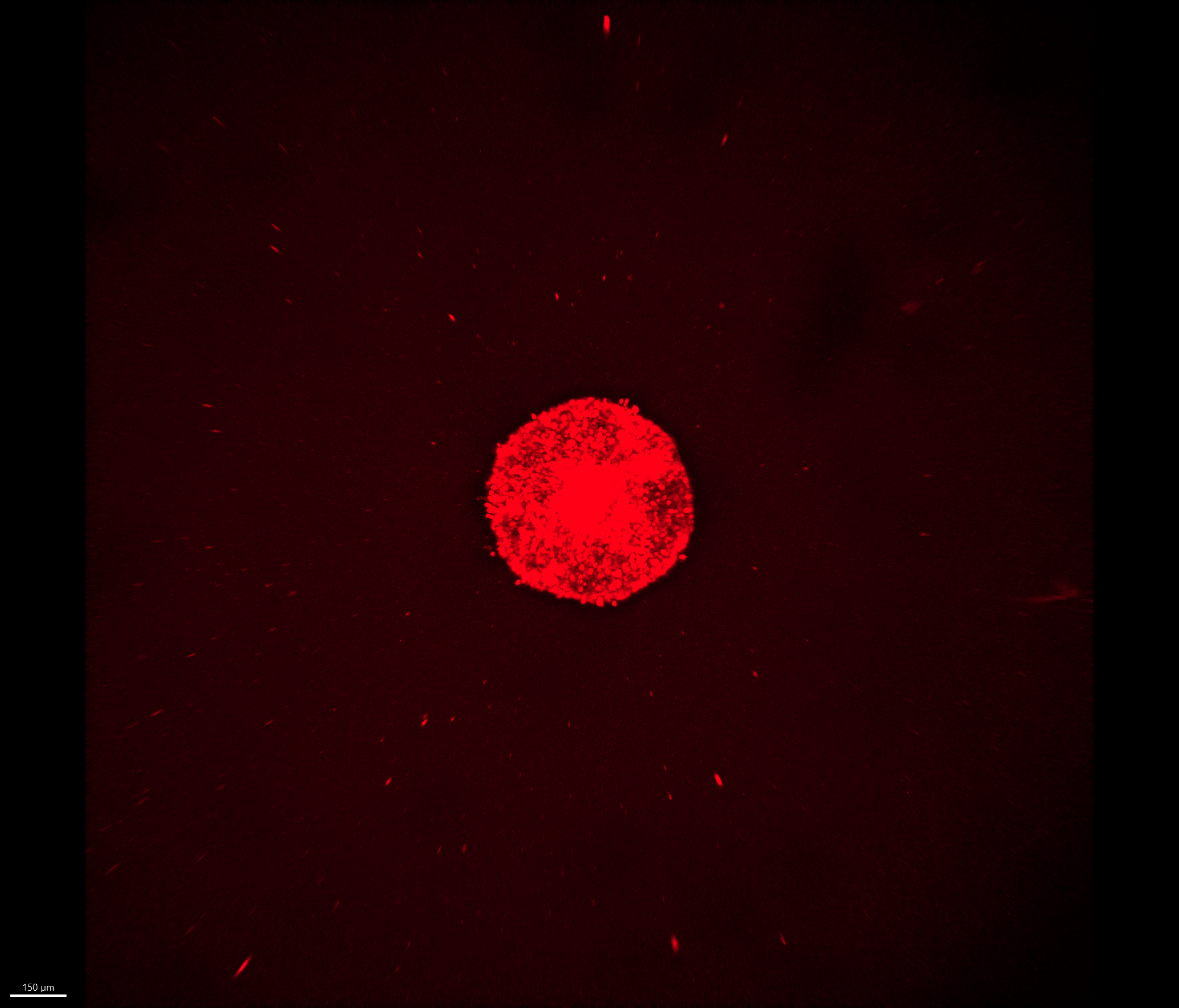

Supplement: Supplementary file 1 — Supplementary Information 1. [file 41598_2023_28078_MOESM1_ESM.zip › Supplementary Data S1/Imaris original images/day 4 (T=96h)/MCF7 COMT 2_[ims1_2021-05-17T11-21-37.449]_2021-05-17T11-40-16.926.tif]

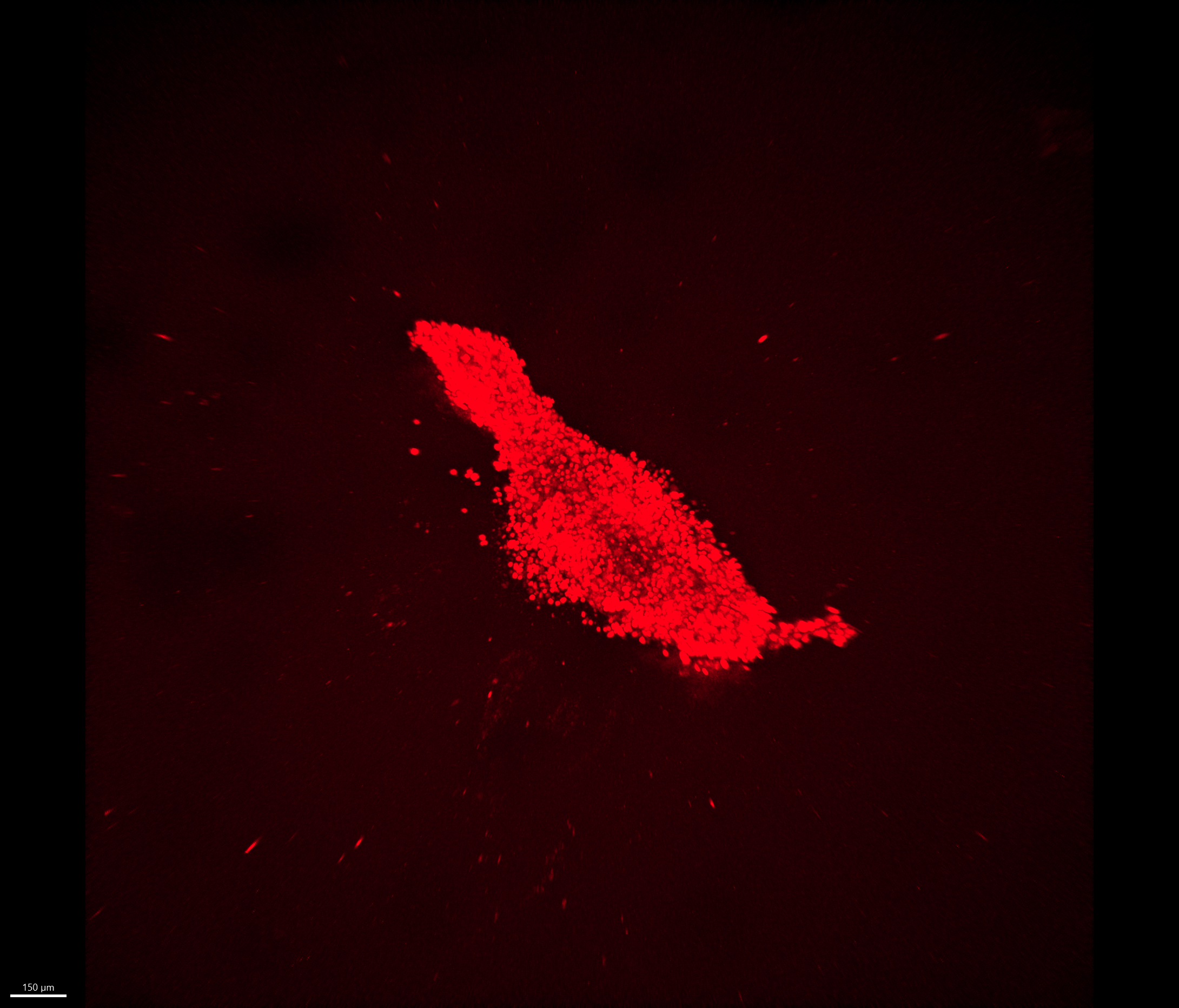

Supplement: Supplementary file 1 — Supplementary Information 1. [file 41598_2023_28078_MOESM1_ESM.zip › Supplementary Data S1/Imaris original images/day 4 (T=96h)/MCF7 COMT 3_[ims1_2021-05-17T11-21-37.449]_2021-05-17T11-40-48.050.tif]

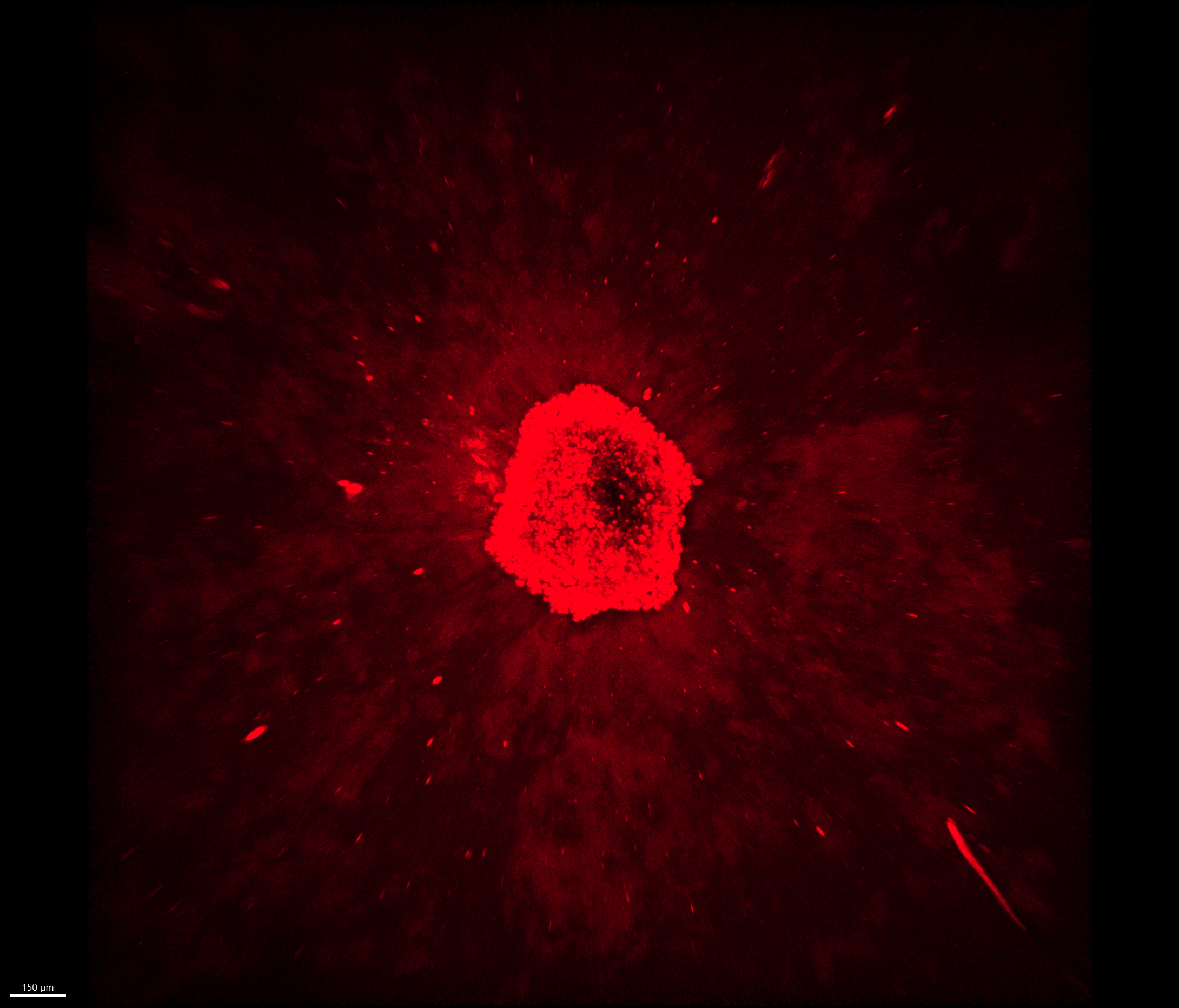

Supplement: Supplementary file 1 — Supplementary Information 1. [file 41598_2023_28078_MOESM1_ESM.zip › Supplementary Data S1/Imaris original images/day 4 (T=96h)/MCF7 GFP 1_[ims1_2021-05-17T11-21-37.449]_2021-05-17T11-41-14.981.tif]

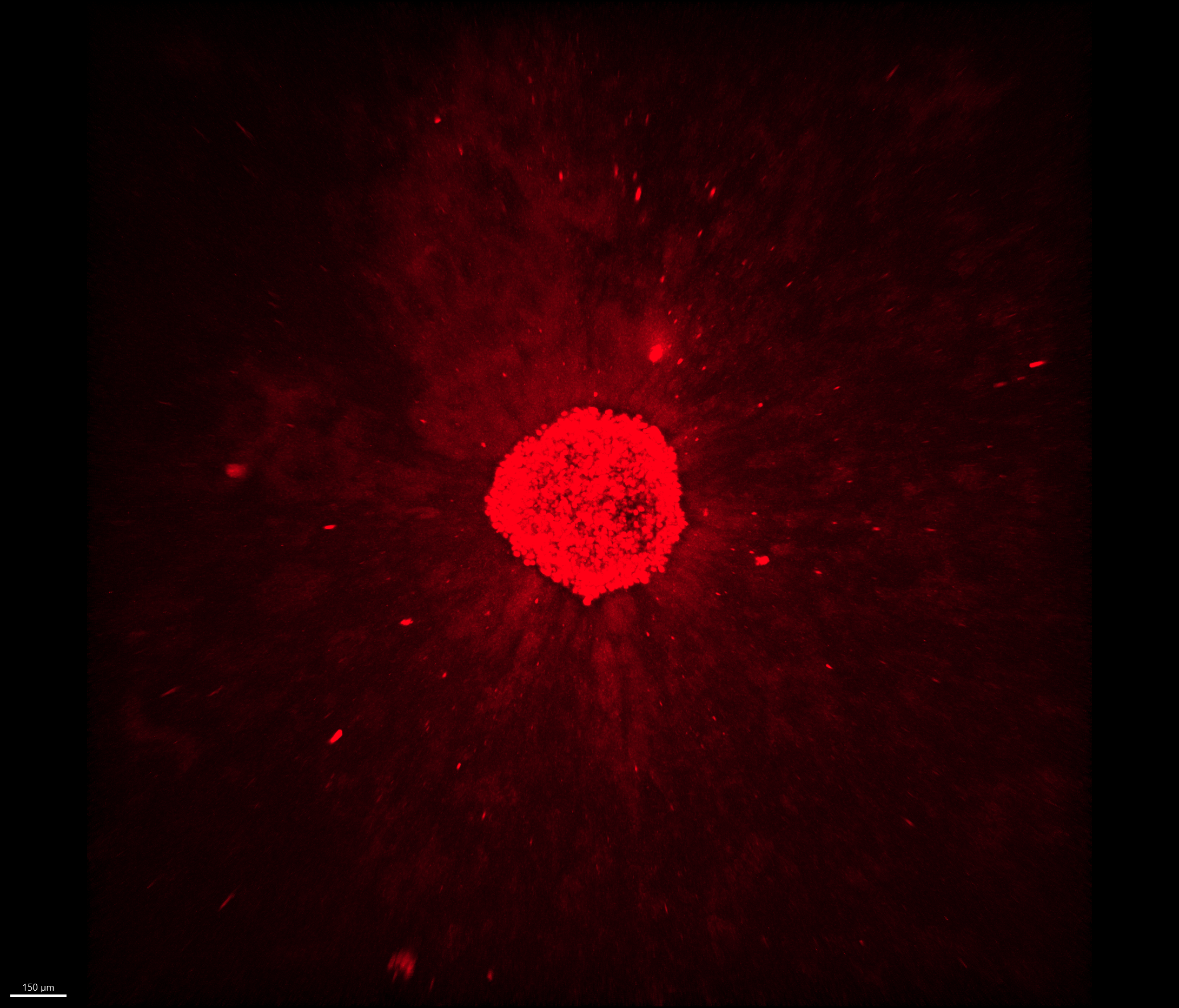

Supplement: Supplementary file 1 — Supplementary Information 1. [file 41598_2023_28078_MOESM1_ESM.zip › Supplementary Data S1/Imaris original images/day 4 (T=96h)/MCF7 GFP 2_[ims1_2021-05-17T11-21-37.449]_2021-05-17T11-41-55.925.tif]

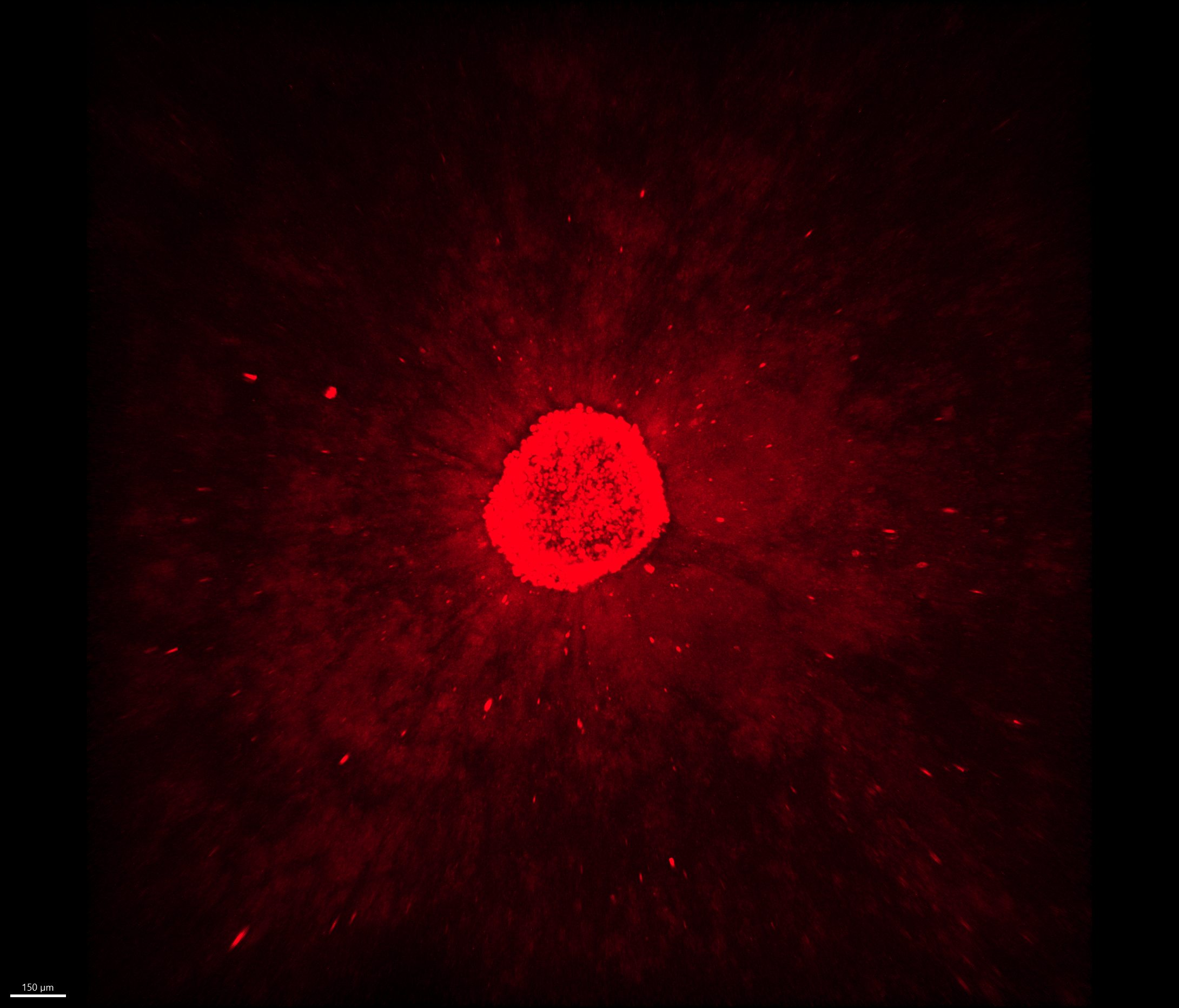

Supplement: Supplementary file 1 — Supplementary Information 1. [file 41598_2023_28078_MOESM1_ESM.zip › Supplementary Data S1/Imaris original images/day 4 (T=96h)/MCF7 GFP 3_[ims1_2021-05-17T11-21-37.449]_2021-05-17T11-42-23.413.tif]
