# Supplementary material for: Catechol-O-methyl transferase suppresses cell invasion and interplays with MET signaling in estrogen dependent breast cancer
Source: Sci Rep. 2023 Jan 23;13:1285. doi: 10.1038/s41598-023-28078-1 (PMC9870911; doi:10.1038/s41598-023-28078-1)
Supplement: Supplementary file 8 — Supplementary Information 8. [file 41598_2023_28078_MOESM8_ESM.docx]

**Supplementary Figures**


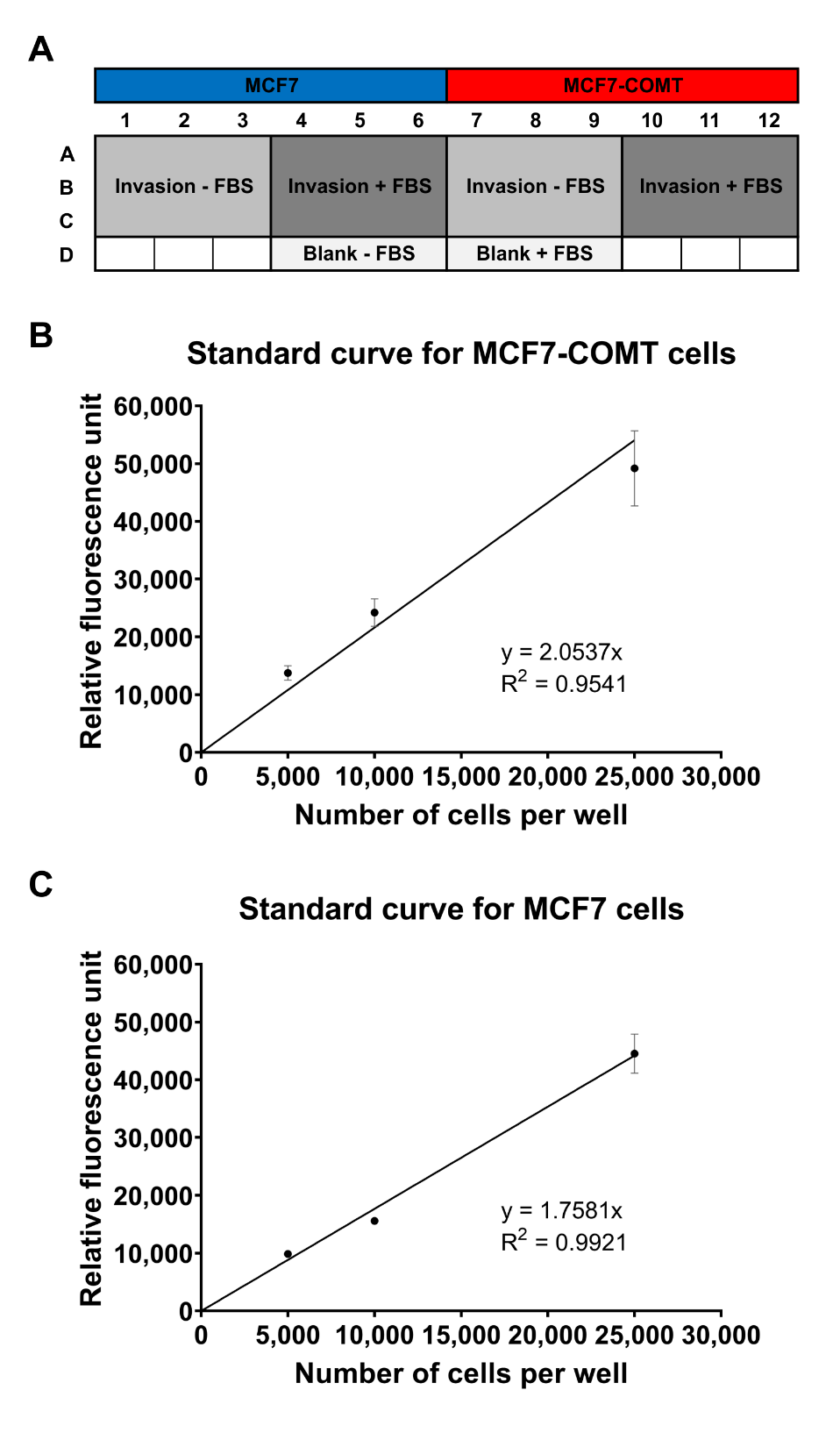


**Figure S1 (related to Fig. 1)**: **Details of Transwell invasion assay**. Design of Transwell plate (A). Invasiveness with and without chemoattractant (FBS) was measured in nine wells per condition (MCF7-COMT and MCF7 cells, respectively). Blank wells without the cells (medium with or without FBS) were measured for background subtraction in triplicates. Standard curves for MCF7-COMT cells (B) and MCF7 control cells (C) were used for calculation of percentual invasiveness compared to initially seeded cells.


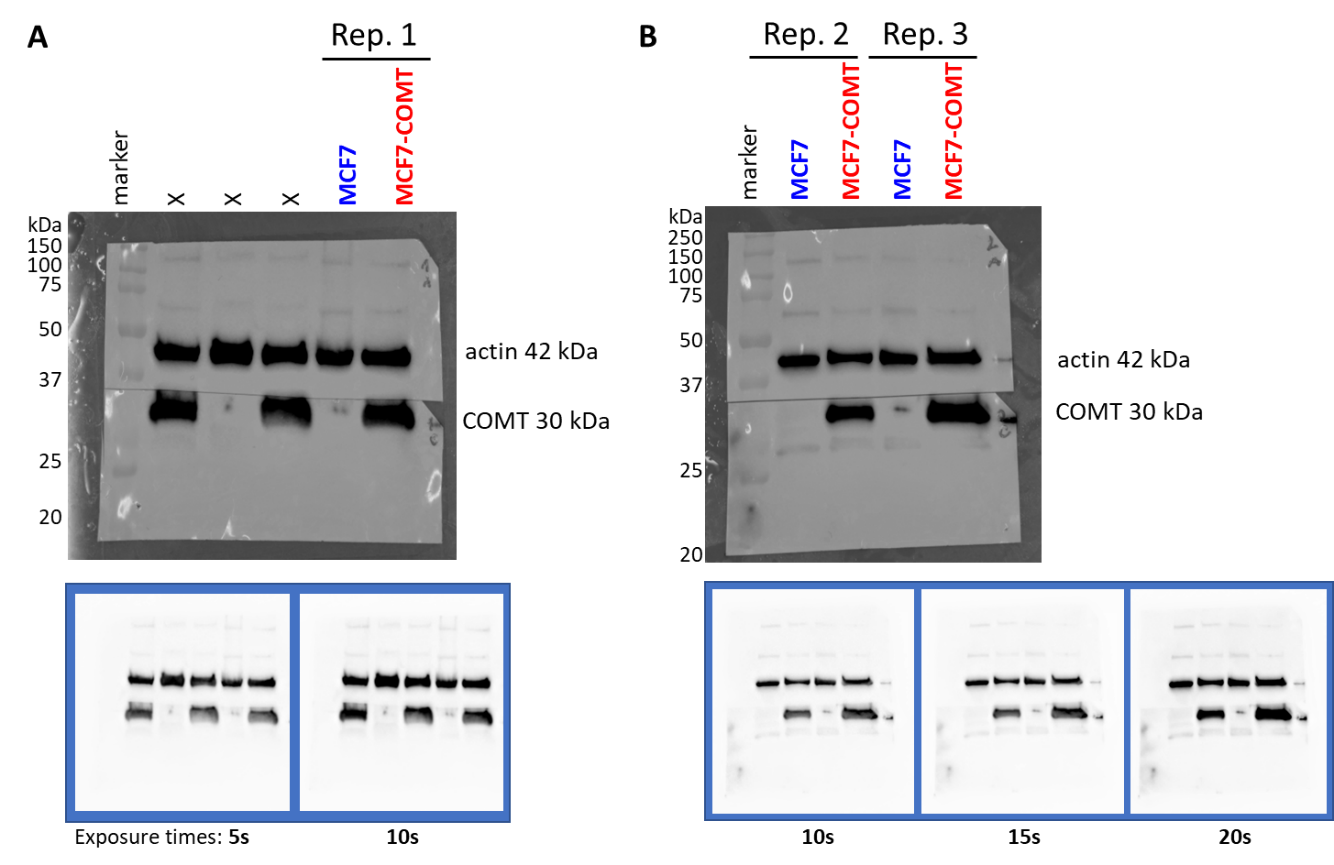


**Figure S2 (related to Fig. 1): Raw immunoblotting image of COMT and actin in COMT overexpressing MCF7 cells (MCF7-COMT) compared to control parental MCF7 cells used in Transwell assay.** (A) Replicate 1 image merged with marker and raw images of two exposure times and (B) Replicates 2 and 3 image merged with marker and raw images of three exposure times.

**
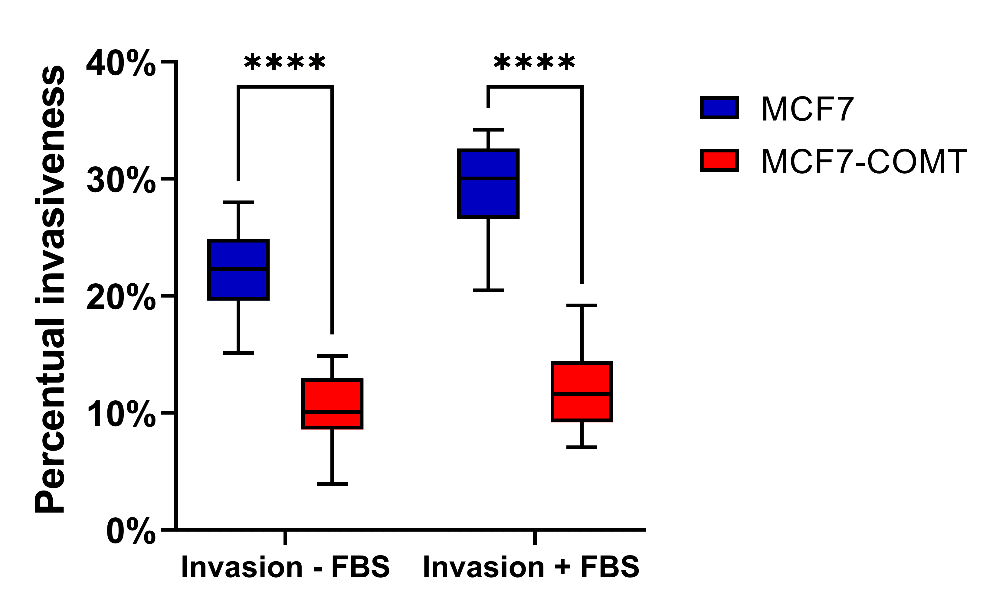
**

**A**

**B**

**
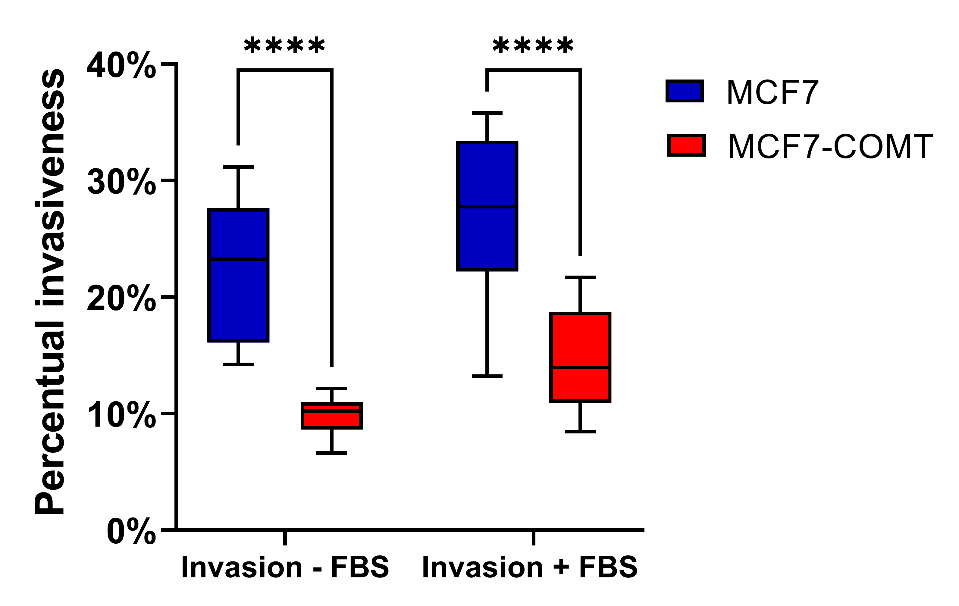
**

**C**

**
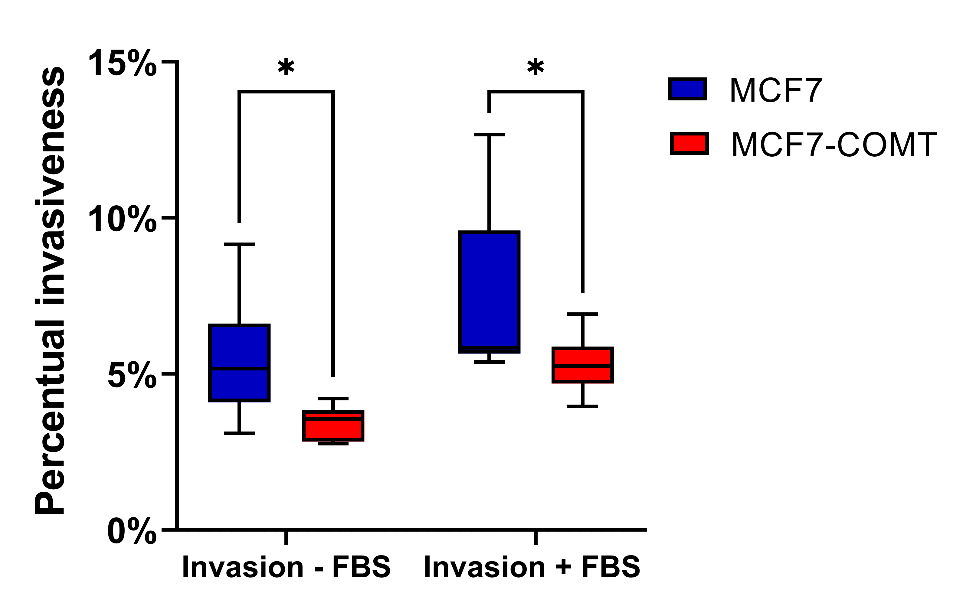
**

**Figure S3 (related to Fig. 1)**: **Transwell invasion assay of COMT overexpressing MCF7 cells (MCF7-COMT) compared to parental MCF7 cells, three independent experiments.** Box plot of the percentual invasiveness of the cells with and without the presence of chemoattractant (fetal bovine serum, FBS) (n=9 per cell line). **** p<0.0001, *** p<0.001, * p<0.05. The box extends from the 25th to 75th percentiles, the line in the middle of the box is plotted at the median. The whiskers represent minimal and maximal value of the dataset. See Fig. S1 for the design of Transwell assay plate and calibration curves.

**
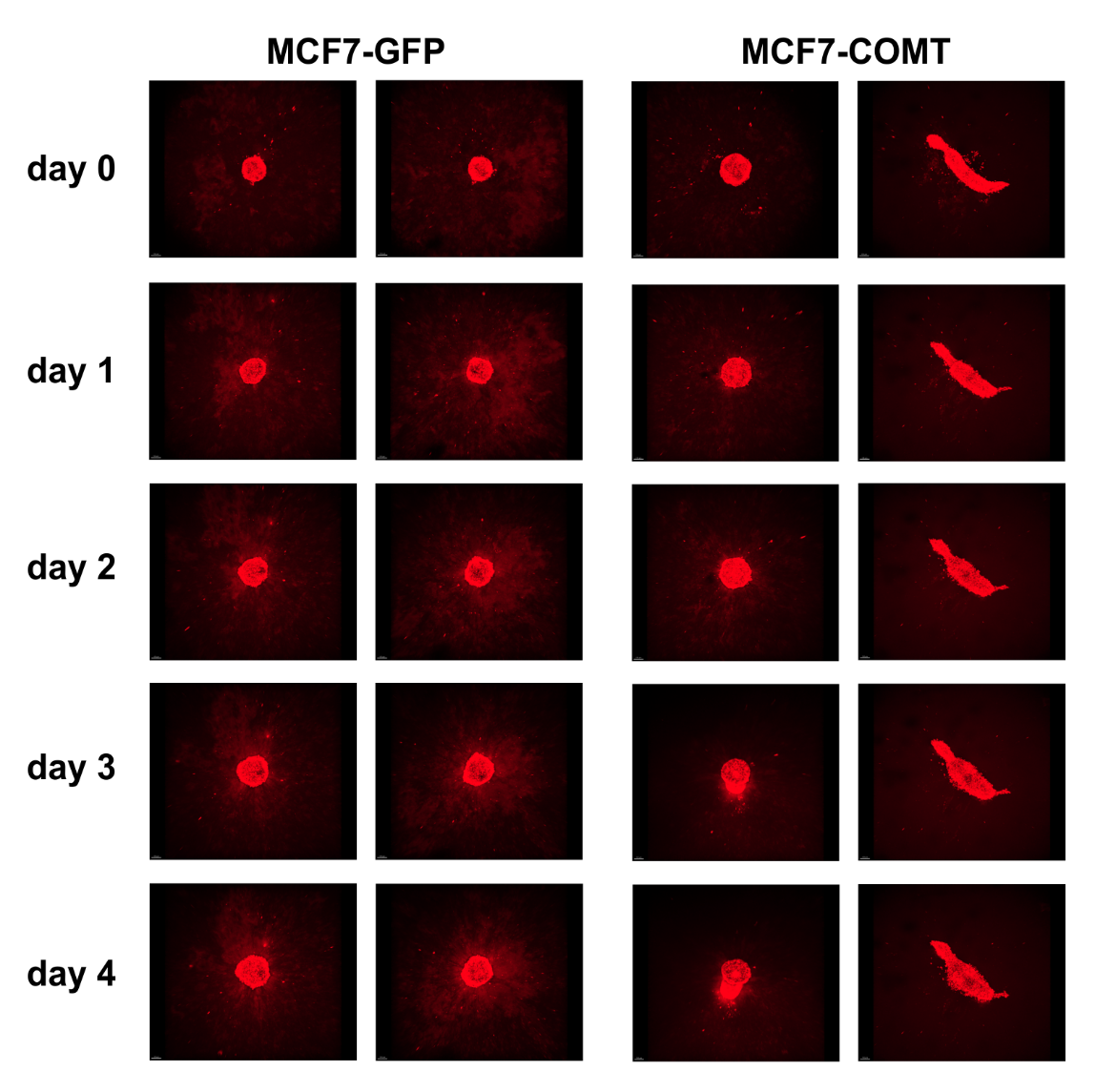
**

**Figure S4 (related to Fig. 2). 3D spheroid invasion assay of COMT overexpressing MCF7 cells (MCF7-COMT) compared to control MCF7-GFP cells.** Z-stack images of spheroids formed by MCF7-COMT cells and control cell line have been acquired by confocal laser scanning microscopy for 5 days. Data for two remaining replicates.


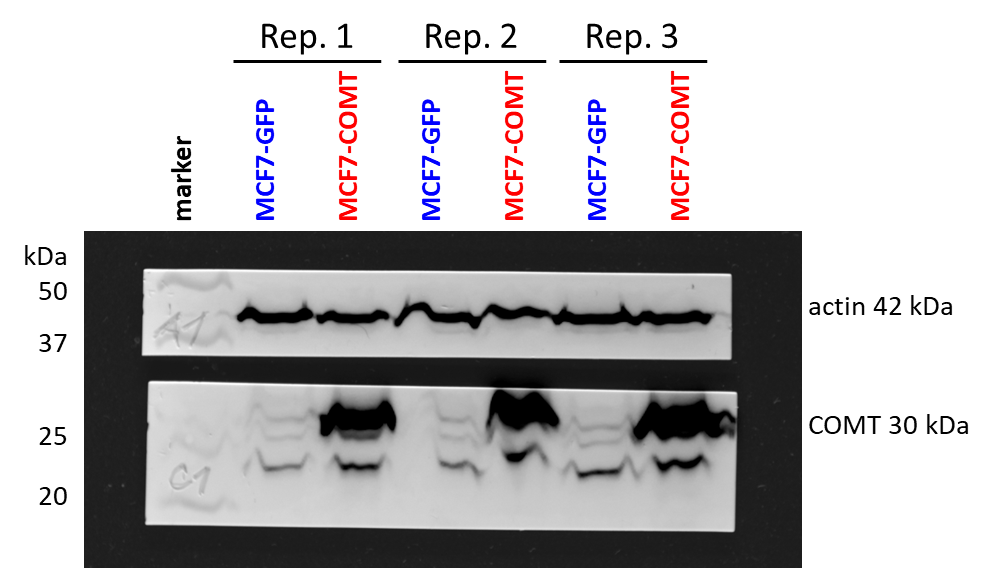


**Figure S5 (related to Fig. 2): Raw immunoblotting image of COMT and actin in COMT overexpressing MCF7 cells (MCF7-COMT) compared to control MCF7-GFP cells used in 3D invassion assay.**


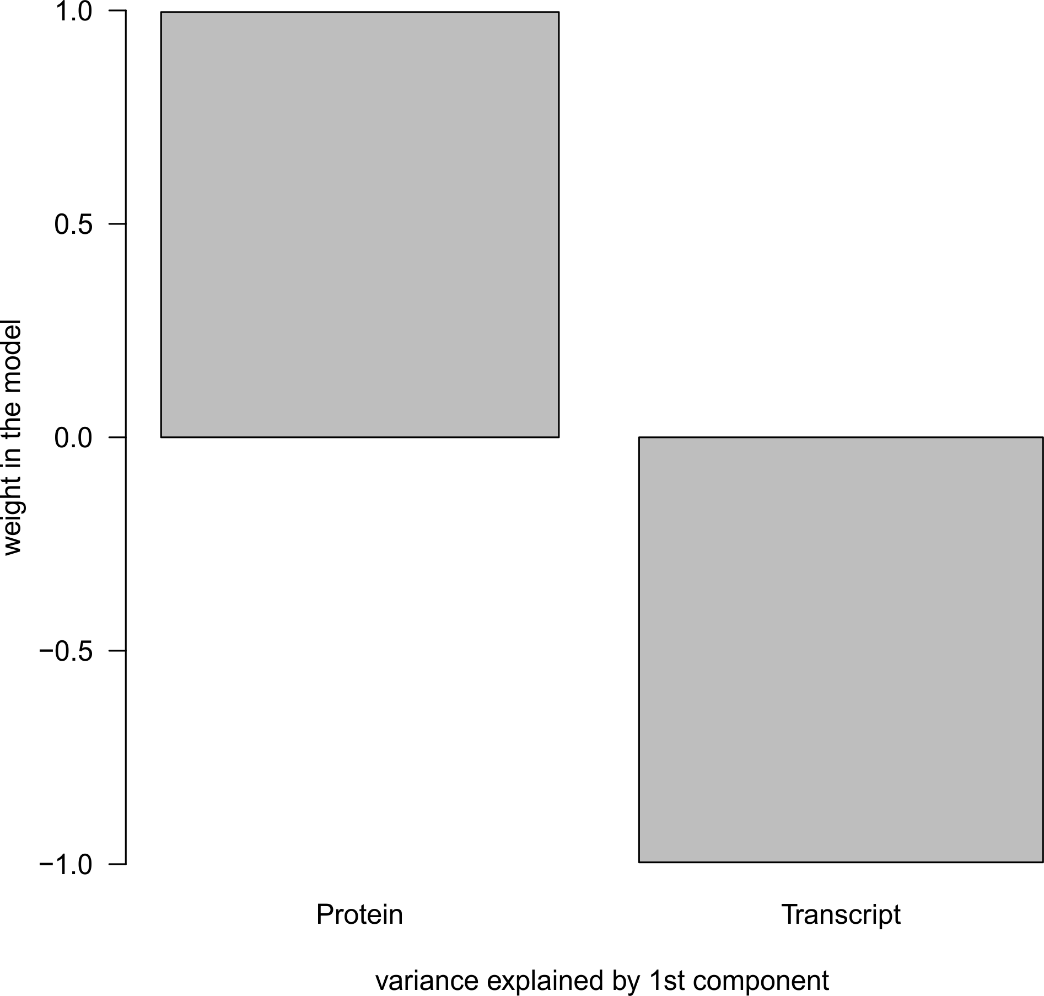


**Figure S6 (related to Fig. 5): Variance between MCF7-COMT samples and MCF7-GFP control cells in RNA-Seq and proteomics data.** By combining our ‘omics analyses using multivariate methods we gained a sufficient insight to distinguish the effect of COMT overexpression on MCF7 cells solely using any of the datasets.


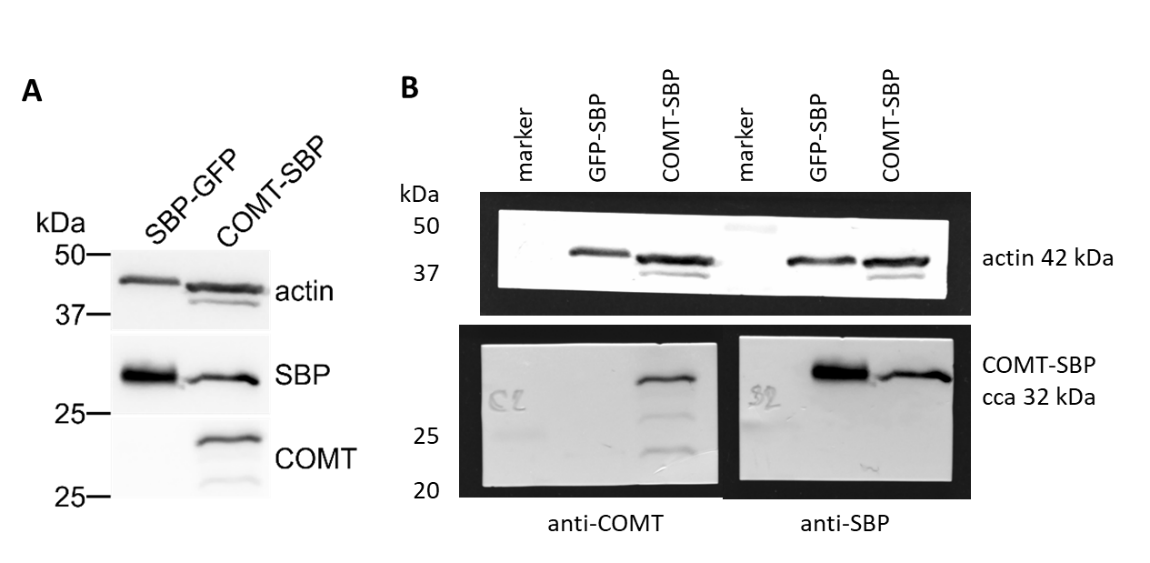


**Figure S7 (related to pull-down assay): Input protein levels of COMT, SBP and actin in MCF7-SBP-COMT and control MCF7-SBP-GFP cells used for pull-down analysis of COMT interaction partners.** (A) processed images and (B) raw images of immunoblotting.

**Supplementary Methods**

**Molecular cloning**

The coding sequence of COMT was transcribed and amplified using SuperScript III One-Step RT-PCR System according to manufacturer protocol (Invitrogen, MAN0001093) from 100 ng of total RNA isolated of MDA-MB-231 cells (obtained by RNeasy Mini Kit; Qiagen, Germany) with COMT specific primers: COMT-mRNA-F 5´-CATCGTCGTGGGGCTTCTG-3´ and COMT-mRNA-R 5´-GCAGGGGAATGGCAGTTGAGAA-3´. Gateway technology (Thermo Fisher Scientific, USA) was used to construct an entry plasmid carrying the COMT gene. The cDNA of COMT was amplified using PCR with Herculase II DNA polymerase (Agilent, 600675-12), pair of COMT cDNA specific primers with TEV cleavage site: COMT-MB(CDS)-TEV-F: 5´- GGCTCTGAGAACCTGTACTTCCAGAGCATGCCGGAGGCCCCGCCTC-3´, COMT-MB(CDS)-GWs-R: 5´- GTACAAGAAAGCTGGGTTTCAGGGCCCTGCTTCGCT-3´ and pair of Gateway universal primers: GW-TEV-F: 5´-GGGGACAAGTTTGTACAAAAAAGCAGGCTTCGGCTCTGAGAACCTGTACTTC-3´ and GW-attb2-R: 5´-GGGGACCACTTTGTACAAGAAAGCTGGGTT-3´. The reaction mixture contained 1 M betaine, 20 μl cDNA, 1x reaction buffer, 1 μM dNTP, 1 μl Herculase II, 0.1 μM specific primers and 0.3 μM universal primers, nuclease-free water was added to a final volume of 50 μl. The PCR program included pre-denaturation at 95 °C/1 min, 30 amplification cycles - denaturation at 95 °C/10 s, annealing at 50 °C/20 s, elongation at 70 °C/90 s, followed by final elongation at 70 °C/5 min. PCR product was separated by electrophoresis on 1% GelRed^TM^ stained agarose gel, visualized under UV-light by CCD camera and the target attB product (816 bp) was extracted by QIAquick Gel Extraction Kit (Qiagen) according to the manufacturer protocol. BP recombinant reaction between 816 bp attB-product and pDONR221 plasmid was done according to Gateway technology manual (Thermo Fisher Scientific, USA, MAN0000282). Chemically competent E. coli TOP10 (Life Technologies) cells were used for the preparation and amplification of resulting entry vectors pENTR221-COMT. QIAprep Spin Miniprep Kit (Qiagen) was used for vector purification according to the manufacturer's protocol. Lentiviral vectors expressing COMT were created by gateway LR recombination reaction according to the Gateway technology manual (Thermo Fisher Scientific, USA, MAN0001032). pENTR221-COMT was used to prepare lentiviral vectors pLENTI6.3-N-HA-COMT-IRES-EmGFP-GW-DEST and pLENTI-N-SBP-COMT-IRES-GFP-GW-DEST. Chemically competent E. coli Stbl3 cells (Thermo Fisher Scientific, USA) were used for the preparation and amplification of resulting lentiviral vectors that were subsequently purified by Plasmid Maxi Kit (Qiagen).

**Construction of the stably transduced cell lines**

The resulting lentiviral vectors were used for the production of lentiviruses. The vector contains an internal ribosomal entry site (IRES) that allows simultaneous expression (translation) of COMT and EmGFP separately but from the same RNA transcript. EmGFP was used as a selection marker to separate the positively transduced cells using cell sorter BD FACSAria III. As a control, lentiviruses carrying pLENTI 6.3-EmGFP-GWs and pLENTI6.3-N-SBP-EmGFP-GWs were prepared. Production of lentiviruses, transduction of MCF7 cells, and selection of stably transfected clones were done according to the ViraPower™ Lentiviral Expression Systems user manual (Invitrogen, USA, 25-0501 MAN0000273).

**LC-MS/MS analysis of pull-down samples**

The peptides originating form pull-down samples were dissolved in 25 µl of analysis of the sample solution containing 0.05% TFA in 5% ACN and 95% water. Prior to LC-MS analysis, a mixture of synthetic peptides as retention time standards (HRM Calibration Kit, Biognosys, Switzerland) was added to the samples: 50 μl of dissolution buffer were added to the HRM peptide mixture, the mixture was then vortexed and sonicated for 5 min. The mixture was added to the prepared samples at 1:10 ratio. Separation of tryptic peptides was performed on an Eksigent Ekspert nanoLC 400 liquid chromatograph (SCIEX, Dublin, California), which was connected online to a TripleTOF 5600+ mass spectrometer (SCIEX, Toronto, Canada). Pre-concentration and additional desalting of the samples were performed using a mobile phase consisting of 0.05% TFA in 5% ACN and 95% water on a cartridge capture column (300 μm id x 5 mm) filled with C18 PepMap100 sorbent on 5 μm particles (Thermo Fisher Scientific, Waltham, MA, USA). The peptides were then separated by an ACN/water gradient with a flow rate of 300 nl/min on an in-house capillary analytical PicoFrit® nanospray column (75 μm x 250 mm, New Objective, USA) filled with the ProntoSIL 120-3-C18 AQ stationary phase with 3 μm particles (Bischoff, Germany). Mobile phase A comprised 0.1% (v/v) formic acid (FA) in water and the mobile phase B consisted of 0.1% (v / v) FA in ACN. The analytical gradient was set at 5% of mobile phase B and 95% of mobile phase A, and the ratio was increased to 40% B and 60% A during 120 minutes. The separated peptides were ionized using a nano-electrospray (nitrogen used as mist and drying gas, flow rate 12, emitter voltage 2.65 kV) using DIA mode in high sensitivity mode. The precursor range was set from 400 to 1200 Th and was divided into 67 precursor windows with a width of 12 Th and an overlap of 1 Th. The accumulation time per precursor window was 50.8 ms, the total cycle time was 3.5 s. The MS/MS spectra were scanned from 360 to 1360 Th.
